# Oxanorbornenes: promising new single addition monomers for the metathesis polymerization†

**DOI:** 10.1039/d1sc00036e

**Published:** 2021-04-07

**Authors:** Subhajit Pal, Mahshid Alizadeh, Phally Kong, Andreas F. M. Kilbinger

**Affiliations:** Department of Chemistry, University of Fribourg Chemin du Musée 9 1700 Fribourg Switzerland andreas.kilbinger@unifr.ch

## Abstract

Higher ring-opening metathesis propagation rates of *exo*-norbornene derivatives over *endo* derivatives are well established in the literature. Here, we report for the first time that *endo*-isomers of oxanorbornene derivatives show higher reactivity towards ring-opening metathesis with Grubbs' 3rd generation catalyst (**G3**) than the corresponding *exo*-isomers. A very high selectivity for the reaction of **G3** with *endo* over the *exo*-isomers could be shown. Furthermore, single molecular addition of the *endo*-isomers with **G3** was observed. On the other hand, pure *exo*-monomers could successfully be homopolymerized. Mixtures of *exo*- and *endo*- monomers, however, prevented the homopolymerization of the *exo*-monomer. Such mixtures could successfully be copolymerized with cycloalkenes, resulting in alternating copolymers. An oxanorbornadiene derivative could be shown to undergo single addition reactions, exploited in the preparation of mono-end functional ROMP polymers. These could be selectively derivatized *via* endgroup selective thiol-ene click reactions. A thiol and alcohol end functional ROMP polymer was synthesized, and the efficient end functionalization was confirmed by ^1^H NMR spectroscopy and MALDI-ToF spectrometry.

## Abstract 2

Bridgehead revisited: *endo*-7-oxa norborneneimide derivatives (green) initiate faster but propagate more slowly than the analogous *exo*-derivatives (red) in ring-opening metathesis allowing the synthesis of alternating and end functional polymers.

## Graphical abstract

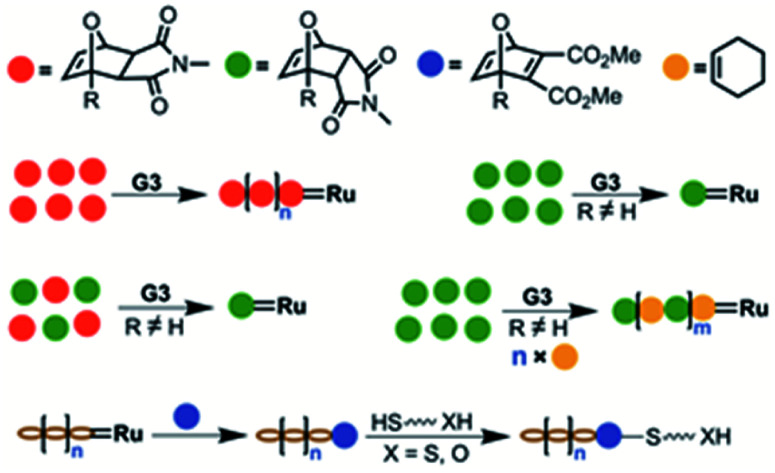

## Introduction

Today, the ring-opening metathesis polymerization (ROMP) has become a routine polymerization technique in the toolbox of polymer chemists. Typically, ruthenium and molybdenum based metal carbene complexes are used for ROMP.^1–3^ Features such as living polymerization, high functional group tolerance of metathesis catalysts, mild reaction conditions, and short reaction times make ROMP popular in polymer as well as materials chemistry.^4–7^ Release of ring strain of the cyclic olefins is the driving force for polymerization; as a result, highly strained cyclic olefins like cyclopropene, cyclobutene, cyclooctene, norbornene, *etc.* are used as monomers in ROMP.^8^

In addition to fast and living polymerizations, control over molecular structure and monomer sequence has been a central goal of polymer chemistry. Several synthetic approaches have been made to tune the polymers' microscopic properties by the precise placement of chemical functionality.^9,10^ However, a strictly sequential arrangement of individual monomer units in a polymer remains challenging.

Much progress has been made regarding the precise placement of end functional groups in ROMP polymers.^6,11^ Within the broad field of reactive polymer chain ends, the thiol group has received significant attention. From bioconjugation to transition metal complexation, thiols are often superior over their oxo-derivatives.^12,13^ One of the most common approaches for ROMP polymer end functionalization is the chain transfer with a symmetrical chain transfer agents (CTA) to install a particular end group.^6^ However, the synthesis of different CTAs for each different end group can be very laborious. Simultaneously, the intolerance of Ru metal carbene complexes for thiol, makes a thiol end-functionalized ROMP polymer synthesis particularly challenging.^14^

Over the past few years, ROMP has been used to synthesize sequence defined polymers, mostly alternating copolymers.^15–21,66^ Several approaches have been made to achieve alternating ring-opening metathesis polymerization (AROMP) either *via* catalyst modification or through monomer design.^22–30^ In 2009, Sampson and co-workers reported an AROMP strategy where cyclobutene-1-carboxylic esters undergo ring-opening metathesis with a ruthenium catalyst giving an enoic carbene incapable of homopropagation.^31–39^

However, alternating copolymerization of cyclobutene-1-carboxylic esters with cycloalkenes lead to a sterically accessible double bond in the polymer backbone, resulting in secondary metathesis (chain transfer to the polymer). Later, Xia and co-workers introduced a creative approach for AROMP using the single molecular addition of sterically hindered cyclopropene derivatives.^40–44^ However, the preparation of cyclopropenes for AROMP is synthetically challenging.

**Fig. 1:**
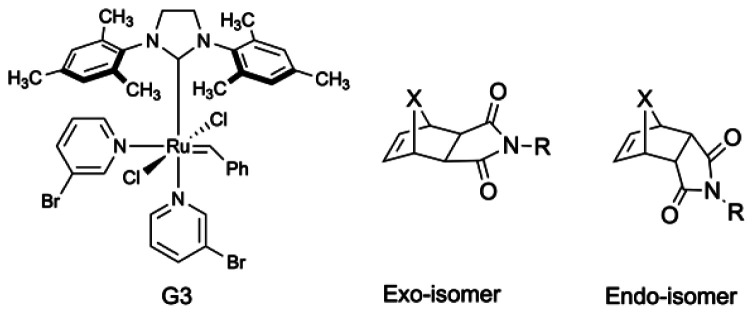
(Left) commercially available Grubbs' third generation (**G3**) catalyst. (Right) two general representations of *exo*- and *endo*- norbornene imide derivatives where X are either C (carba) or O (oxo).

Monomers like norbornene (NBE) and its derivatives (Fig. 1) are the most common choice in metathesis polymerization due to easy synthetic accessibility. Fast polymerization kinetics, easy functionalization, and absence of irreversible chain-transfer allows for a living polymerization and make NBE superior to many other monomers.^45^ However, among the two different isomers (*exo*- and *endo*-, Fig. 1) of NBE derivatives, *exo*-isomers are predominantly used.^46^ It is well-known that in the presence of a suitable metal carbene initiator, *exo*-isomers undergo rapid polymerization, whereas *endo*-isomers exhibit much slower propagation kinetics.^47–50^ The difference in reactivity of the two isomers of NBE derivatives is attributed to a higher ring strain and a more reactive propagating carbene of the *exo*-derivative.^51–56^ However, a lower ring strain and a less reactive propagating carbene of the *endo*-isomer could potentially be advantageous in controlling the polymer microstructure. Unfortunately, the detailed exploration of *endo*- norbornene derivatives towards sequential incorporation during polymerization is rare.

Several approaches have been reported for the controlled incorporation of norbornene derivatives throughout the copolymerization. The higher reactivity of NBE derivatives makes a sequential arrangement challenging. Coughlin and co-workers reported AROMP using oxanorbornene imide derivatives with Grubbs' 1st (**G1**) and 2nd (**G2**) generation catalysts.^57,58^ Recently, our group also reported AROMP with oxanorbornene imide (oxaNBE) derivatives even at elevated temperature using **G2**.^59^ However, under similar conditions, the corresponding norbornene derivatives exhibiting comparable ring strain mainly yielded block copolymers.^60–63^ To the best of our knowledge, a norbornene derivative that undergoes a strict single addition to a metathesis initiator has not yet been reported.

The slower reactivity of oxanorbornene derivatives compared to their norbornene counterparts and a more straightforward synthetic accessibility inspired us to explore such derivatives as single addition monomers. Here, we are reporting for the first time the single monomer addition of an oxanorbornene derivative to Grubbs' 3rd generation catalyst (**G3**, Fig. 1), which gives access to AROMP and a unique end functionalization method for mono-telechelic ROMP polymers.

## Results and discussion

### Initiation studies

Faster propagation kinetics of *exo*-norbornene derivatives compared to *endo*-derivatives are well established.^47–50^ However, we are not aware of detailed studies investigating the initiation kinetics of *exo*- and the corresponding *endo*-isomers.

To understand the initiation preference of **G3** (Fig. 1) for either the *exo*- or the *endo*-isomer, a control ^1^H NMR experiment was performed with a mixture of **1-exo** : **1-endo** :  **G3** (1 : 1 : 0.4) in the presence of 30 eq. of 3-bromo pyridine. 3-Bromo pyridine was used to slow down the reaction with **G3** so that it could be followed by ^1^H NMR spectroscopy. Surprisingly, preferential initiation of **G3** with **1-endo** over **1-exo** (80% *endo* carbene, 20% *exo* carbene) was clearly observed (Fig S2 and S3†). Over the course of the reaction, the concentration of the *endo* carbene increased even in the presence of **1-exo**. To generalize whether the observed preference is also valid for (7-carba) norbornene derivatives, another control ^1^H NMR experiment was performed with a 1 : 1 mixture of *endo* and *exo N*-phenylnorbornene imide in the presence of **G3** (1 : 1 : 0.5) and 30 eq. of 3-bromo pyridine. Interestingly, in this case preferential initiation with the *exo*-isomer was observed (Fig. S4†). Initially, **G3** reacted mainly with the *exo* isomer (30% *endo*-carbene, 70% *exo*-carbene) and over time (60 min) converted exclusively to the *endo* carbene after complete consumption of the *exo*-derivative.

The steric demand of both, 7-carba and 7-oxanorbornene derivatives is very similar and it is not immediately apparent to us what causes the observed difference in initiation kinetics. *Ab initio* calculations are currently being carrying out in an attempt to answer this question.

As far as the propagation rate is concerned, oxaNBEimides follow a similar trend to NBEimide derivatives. We believe this is due to the lower reactivity of the propagating *endo* carbene compared to the propagating *exo*-carbene. The lower reactivity of propagating *endo*-carbenes of NBEimide derivatives is well established in the literature.^51–56^ For geometrical reasons, the ring-opening of any *endo* norbornene or 7-oxanorbornene imide derivative results in the imide group pointing towards the Ru metal center. This results in a sterically more crowded metal coordination site and, likely, a coordination between the carbonyl group of the imide with the Ru metal center. We believe this to be the reason for the observed lower reaction rates for such carbene complexes.

In order to achieve a true single monomer addition the monomer should react only once with an initiator and should not undergo homopolymerization. The fast initiation and slow homopropagation kinetics of the *endo* oxanorbornene derivatives appeared suitable to be considered as single addition monomers. Although, **1-endo** has a low reactivity it still undergoes homopolymerization.^59^ The lower reactivity of bridgehead substituted oxanorbornene imide derivatives towards ROMP had been reported previously.^64^ The presence of steric bulk at the bridgehead position makes the monomer less accessible for coordination to alkylidene catalysts and thus results in slower polymerization.

### Slower monomers design

**Scheme 1:**
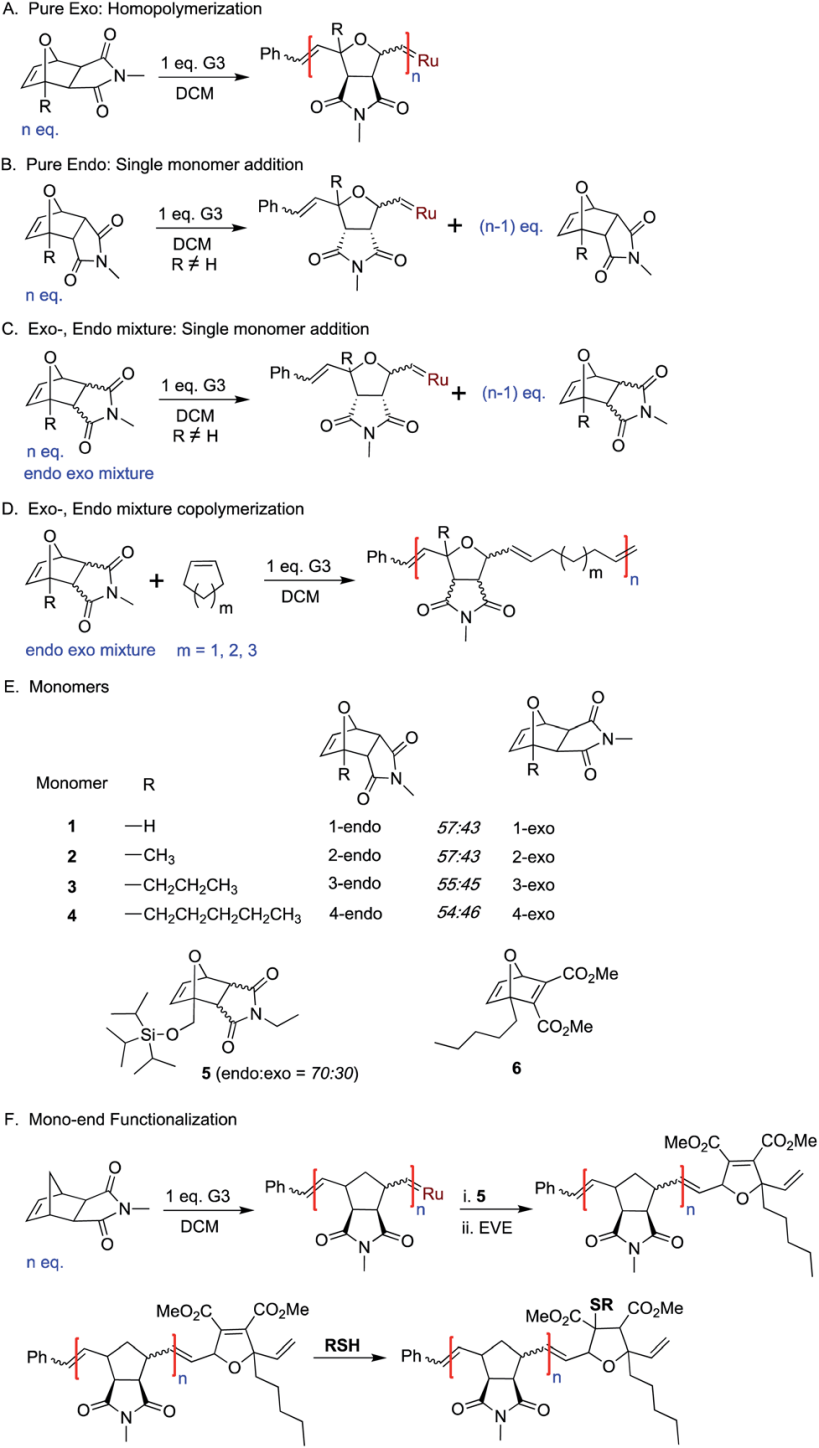
General representation of *exo* and *endo*-oxanorbornene reactivity.

Therefore, the unique reactivity of oxaNBEimide derivatives was explored more carefully by synthesizing sterically more demanding and hence less reactive bridgehead substituted derivatives of monomer **1** (monomers **2**, **3**, **4** and **5**Scheme 1E).

**Fig. 2:**
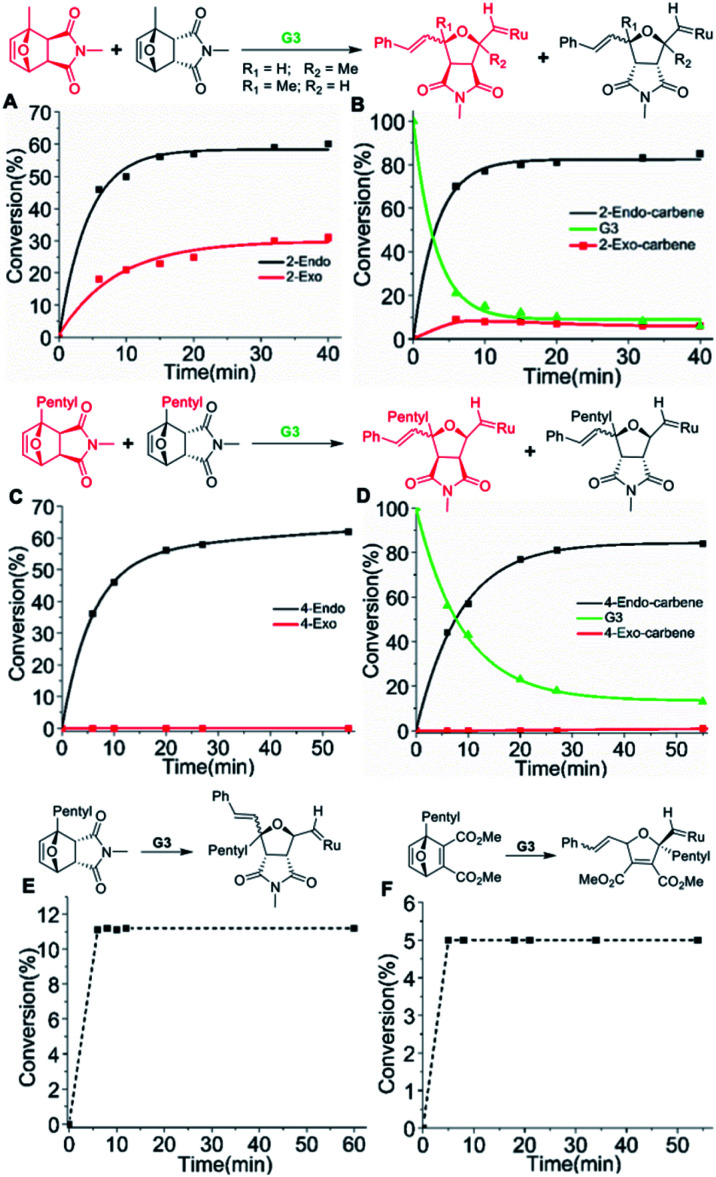
Plot of monomer and initiator conversion determined by ^1^H NMR spectra (CD_2_Cl_2_, 400 MHz) of the reactions: (A and B) mixture of **2-endo** : **2-exo** (1 : 1) with 0.4 eq. **G3**. (A) Conversion of **2-endo**, **2-exo***vs.* time showing preference for **2-endo** (B) Conversion to *endo* carbene and *exo* carbene *vs.* time showing preference for *endo* carbene. (C and D) Mixture of **4-endo** : **4-exo** (1 : 1) with 0.4 eq. **G3**. (C) Conversion of **4-endo**, **4-exo***vs.* time showing selective reaction with **4-endo** (D) conversion to *endo* carbene and *exo* carbene *vs.* time showing exclusive formation of *endo* carbene. (E) Conversion of monomer **4-endo***vs.* time showing single molecular addition of **4-endo** to **G3** (11 mol%). (F) Conversion of monomer **6***vs.* time showing single molecular addition of **6** to **G3** (5 mol%).

To investigate the initiation selectivity of **G3** and the newly synthesized monomers, control ^1^H NMR experiments were performed with mixtures of *exo*- and *endo*-monomers **2–4** and **G3** (1 : 1 : 0.4) in dichloromethane-d_2_ (Fig. 2). The lower reactivity of monomers **2–4** allows careful ^1^H NMR analysis even in the absence of 3-bromo pyridine. Interestingly, more than 94% *endo* : *exo* carbene selectivity was observed for monomer **2** (40 min, Fig. 2A, B, S5 and S6†), whereas 99.9% *endo* : *exo* selectivity was observed in the case of monomer **4** (55 min, Fig. 2C, D, S7 and S8†).

Next, the *endo* and *exo*-isomers of monomers **2** and **4** were isolated by column chromatography (see ESI†) and the homopropagation of the individual isomers was followed by ^1^H NMR spectroscopy. The effect of increasing steric bulk towards slowing down the polymerization was confirmed by homopolymerization experiments of monomers **2-exo** and **4-exo** (Scheme 1A). The ^1^H NMR analysis of the homopolymerization of monomer **2-exo** in the presence of 3 mol% of **G3**, shows complete consumption of the monomer within 5 hours whereas monomer **4-exo** was consumed only 75% in 24 hours (Fig. S9 and S10†).

Then, homopolymerizations of **2-endo**, **3-endo**, and **4-endo** with 3 mol% of **G3** were attempted. Surprisingly, no homopolymer was obtained in any of the experiments. To understand the “inertness” of the *endo* monomers towards homopolymerization, a control ^1^H NMR experiment was performed with **4-endo** and 10 mol% **G3** in dichloromethane-d2 (Scheme 1B, Fig. 2E and S11†). Surprisingly, only a single monomer addition to **G3** was observed immediately and the ^1^H-NMR spectrum remained unchanged even after 13 hours. A similar observation was also made when **2-endo** was reacted with 10 mol% of **G3** (Scheme 1B, Fig S12†).

Thereafter, homopolymerization studies were performed with mixtures of the corresponding *exo* and *endo* isomers of monomer **2** and **4** using 6.6 mol% of **G3** (Scheme 1C, Fig S13 and S14†). Even in these cases, only a single addition of the *endo*-monomer but no homopolymerization of the *exo* isomer was observed (Fig S13 and S14†).

### Copolymerizations

This clearly shows that **G3** reacts fast and selectively with the *endo*-isomers of monomers **2**, **3** and **4** and that the resulting carbene complexes cannot further propagate neither with the *endo* nor the *exo* isomer due to increased steric bulk. Consequently, in the absence of homopolymerization of the *exo*-, *endo*- mixture of monomers **2–4**, the determination of propagation preference of **G3** becomes challenging. However, a bulky monomer that does not homopolymerize could still undergo copolymerization with sterically less bulky comonomers. A set of monomers that undergo single monomer addition but do not homopolymerize is most useful for sequence-controlled polymer synthesis. One of the simplest examples for sequence control is the alternating copolymerization. If two monomers cannot homopolymerize individually but form a copolymer when mixed together, the resulting copolymer must necessarily be a strictly alternating copolymer.

Next, the potential of monomers **2–4** towards alternating copolymerization was studied. Alternating copolymerization of monomers **2–4** will allow the determination of the propagation preference of **G3** among the *exo*, *endo* isomers. Exhibiting little or no ring strain, small cycloalkenes such as cyclohexene (**Chex**), cyclopentene (**Cpen**), or cycloheptene (**Chep**) are popular comonomers in alternating ROMP polymer synthesis.^31,32,40,41^ We hypothesized that, after fast initiation of **G3** with a bulky strained monomer (such as **2–4**, see above), the formed alkylidene might still react with a less bulky cycloalkene monomer. This would lead to a sterically less hindered alkylidene which could react once more with a new strained but bulky monomer leading, eventually, to an alternating sequence.

**Fig. 3:**
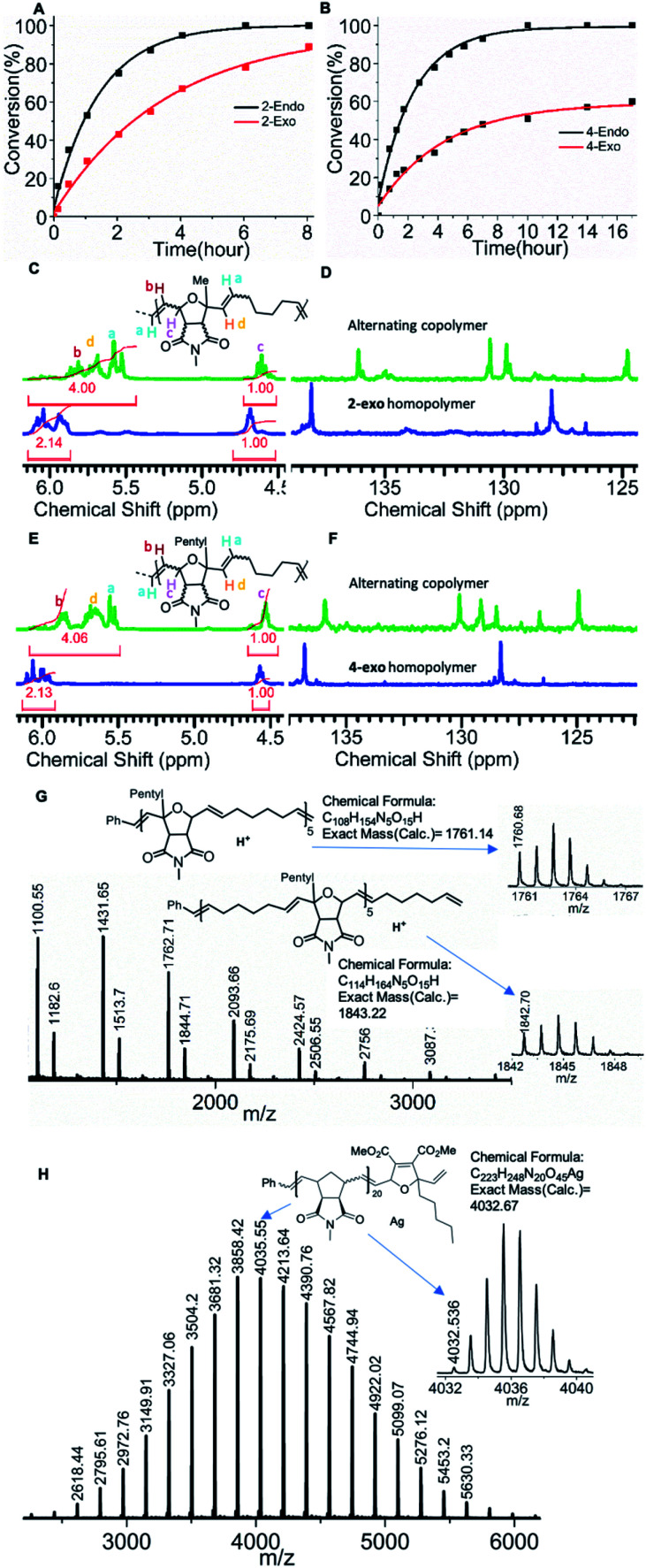
(A) Plot of **2-endo**, **2-exo** (57 : 43, 16 eq.) conversion *vs.* time in the copolymerization with cyclohexene (320 eq.) and 6.6 mol% **G3** (1 eq.). (B) Plot of **4-exo**, **4-endo** (54 : 46, 16 eq.) conversion in the copolymerization with cyclohexene (320 eq.) and 6.6 mol% **G3** (1 eq.). (C and D) Stacking of ^1^H and ^13^C NMR spectrum of the alternating copolymer synthesized from *endo*/*exo* mixture (**2** + cyclohexene) and homopolymer of **2-exo**. (E and F) Stacking of ^1^H and ^13^C NMR spectrum of the alternating copolymer synthesized from *endo*/*exo* mixture (**4** + cyclohexene) and homopolymer of **2-exo**. (G) MALDI-ToF mass spectrum of alternating copolymer synthesized from **4-endo** and cyclohexene (**P14**). (H) MALDI-ToF mass spectrum of single end capped polymer **P18**.

To verify our hypothesis, a control ^1^H NMR copolymerization experiment was performed using an *endo*/*exo*-mixture (57 : 43) of monomer **2** (16 eq.) cyclohexene (**Chex**) (320 eq.) and 6.6 mol% of **G3** (1 eq.) in the presence of an internal standard (1,3,5-trimethoxybenzene) in dichloromethane-d_2_ (Scheme 1D). Interestingly, under these conditions the consumption of both, *endo* (**2-endo**) and *exo* monomer (**2-exo**), was observed over the course of the reaction. A faster consumption of the *endo* isomer was clearly observed, which confirmed our earlier observations (see above and Fig. 3A). The fact that the *exo* monomer (**2-exo**) is also consumed over time shows that the ruthenium alkylidene complex formed after ring opening of cyclohexene is less selective than the **G3** benzylidene complex: the sterically bulky and less reactive metal carbene complex of **2-endo** cannot homopolymerize. After reaction with cyclohexene (**Chex**), an alkylidene is formed that is sterically less hindered and more reactive.^65^ This carbene is less selective than the initial benzylidene complex. It still results in a faster *endo* monomer (**2-endo**) consumption but as the concentration of the *endo*-monomer (**2-endo**) decreases, so does its rate of reaction, and thus the consumption of the *exo*-monomer (**2-exo**) increases over time.

Next, the degree of alternation in the polymer backbone was analyzed by NMR spectroscopy. As the *endo*/*exo* monomer mixture of **2** and cyclohexene (**Chex**) cannot undergo homopolymerization, a high degree of alternation was expected. A comparison of ^1^H and ^13^C NMR signals of the resulting copolymer **P1** with homopolymer of **2-exo** (Fig. 3C, D, see ESI†) shows a characteristic shift in the olefinic signals, which confirms an excellent alternating nature of the copolymer (Fig. 3C, D, see ESI,† 90% alternating diads).^57,59^ The molecular mass of the polymer **P1** obtained by size exclusion chromatography (SEC, THF, *M*_n_ = 4.1 kDa.; *Đ* = 1.2, Table 1) was close to the molecular mass determined by the [**2**] : [**G3**] ratio. Another polymerization using monomer **2** (*endo*/*exo*-mixture 57 : 43, 33 eq.) cyclohexene (660 eq.) and 3 mol% **G3** (1 eq.) targeting a higher molecular weight confirmed good molecular weight control (SEC, THF, **P2**, *M*_n_ = 10.6 kDa.; *Đ* = 1.5, Table 1).

**Table 1:** Copolymers and SEC data

Polymer	Monomers	Monomer/**G3**	*M*_n(theo)_	*M*_n(GPC)_	*Đ* a
**P1**	**2** : **Chex**	16	4.4	4.1	1.2
**P2**	**2** : **Chex**	33	9	10.6	1.5
**P3**	**2** : **Chep**	16	4.6	4.2	1.2
**P4**	**2** : **Chep**	33	9.5	10.9	1.6
**P5**	**2** : **Cpen**	16	4.2	3.9	1.2
**P6**	**3** : **Chex**	16	4.8	5.3	1.2
**P7**	**3** : **Chex**	60	18	13	1.3^*b*^
**P8**	**3** : **Cpen**	16	4.6	3.7	1.2
**P9**	**3** : **Chep**	16	5.1	5	1.2
**P10**	**4** : **Chex**	16	5.3	5.6	1.1
**P11**	**4** : **Chex**	60	19.8	14.1	1.4^*b*^
**P12**	**4** : **Cpen**	16	5.1	4.4	1.2
**P13**	**4** : **Chep**	16	5.5	5	1.2
**P14**	**4-endo** : **Chex**	16	5.3	5.8	1.1
**P15**	**5** : **Chex**	16	7.3	10.6	1.3^*b*^
**P16**	**5** : **Chex**	30	13.8	17.8	1.3^*b*^
**P17**	**5** : **Chex**	60	27.6	24.9	1.3^*b*^

a All copolymers were analysed by GPC (THF) except (*b*) which were measured in CHCl_3_.

Thereafter, copolymerizations with either cycloheptene or cyclopentene were explored with the *endo*/*exo* mixture (57 : 43) of monomer **2**. Copolymerization of monomer **2** with an equivalent amount of cycloheptene (**Chep**) with 6.6 and 3 mol% **G3** yielded polymers **P3** (SEC, THF, *M*_n_ = 4.2 kDa.; *Đ* = 1.2) and **P4** (SEC, THF, *M*_n_ = 10.9 kDa.; *Đ* = 1.6). Monomer **2** was also copolymerized with cyclopentene (**Cpen**) in the presence of 6.6 mol% **G3** to obtain polymer **P5** (SEC, THF, *M*_n_ = 3.9 kDa.; *Đ* = 1.2). The good control over molecular weight and excellent degree of alternation of all copolymers obtained was confirmed by SEC and NMR spectroscopy (see ESI, Table 1,† 92% alternating diads for **P3–P5**).

The copolymerizations of *endo*/*exo* mixture (55 : 45) of monomer **3** with cyclohexene (**Chex**), cyclopentene (**Chep**), and cycloheptene (**Chep**) were also carried out. Monomer **3** was copolymerized with cyclohexene in the presence of 6.6 mol% and 1.6 mol% **G3** to yield polymer **P6** (SEC, THF, *M*_n_ = 5.3 kDa.; *Đ* = 1.2) and **P7** (SEC, CHCl_3_, *M*_n_ = 13 kDa.; *Đ* = 1.3). Then, monomer **3** was polymerized with an equivalent amount of cyclopentene and 6.6 mol% of **G3** to obtain polymer **P8** (SEC, THF, *M*_n_ = 3.7 kDa.; *Đ* = 1.2). Polymer **P9** (SEC, THF, *M*_n_ = 5 kDa.; *Đ* = 1.2) was obtained by polymerizing monomer **3** with cycloheptene in the presence of 6.6 mol% **G3**(see ESI, Table 1).

Another control ^1^H NMR copolymerization experiment was performed using an *endo*/*exo* mixture (54 : 46) of monomer **4** (16 eq.) cyclohexene (320 eq.) and 6.6 mol% of **G3** in the presence of an internal standard (1,3,5-trimethoxybenzene) in dichloromethane-d_2_.

Similar to the copolymerization of monomer **2**, faster *endo* conversion was observed, which supports our previous observation (Fig. 3B). The polymer **P10** (SEC, THF, *M*_n_ = 5.6 kDa.; *Đ* = 1.1) obtained by this copolymerization was analyzed by SEC and NMR spectroscopy (Fig. 3E, F, see ESI, Table 1, 96% alternating diads). A high molecular weight copolymer **P11** (SEC, CHCl_3_, *M*_n_ = 14.1 kDa.; *Đ* = 1.4) was also synthesized with monomer **4** (60 eq.), cyclohexene (1200 eq.) and 1.6 mol% **G3** (1 eq.). Good control over the molecular weight even at a higher monomer to initiator ratio and the high degree of alternation of the synthesized copolymers confirmed the versatility of the copolymerization.

Copolymerization of *endo*/*exo* mixture (54 : 46) of monomer **4** with an equivalent amount of cyclopentene and 6.6 mol% **G3** yielded polymer **P12** (SEC, THF, *M*_n_ = 4.4 kDa.; *Đ* = 1.2, 96% altering diads). The *endo*/*exo* mixture (54 : 46) of monomer **4** was also copolymerized with cycloheptene in the presence of 6.6 mol% **G3** to obtain polymer **P13** (SEC, THF, *M*_n_ = 5 kDa.; *Đ* = 1.2, 94% alternating diads). The SEC and NMR analysis confirmed good molecular weight control as well as an excellent degree of alternation of the resulting polymers (see ESI, Table 1).

A copolymerization was also performed with pure **4-endo** (15 eq.) and cyclohexene (300 eq.) in the presence of 6.6 mol% **G3** (1 eq.) to obtain polymer **P14** (SEC, THF, *M*_n_ = 5.8 kDa.; *Đ* = 1.1). Neither of the two monomers has the possibility to form a homopolymer, yet a polymer (**P14**) was formed from the mixture. This by itself could be regarded as proof for strict alternation, however, the ^1^H, ^13^C NMR, and MALDI-ToF analysis confirmed the strictly alternating character of the polymer (Fig. 3G, S65, S91–92, S117–118). In particular, the MALDI-ToF mass spectrum shows a pattern in which two peaks (the higher intensity one at lower mass and the lower intensity one at higher mass) repeat. The higher intensity peak can be assigned to polymers with equal numbers of **4-endo** and cyclohexene units, whereas the lower intensity peak is formed by polymers containing one unit of cyclohexene more than **4-endo**. Other combinations of these two monomers could not be assigned which is a strong indication for a strictly alternating polymerization.

Next, a bridgehead functional monomer **5** (Scheme 1E) was tested under alternating copolymerization conditions. A control ^1^H NMR copolymerization experiment was performed with the *endo*/*exo* mixture of monomer **5** (16 eq.), cyclohexene (300 eq.) and 6.6 mol% **G3** (1 eq.) in the presence of an internal standard (1,3,5-trimethoxybenzene) in dichloromethane-d_2_. Similar to monomers **2–4**, faster consumption of **5-endo** was observed over **5-exo** during the course of the copolymerization (Fig S18,† S19). Slower copolymerization kinetics of monomer **5** are attributed to the increased steric bulk at the bridgehead position. The obtained polymer **P15** (SEC, CHCl_3_, *M*_n_ = 10.6 kDa.; *Đ* = 1.3) was analyzed by SEC and NMR, which confirmed good control over the molecular weight and a high degree of alternation of the copolymer. Two high molecular weight copolymers **P16** and **P17** were also synthesized with the *endo*/*exo* mixture of monomer **5**, cyclohexene and **G3** (3.3 and 1.6 mol%). The SEC analysis of **P16** (SEC, CHCl_3_, *M*_n_ = 17.8 kDa.; *Đ* = 1.3) and **P17** (SEC, CHCl_3_, *M*_n_ = 24.9 kDa.; *Đ* = 1.3) confirmed good control over the molecular weight (see ESI†). Furthermore, NMR analysis confirmed a high degree of alternation of the resulting copolymers (see ESI†) (Table 1).

### End-functionalization

Furthermore, we studied the use of single monomer addition towards the end functionalization of ROMP polymers. A monomer that undergoes single molecular addition while containing a highly reactive group could be very useful for the installation of functional end groups *via* post-polymerization reactions. The wide commercial availability of functional thiol derivatives and the efficient thiol-ene click reaction prompted us to design monomer **6**. We assumed that the planar geometry of the carbonyl groups in the ring-opened form of this 7-oxanorbornadiene derivative would be efficient for chelation with the metal carbene and lead to a single monomer addition.

To prove our hypothesis, a control ^1^H NMR experiment was performed with **6** and 10 mol% **G3** in dichloromethane-d_2_ (Fig. 2F, S22†). As proposed, a single monomer addition of **6** to **G3** was observed immediately and the ^1^H-NMR spectrum remained unchanged even after 9 hours.

Next, a monomer **6** terminated ROMP polymer was synthesized. *Exo N*-methylnorbornene imide (MNI, 10 eq.) was polymerized for 10 min with 3.3 mol% of **G3** followed by the addition of monomer **6** (Scheme 1F). The polymerization was quenched with an excess of ethyl vinyl ether 10 min after monomer **6** addition (see ESI†) to obtain **P18** (SEC, CHCl_3_, *M*_n_ = 6.1 kDa.; *Đ* = 1.08). A sample was collected after addition of each reagent and analysed by ^1^H NMR spectroscopy confirming complete carbene conversion (Fig S23†). ^1^H NMR and MALDI-ToF MS spectroscopy confirmed the precise single molecular addition of monomer **6** at the polymer (**P18**) chain end (Fig. 3H, S66, S103†).

The presence of an activated maleic ester in monomer **6** makes it a very good reaction partner for thiol ene click derivatization (Scheme 1F). Hence, different end functional polymers can be achieved from one single parent polymer simply by using commercial functional thiols.

To prove our hypothesis, two samples of polymer **P18** were treated separately with 10 eq. of thioethanol (**P19**) or pentanedithiol (**P20**) (Scheme 1F, see ESI†). The ^1^H NMR and MALDI-ToF analysis of both polymers confirmed the presence of polymers with either an alcohol or thiol end group with a high degree of end functionalization (>98% for the alcohol end group by ^1^H-NMR spectroscopy, Fig S67, S68, S105;† unfortunately, the percentage of thiol end groups could not be determined by ^1^H-NMR spectroscopy.).

## Conclusions

In conclusion, we have successfully developed an oxanorbornene type single addition monomer. We observed that in 7-oxanorbornene derivatives the *endo* isomer initiates faster with **G3** than the *exo* isomer. Together with a slow propagation reaction of the *endo*-isomer, this allowed us to use *endo* 7-oxanorbornenes as single addition monomers. The observed selective initiation of **G3** with the *endo* isomer prompted us to synthesize a set of bridgehead substituted 7-oxanorbornene monomers where no homopolymerization could be observed at all. The absence of homopolymerization and successful copolymerization with cycloalkenes allowed us to prepare alternating copolymers. The synthesis of a strictly alternating copolymer with **4-endo** and cyclohexene confirmed the single molecular addition in the presence of **G3**. End functionalization of ROMP polymers with reactive single addition monomer **6** allowed us to prepare a polymer carrying a reactive end group for post-polymerization functionalization. Two different end functional polymers were obtained from a single parent polymer with commercial thiol derivatives, one yielding an alcohol and the other a thiol end group. We believe that the detailed understanding of the reactivity of oxaNBE derivatives combined with their straightforward synthesis will allow easy access to sequence-controlled and end functional ROMP polymers. These polymers could be exploited as a building block for the preparation of well-defined architectures, new functional materials, and in biomedical applications.

## Author contributions

P. K. synthesized the *exo* and *endo N*-phenylnorborneneimides, M. A. carried out several polymerizations and helped in editing the manuscript, S. P. synthesized all remaining monomers, polymers and carried out all analyses. S. P. and A. F. M. K. prepared the manuscript.

## Conflicts of interest

There are no conflicts to declare.

## Supplementary Material

SC-012-D1SC00036E-s001
