# Supplementary material for: Oxanorbornenes: promising new single addition monomers for the metathesis polymerization
Source: Chem Sci. 2021 Apr 7;12(19):6705–11. doi: 10.1039/d1sc00036e (PMC8133030; doi:10.1039/d1sc00036e)
Supplement: SC-012-D1SC00036E-s001 [file SC-012-D1SC00036E-s001.pdf]

Electronic Supplementary Material (ESI) for Chemical Science.
This journal is © The Royal Society of Chemistry 2021

Supporting Information

Oxanorbornenes: Promising New Single Addition Monomers for the Metathesis Polymerization.

Subhajit Pal, Mahshid Alizadeh, Phally Kong and Andreas F.M. Kilbinger*

Department of Chemistry, University of Fribourg, Chemin du Musée 9, CH-1700 Fribourg, Switzerland.

Table of Contents

Materials....2
Characterization....2
Synthesis of substrates....3
Control ^{1}H NMR initiation experiments....5
Polymerization with cyclohexene....5
Polymerization with cycloheptene and cyclopentene....5
End capping of ROMP polymer with monomer **6**....6
End functionalization of **P18**....6
Table S1. Polymerization results and SEC data for different polymers....7
^{1}H NMR spectroscopic experiments....10
NMR spectra of monomers....20
GPC elugrams of polymers....31
MALDI-ToF mass spectra of polymers....40
NMR spectra of polymers....43
NMR spectra comparisons of homo and alternating copolymer....63
High-resolution mass spectrometric data....66

Materials

Grubbs' initiators **G3**, *N*-methylmaleimide, *N*-ethylmaleimide, 2-Methylfuran, 2-Pentylfuran, dimethyl acetylenedicarboxylate, Furfuryl alcohol, 3-Bromopyridine and 1,3,5-Trimethoxybenzene were purchased from Sigma-Aldrich and used without further purification. 2-Propylfuran and Triisopropylsilyl chloride was purchased from Alfa-Aesar and used without further purification. All other reagents and solvents were purchased from Acros organics or Sigma-Aldrich and used without further purification**.** Deuterated solvents (CD_2Cl_2, $CDCl_3$) were purchased from Cambridge Isotope Laboratories, Inc.

Characterization

All ^1H NMR (400 MHz) and ^{13}C NMR (100 MHz) spectra were recorded on a Bruker Avance DPX (360 MHz) FT NMR spectrometer. Chemical shifts were given in ppm relative to the residual solvent peak ($CDCl_3$: 7.26 for ^1H; $CDCl_3$: 77.16 for ^{13}C and CD_2Cl_2: 5.32 for ^1H; CD_2Cl_2: 53.88). HR MALDI FT-ICR mass spectra were measured on a Bruker FTMS 4.7T BioAPEX II in positive mode using trans-2-[3-(tert-butylphenyl)-2-methyl-2-propenylidene] malononitrile (DCTB) as matrix and silver trifluoroacetate (AgTFA), sodium trifluoroacetate (NaTFA) as counter ion source. HR-MS (ESI+) mass spectra were measured on a Bruker FTMS 4.7T BioAPEX II. Relative molecular weights and molecular weight distributions were measured by gel permeation chromatography (GPC) with tetrahydrofuran as eluent with a flow rate of 1 mL/min at room temperature. The system was calibrated with polystyrene standards in a range from 10^3 to 3×10^6 Da. The instrument is an automated Viscotek GPCmax VE-2001 with a set of two Viscotek T6000M linear columns (300 x 8 mm, 5 µm particle size). Signal detection occurred by use of a Viscotek Smartline 2600 UV detector (set to 254 nm wavelength) and a Viscotek VE 3580 RI detector (refractive index). The Chloroform GPC is an automated PSS SECcurity System (Agilent Technologies 1260 infinity ll) with a set two MZ-Gel SDplus linear columns (300 x 8 mm, 5 µm particle size). The Chloroform GPC was calibrated with polystyrene standards in a range from 10^3 to 3×10^6 Da and the samples were run at 40 °C and a flow rate of 1.0 mL/min.

Synthesis of substrates

N-methyl-7-oxanorborneneimide (1)

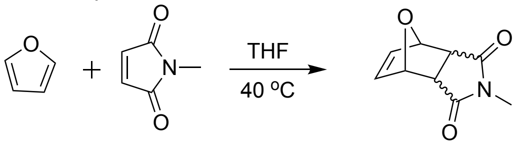

A mixture of *N*-methylmaleimide (10g, 90 mmol, 1eq) and furan (9.19g, 135 mmol, 1.5eq) was dissolved in 5 mL of tetrahydrofuran. The solution was heated to 40 ^{0}C until complete disappearance of N-methylmaleimide was observed. The reaction was concentrated under vacuum to give a white solid as 57:43 endo-, exo- mixture (15.78g, 98%). The mixture was used for metathesis without any further purifications. The mixture was also purified by column chromatography with hexane: ethyl acetate (75:25) to obtain pure **1-endo** (white solid) and **1-exo** (crystalline colorless solid). The endo isomer was stored at -20 ^{0}C. Endo- isomer: ^{1}H NMR (400 MHz, CHLOROFORM-*d*) δ 6.39 (t, *J*=0.86 Hz, 2 H), 5.32 (ddd, *J*=2.69, 1.71, 0.86 Hz, 2 H), 3.52 (dd, *J*=3.61, 1.65 Hz, 2 H), 2.81 (s, 3 H) ppm. Exo- isomer: ^{1}H NMR (400 MHz, CHLOROFORM-*d*) δ 6.51 (t, *J*=0.86 Hz, 2 H), 5.26 (t, *J*=0.86 Hz, 2 H), 2.97 (s, 3 H), 2.85 (s, 2 H) ppm. HR-MS (ESI) calcd. For $C_9H_9NO_3H$+ [M+H]+: 180.0661; Found: 180.0658.

4-Methyl-*N*-methyl-7-oxanorbornenecarboximide (2)

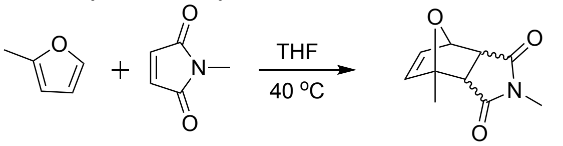

A mixture of *N*-methylmaleimide (10g, 90 mmol, 1eq) and 2-methylfuran (14.7g, 180 mmol, 2 eq) was dissolved in 5 mL of tetrahydrofuran. The solution was heated to 40 ^{0}C until complete disappearance of *N*-methylmaleimide was observed. The reaction was concentrated under vacuum to give a white solid as 57:43 endo-, exo- mixture (17g, 98%). The mixture was used for metathesis without any further purifications. The mixture was also purified by column chromatography with hexane: ethyl acetate (80:20) to obtain pure **2-endo** (white solid) and **2-exo** (crystalline colorless solid). The endo isomer was stored at -20 ^{0}C. Endo- isomer: ^{1}H NMR (400 MHz, CHLOROFORM-*d*) δ 6.38 (dd, J=5.75, 1.47 Hz, 1 H), 6.21 (d, J=5.75 Hz, 1 H), 5.21 (dd, J=5.56, 1.65 Hz, 1 H), 3.64 (dd, J=7.52, 5.56 Hz, 1 H), 3.11 (d, J=7.58 Hz, 1 H), 2.81 (s, 3 H), 1.83 (s, 3 H) ppm. Exo- isomer: ^{1}H NMR (400 MHz, CHLOROFORM-*d*) δ 6.49 (dd, J=5.62, 1.59 Hz, 1 H), 6.29 (d, J=5.62 Hz, 1 H), 5.16 (d, J=1.71 Hz, 1 H), 2.92 - 3.00 (m, 4 H), 2.70 (d, J=6.48 Hz, 1 H), 1.71 (s, 3 H)6.51 (dd, J=5.64, 1.51 Hz, 1 H), 6.31 (d, J=5.69 Hz, 1 H), 5.18 (d, J=1.74 Hz, 1 H), 2.93 - 3.01 (m, 4 H), 2.72 (d, J=6.51 Hz, 1 H), 1.73 (s, 3 H) ppm. Endo-isomer: ^{13}C NMR (101 MHz, CHLOROFORM-*d*) δ 175.2, 175.1, 137.6, 134.9, 88.4, 79, 50.8, 48.8, 24.5, 18.4 ppm. Exo-mixture: ^{13}C NMR (101 MHz, CHLOROFORM-*d*) δ 176.1, 174.9, 140.4, 136.8, 88, 80.5, 50.6, 49.4, 24.8, 15.6 ppm. MS (ESI) calcd. For $C_{10}H_{11}NO_3Na$+ [M+H]+: 216.0637; Found: 216.0295.

4-Propyl-*N*-methyl-7-oxanorbornenecarboximide (3)

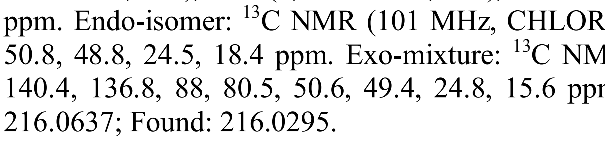

A mixture of *N*-methylmaleimide (10g, 90 mmol, 1eq) and 2-propylfuran (19.8g, 180 mmol, 2 eq) was dissolved in 5 mL of tetrahydrofuran. The solution was heated to 40 ^{0}C until complete disappearance of *N*-methylmaleimide was observed. The reaction was concentrated under vacuum to give a white solid as 57:43 endo-, exo- mixture (19.5g, 98%). The mixture was used for metathesis without any further purifications. Endo- isomer: ^{1}H NMR (300 MHz, CHLOROFORM-*d*) δ 6.35 - 6.38 (m, 1 H), 6.25 (d, J=5.69 Hz, 1 H), 5.23 (dd, J=5.55, 1.60 Hz, 1 H), 3.61 (dd, J=7.52, 5.50 Hz, 1 H), 3.16 (d, J=7.61 Hz, 1 H), 2.81 (s, 3 H), 1.88 - 2.12 (m, 2 H), 1.42 - 1.73 (m, 2 H) 1.02 (m, 3 H) ppm. Exo- isomer: ^{1}H NMR (300 MHz, CHLOROFORM-*d*) δ 6.50 (dd, J=5.73, 1.60 Hz, 1 H), 6.38 (d, J=5.69 Hz, 1 H), 5.20 (d,

J=1.74 Hz, 1 H), 2.92 - 2.99 (m, 4 H), 2.76 (d, J=6.42 Hz, 1 H), 2.16 - 2.33 (m, 2 H), 1.42 - 1.73 (m, 2 H), 1.02 (td, J=7.36, 3.44 Hz, 3 H) ppm. Endo-, Exo-mixture: ^{13}C NMR (75 MHz, CHLOROFORM-*d*) δ 176.3, 175.3, 175.2, 174.9, 138.9, 136.8, 136.7, 134.7, 91.9, 91.7, 80.4, 78.8, 50.5, 49.7, 49.1, 48.5, 34.4, 31.7, 24.8, 24.5, 18.7, 17.9, 14.5, 14.4 ppm. HR-MS (ESI) calcd. For $C_{12}H_{15}NO_3Na^+$ [M+Na]+: 244.0950 ; Found: 244.0949.

4-Pentyl-*N*-methyl-7-oxanorbornenecarboximide (4)

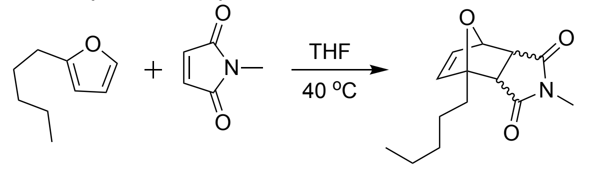

A mixture of *N*-methylmaleimide (10g, 90 mmol, 1eq) and 2-pentylfuran (24.8g, 180 mmol, 2 eq) was dissolved in 5 mL of tetrahydrofuran. The solution was heated to 40 ^{0}C until complete disappearance of *N*-methylmaleimide was observed. The reaction was concentrated under vacuum to give a white solid as 57:43 endo-, exo- mixture (21.9g, 98%). The mixture was used for metathesis without any further purifications. The mixture was also purified by column chromatography with hexane: ethyl acetate (90:10) to obtain pure **4-endo** (yellow waxy solid) and **4-exo** (crystalline colorless solid). The endo isomer was stored at -20 ^{0}C. Endo- isomer: ^{1}H NMR (400 MHz, CHLOROFORM-*d*) δ 6.35 (dd, J=5.75, 1.47 Hz, 1 H), 6.24 (d, J=5.75 Hz, 1 H), 5.22 (dd, J=5.56, 1.65 Hz, 1 H), 3.60 (dd, J=7.52, 5.56 Hz, 1 H), 3.15 (d, J=7.46 Hz, 1 H), 2.80 (s, 3 H), 2.19 - 2.33 (m, 1 H), 1.94 - 2.08 (m, 1 H), 1.44 - 1.59 (m, 2 H), 1.28 - 1.42 (m, 4 H), 0.82 - 0.95 (m, 3 H) ppm. Exo- isomer: ^{1}H NMR (400 MHz, CHLOROFORM-*d*) δ 6.49 (dd, J=5.69, 1.65 Hz, 1 H), 6.38 (d, J=5.75 Hz, 1 H), 5.19 (d, J=1.71 Hz, 1 H), 2.90 - 3.01 (m, 4 H), 2.75 (d, J=6.36 Hz, 1 H), 1.89 - 2.14 (m, 2 H), 1.54 - 1.67 (m, 1 H), 1.30 - 1.54 (m, 5 H), 0.86 - 0.95 (m, 3 H)ppm. Endo-mixture: ^{13}C NMR (101 MHz, CHLOROFORM-*d*) δ 175.3, 175.1, 136.7, 134.7, 91.9, 78.8, 49.7, 48.5, 32.2, 31.9, 24.5, 24.2, 22.4, 13.9 ppm. Exo-mixture: ^{13}C NMR (101 MHz, CHLOROFORM-*d*) δ 176.2, 174.9, 138.9, 136.8, 91.8, 80.4, 50.5, 49, 32.1, 29.5, 24.9, 24.8, 22.5, 13.9 ppm. HR-MS (ESI) calcd. For $C_{14}H_{19}NO_3Na^+$ [M+Na]+: 272.1263 ; Found: 272.1258.

4-Methoxytriisopropylsilane-*N*-methyl-7-oxanorbornenecarboximide (5)

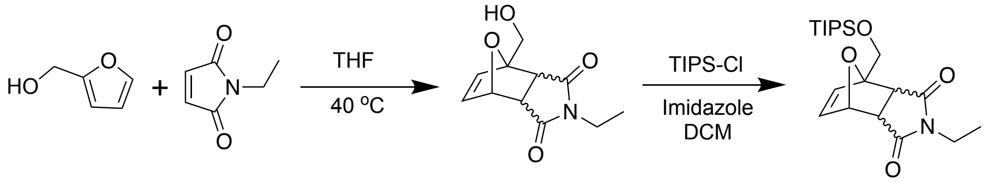

A mixture of *N*-ethylmaleimide (5g, 40 mmol, 1eq) and furfuryl alcohol (7.8g, 80 mmol, 2 eq) was dissolved in 2 mL of tetrahydrofuran. The solution was heated to 40 ^{O}C until complete disappearance of *N*-ethylmaleimide was observed. The reaction was concentrated under vacuum to give a yellow liquid as 70:30 endo-, exo- mixture (8.8g, 99%). The mixture was used for next step without any further purifications.

A mixture of crude product (4g, 17.9 mmol, 1eq) and imidazole (3.6g, 53.8 mmol, 3eq) were dissolved in 30 mL dry dichloromethane and cooled to 0 ^{O}C. To the precooled solution triisopropylsilyl chloride (4.1g, 21.5 mmol, 1.2eq) was added dropwise and the mixture was allowed to warm to room temperature. The reaction mixture was stirred for a further 12 hours at room temperature. The reaction mixture was concentrated under reduced pressure and purified by column chromatography with hexane: ethyl acetate (95:5) to obtain **5** as endo/exo mixture (6.1g, 90%). Endo- isomer: ^{1}H NMR (300 MHz, CHLOROFORM-*d*) δ 6.40 (dd, J=5.78, 1.56 Hz, 1 H), 6.26 (d, J=5.78 Hz, 1 H), 5.29 (dd, J=5.23, 1.56 Hz, 1 H), 4.27 - 4.40 (m, 2 H), 3.47 - 3.64 (m, 2 H), 3.38 (q, J=7.15 Hz, 2 H), 0.96 - 1.23 (m, 24 H) ppm. Exo- isomer: ^{1}H NMR (300 MHz, CHLOROFORM-*d*) δ 6.59 (d, J=5.69 Hz, 1 H), 6.50 (dd, J=5.73, 1.60 Hz, 1 H), 5.24 (d, J=1.65 Hz, 1 H), 4.42 (d, J=7.61 Hz, 1 H), 4.08 (d, J=11.28 Hz, 1 H), 3.48 - 3.54 (m, 2 H), 2.94 (d, J=6.42 Hz, 1 H), 2.86 (d, J=6.42 Hz, 1 H), 0.96 - 1.23 (m, 24 H) ppm. Endo-, Exo-mixture: ^{13}C NMR (75 MHz, CHLOROFORM-*d*) δ 176, 175.1, 175, 174.5, 138.4, 136.2, 134.9, 92.8, 91.8, 81, 79.5, 77.4, 76.6, 61.7, 61.1, 50.2, 47.8, 47.7, 44.8, 33.8, 33.3, 17.9, 12.9, 12.6, 11.9 ppm. MS (ESI) calcd. For $C_{20}H_{33}NO_4SiNa^+$ [M+Na]+: 402.2077; Found: 402.0018.

Dimethyl-1-pentyl-7-oxabicyclo[2.2.1]hepta-2,5-diene-2,3-dicarboxylate (6)

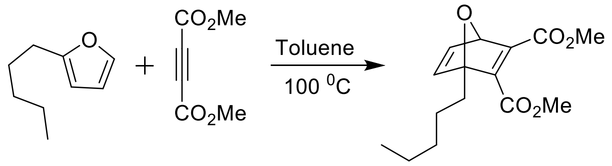

A mixture of dimethyl acetylenedicarboxylate (1g, 7 mmol, 1eq), 2-pentylfuran (1.2g, 8.4 mmol, 1.2 eq) and 3 mL of toluene was heated to 100 ^{0}C for 24h. The reaction was concentrated under reduced pressure and purified by column chromatography with hexane: ethyl acetate (90:10) to obtain monomer **5** as red liquid (1.8g, 94%). HR-MS (ESI) calcd. For $C_{15}H_{20}O_{5Na}$+ [M+Na]+: 303.1208 ; Found: 303.1198.

Control ^{1}H NMR initiation experiments

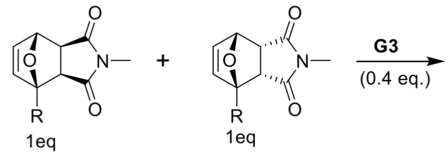

An equal mixture of endo- and exo- isomer of monomer (***1-4***) was dissolved in 0.5 mL of dry degassed dichloromethane-d2. The resulting solution was transferred to degassed NMR tube containing internal standard (1, 3, 5 trimethoxybenzene) and ^{1}H NMR spectra was measured. Separately, 0.4 eq of **G3** was dissolved in dry degassed dichloromethane-d2 and quickly added to the NMR tuhbe, ensuring efficient mixing. Then, ^{1}H NMR spectrum of the reaction mixture was recorded over time.

Polymerization with cyclohexene

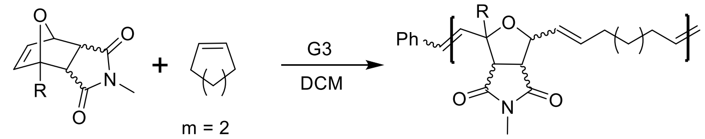

The monomer (***2-4***) and 20eq. of cyclohexene were dissolved in dry degassed dichloromethane to obtain 0.3M monomer solution (with respect to ***2-4***). Separately, a stock solution of catalyst **G3** was prepared. The required amount of **G3** solution was quickly added to the monomer solution and ensuring efficient mixing. The reaction mixture was stirred for 12-18 hours (for monomer ***2***, 12 hours and monomer ***3-4***, 18 hours) at rt under argon atmosphere. The reactions were quenched with excess ethyl vinyl ether and precipitated into cold methanol to obtain alternating polymers.

Polymerization with cycloheptene and cyclopentene

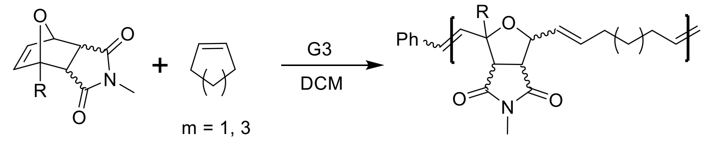

An equimolar mixture of monomers (***2-4***) and cycloheptene/ cyclopentene were transfer into vials equipped with a stir bar under argon atmosphere. Separately, a stock solution of catalyst **G3** was prepared. The required amount of **G3** solution was quickly added to the monomer solution and ensuring efficient mixing. The reaction mixture was stirred for 12-18 hours (for monomer ***2***, 12 hours and monomer ***3-4***, 18 hours) at rt under argon atmosphere. The reactions were quenched with excess ethyl vinyl ether and precipitated into cold methanol to obtain alternating polymers.

End capping of ROMP polymer with monomer 6

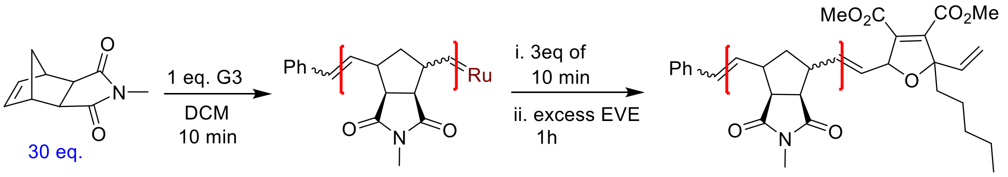

Monomer MNI (0.5g, 2.8 mmol, 30 eq) was dissolved in 2.5 mL dry degassed dichloromethane. Separately, a stock solution of catalyst **G3** was prepared. The required amount of **G3** (83 mg, 0.09 mmol, 1eq) solution was quickly added to the monomer solution and ensuring efficient mixing. The reaction mixture was stirred for 10 min and an aliquot was collected for ^{1}H NMR measurement. A stock solution of monomer **6** was prepared and required amount of monomer **6** (76 mg, 0.27 mmol, 3eq) solution was quickly added to the polymerization mixture was continue stirring for 10 min. A sample was collected for ^{1}H NMR measurement. Then, the reaction was quenched with excess of ethyl vinyl ether. Finally, the reaction mixture was concentrated under reduced pressure and precipitated into cold methanol to obtain **P18** (SEC, THF, Mn=6.1 kDa.; Đ=1.08).

End functionalization of P18

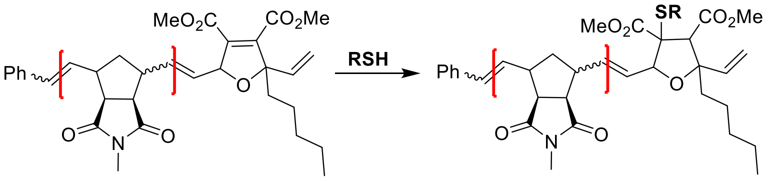

Polymer **P18** (40 mg, 1eq) was dissolved in 0.5 mL of dry THF. Separately, 10eq of thiol derivative was dissolved in 0.5 mL of dry DMF and 0.2eq of NaH (60% dispersed in mineral oil) was added. The resulting mixture was stirred for 20 min and quickly transfer into the polymer solution. The reaction mixture was heated at 40 ^{0}C for 12 h. Finally, the reaction mixture was concentrated under reduced pressure and precipitated into cold methanol to obtain polymer **P19** (ethanolthiol) or **P20** (pentanedithiol).

Table S1. Polymerization results and SEC data for different polymers

Polymer	Monomer (M)	Monomer to Initiator ratio (M/G3)	M_{n-theo} [kDa]	M_{n-GPC} [kDa]	Ð[a]	Time [Hour]	Yield [%]
**P1**	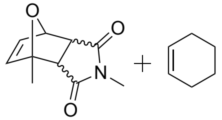 1eq. 20eq.	16	4.4	4.1	1.2	12	90
**P2**	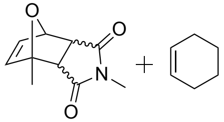 1eq. 20eq.	33	9	10.6	1.5	18	90
**P3**	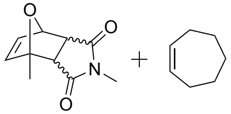 1eq. 1eq.	16	4.6	4.2	1.2	12	94
**P4**	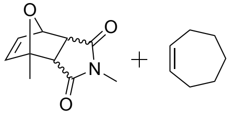 1eq. 1eq.	33	9.5	10.9	1.6	18	94
**P5**	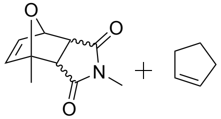 1eq. 1eq.	16	4.2	3.9	1.2	12	92
**P6**	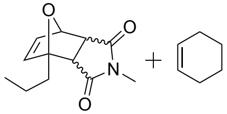 1eq. 20eq.	16	4.8	5.3	1.2	18	80

**P7**[b]	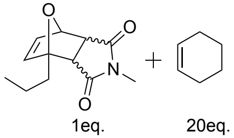	60	18	13	1.3	40	70
**P8**	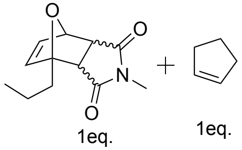	16	4.6	3.7	1.2	18	85
**P9**	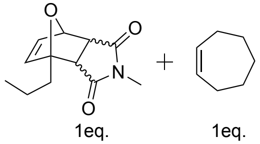	16	5.1	5	1.2	18	88
**P10**	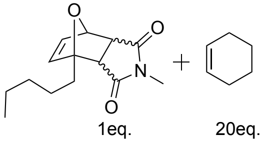	16	5.3	5.6	1.1	20	80
**P11**[b]	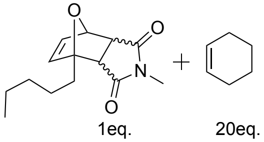	60	19.8	14.1	1.4	40	70
**P12**	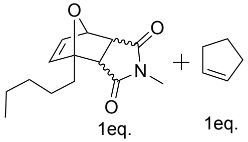	16	5.1	4.4	1.2	20	80
**P13**	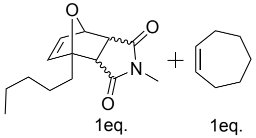	16	5.5	5	1.2	20	84
**P14**	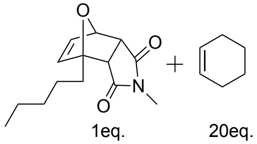	16	5.3	5.8	1.1	12	90

**P15**[b]	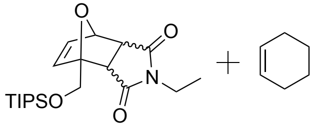	16	7.3	10.6	1.3	35	70
**P16**[b]	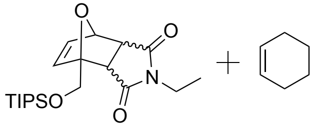	30	13.8	17.8	1.3	47	70
**P17**[b]	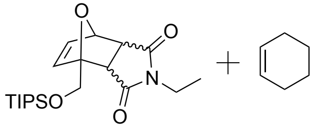	60	27.6	24.9	1.3	67	70

a. All the copolymers were analysed by THF GPC except **b** which are measured in $CHCl_3$ GPC.

^{1}H NMR spectroscopic experiments

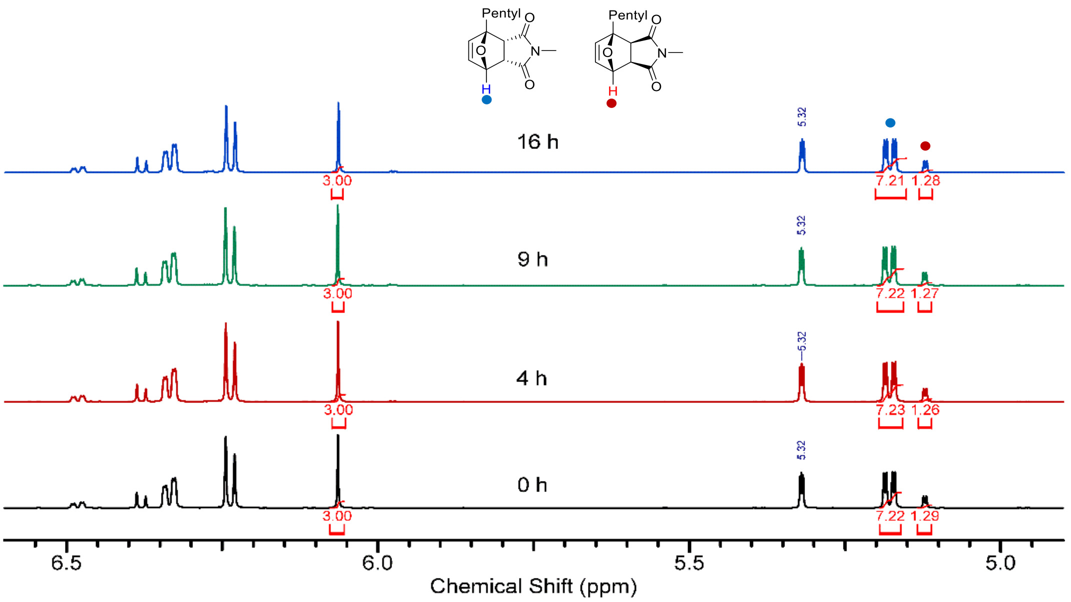

Fig S1. ^{1}H NMR (CD_2Cl_2, 400 MHz) analysis of the stability of 4-exo and 4-endo in solution. A biased concentration (85% **4-endo** and 15% **4-exo** in the presence of an internal standard 1, 3, 5 trimethoxybenzene) of *exo* and *endo* isomer was used to study dynamic behavior between two isomers. Over 16 hours in solution at rt no appreciable change was detected.

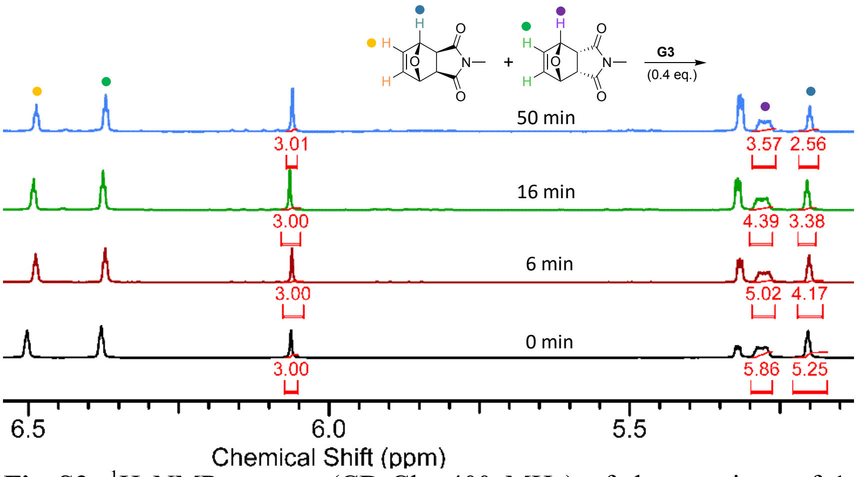

Fig S2. ^{1}H NMR spectra (CD_2Cl_2, 400 MHz) of the reactions of 1:1 mixture of **endo-**, **exo- N-methylnorborneneimide** with 0.4 eq **G3** in presence of internal standard 1, 3, 5 trimethoxybenzene and 30 eq. 3-bromopyridine.

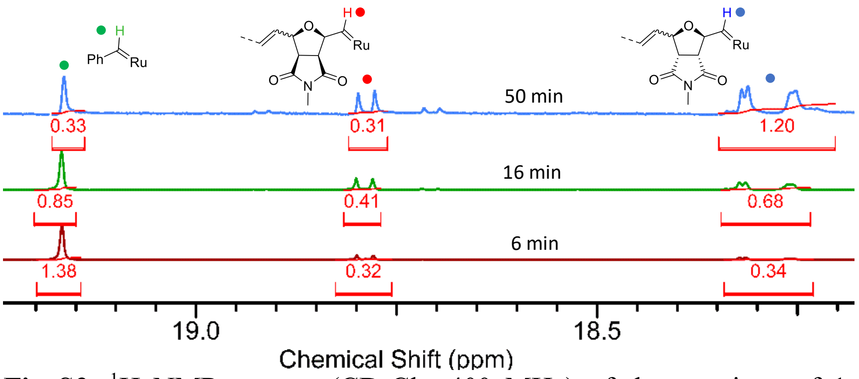

Fig S3. ^{1}H NMR spectra (CD_2Cl_2, 400 MHz) of the reactions of 1:1 mixture of **endo-**, **exo- N-methylnorborneneimide** with 0.4 eq **G3** in presence of internal standard 1, 3, 5 trimethoxy benzene and 30 eq. 3-bromopyridine.

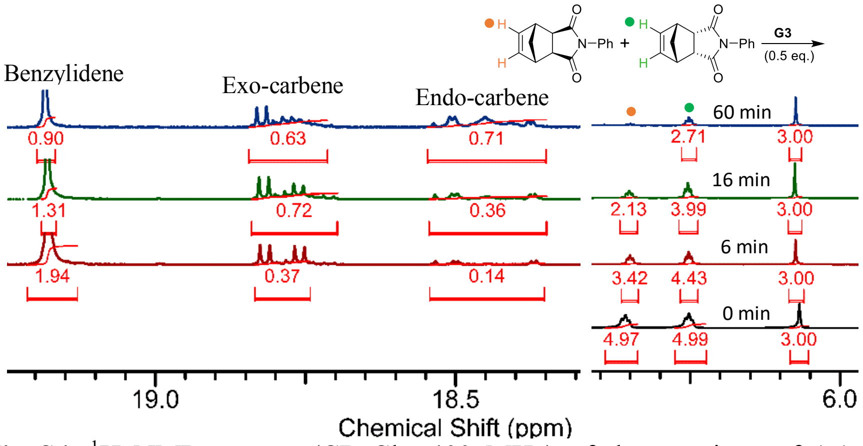

Fig S4. ^{1}H NMR spectra (CD_2Cl_2, 400 MHz) of the reactions of 1:1 mixture of **endo-**, **exo- N-phenylnorborneneimide** with 0.5 eq **G3** in presence of internal standard 1, 3, 5 trimethoxy benzene and 30 eq. 3-bromopyridine.

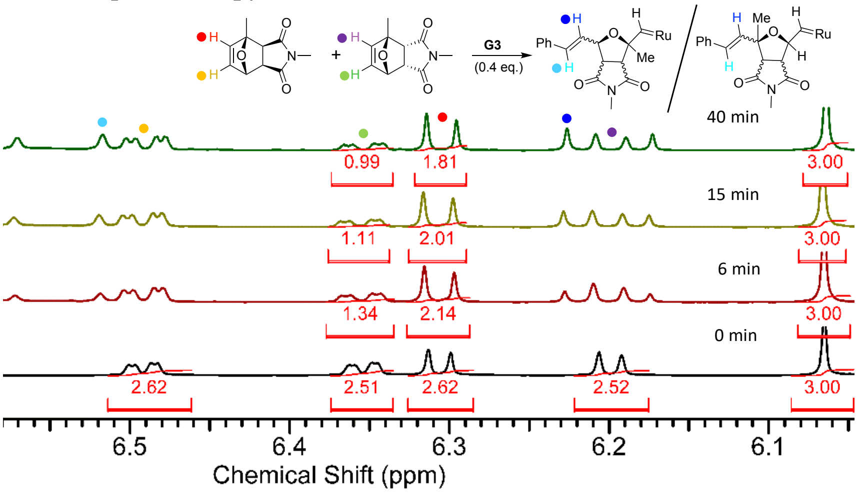

Fig S5. ^{1}H NMR spectra (CD_2Cl_2, 400 MHz) of the reactions of 1:1 mixture of **2-endo**, **2-exo** with 0.4 eq **G3** in presence of internal standard 1, 3, 5 trimethoxybenzene (Monomer double bond are focused).

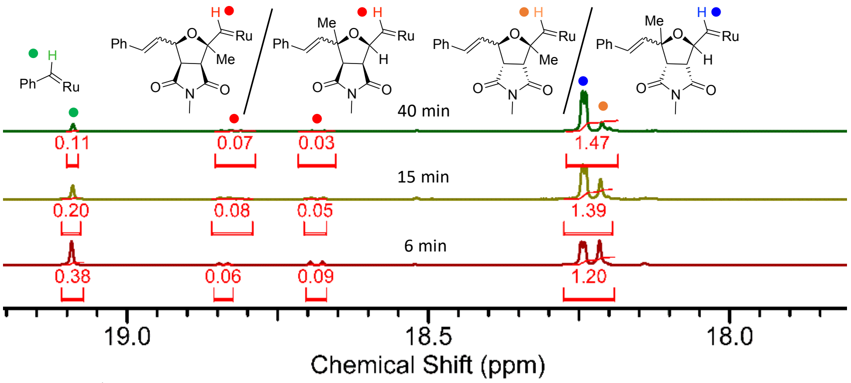

Fig S6. ^{1}H NMR spectra (CD_2Cl_2, 400 MHz) of the reactions of 1:1 mixture of **2-endo-**, **2-exo** with 0.4 eq **G3** in presence of internal standard 1, 3, 5 trimethoxybenzene (Metal carbene signal are focused).

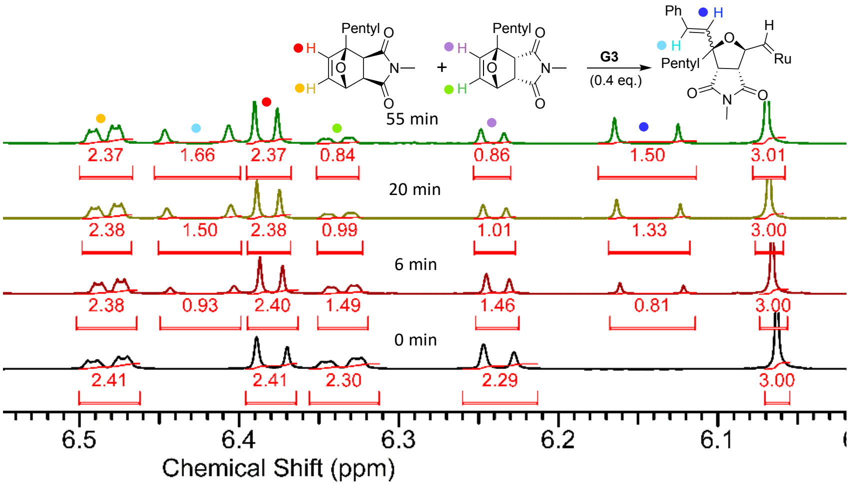

Fig S7. ^{1}H NMR spectra (CD_2Cl_2, 400 MHz) of the reactions of 1:1 mixture of **4-endo**, **4-exo** with 0.4 eq **G3** in presence of internal standard 1, 3, 5 trimethoxybenzene (Monomer double bond are focused).

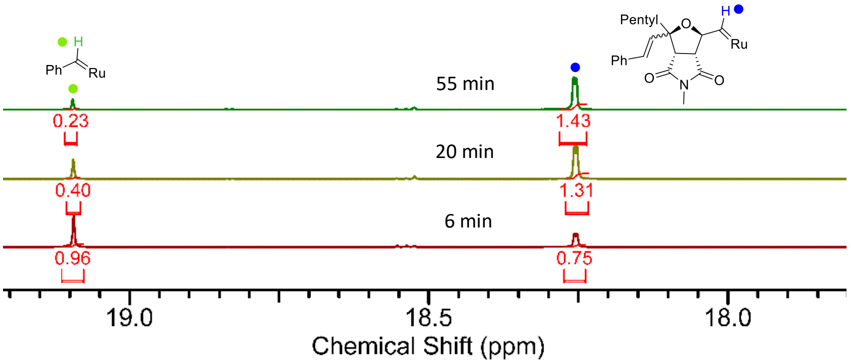

Fig S8. ^{1}H NMR spectra (CD_2Cl_2, 400 MHz) of the reactions of 1:1 mixture of **4-endo-**, **4-exo** with 0.4 eq **G3** in presence of internal standard 1, 3, 5 trimethoxybenzene (Metal carbene signal are focused).

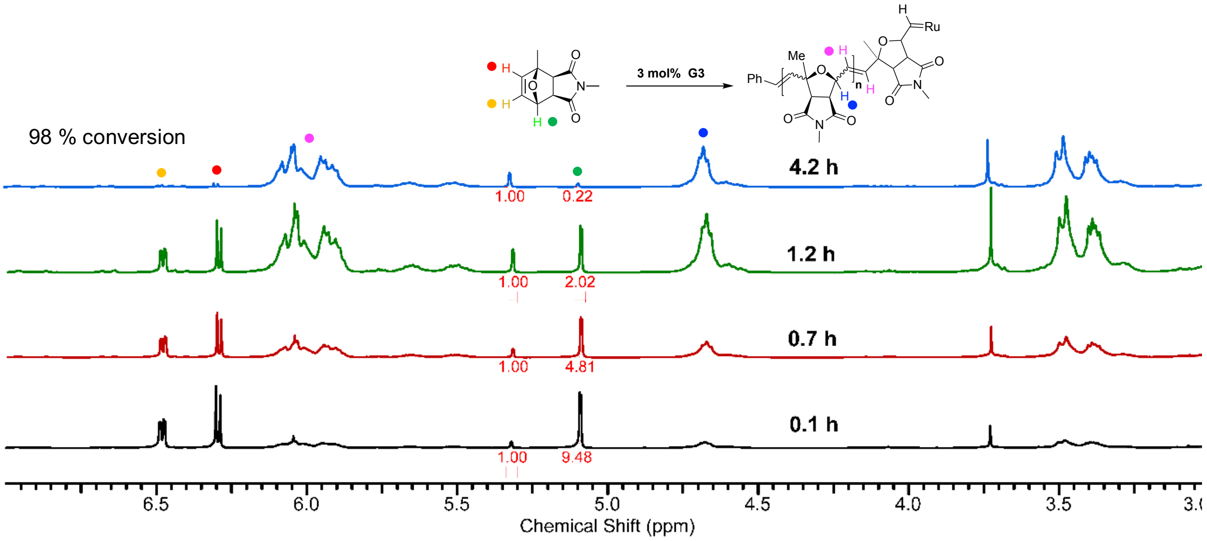

Fig S9. ^{1}H NMR spectra (CD_2Cl_2, 400 MHz) of the reactions of **2-exo** with 3 mol% **G3** in presence of internal standard 1, 3, 5 trimethoxy benzene.

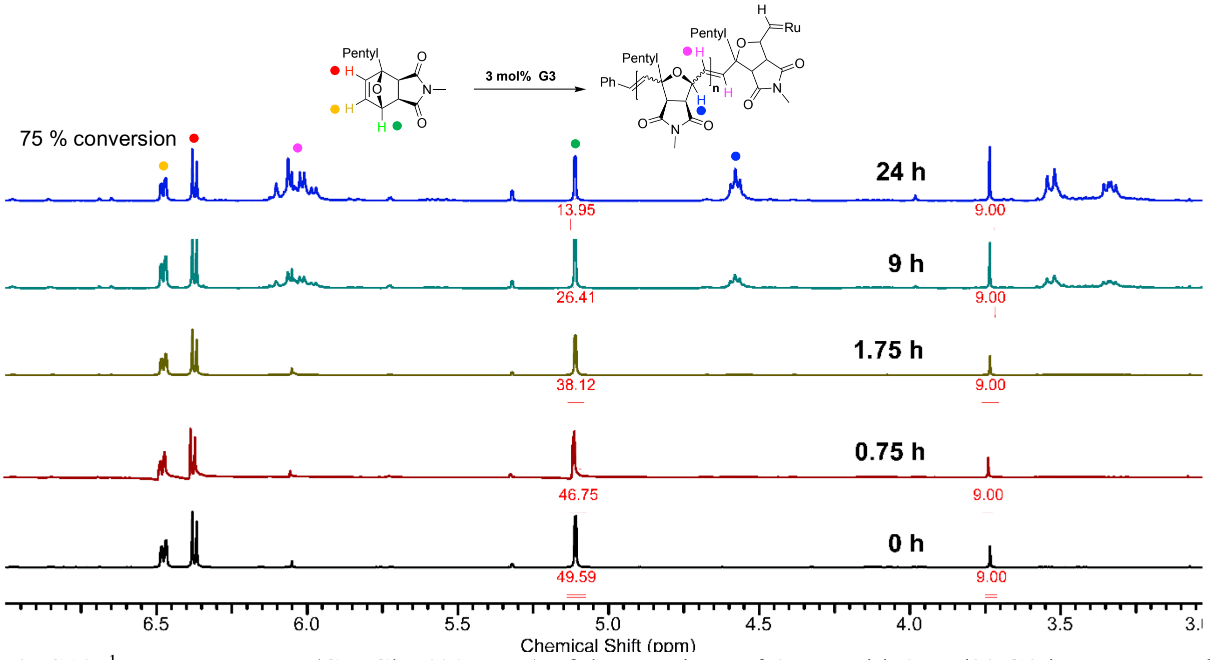

Fig S10. ^{1}H NMR spectra (CD_2Cl_2, 400 MHz) of the reactions of **4-exo** with 3 mol% **G3** in presence of internal standard 1, 3, 5 trimethoxy benzene.

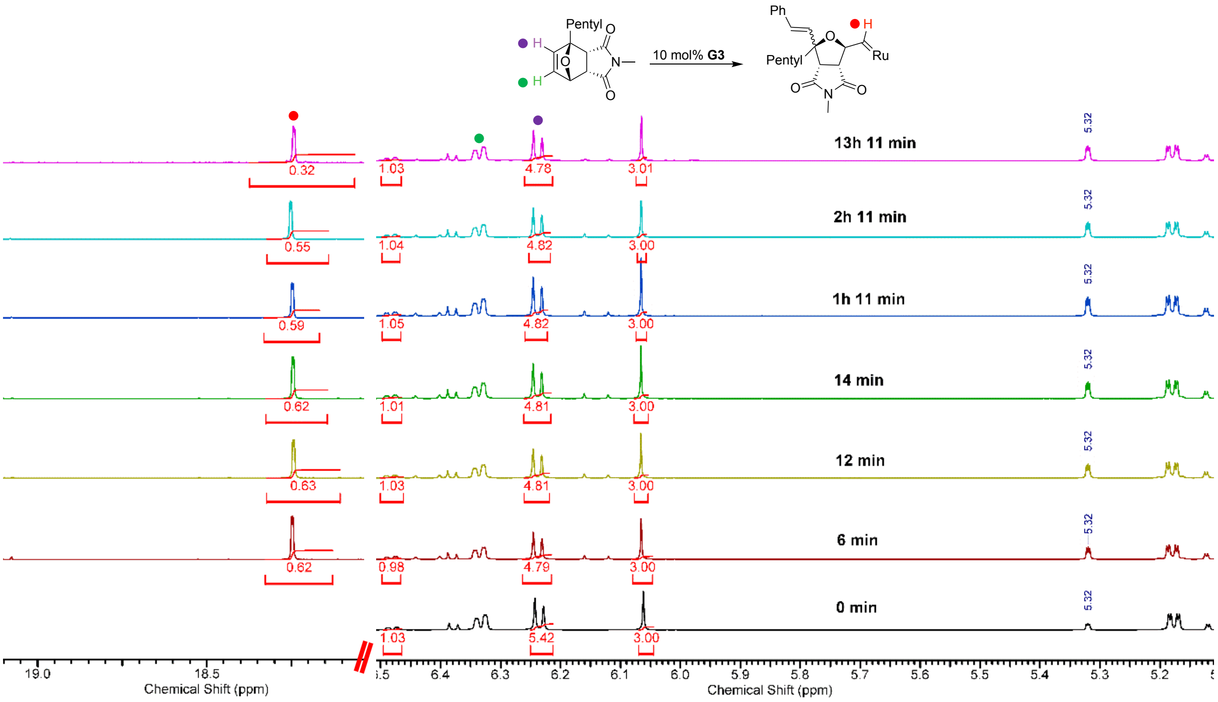

Fig S11. ^{1}H NMR spectra (CD_2Cl_2, 400 MHz) of the reactions of **4-endo** with 10 mol% **G3** in presence of internal standard 1, 3, 5 trimethoxy benzene. 17% **4-exo** impurity was present (integration 1.04, δ 6.48 ppm).

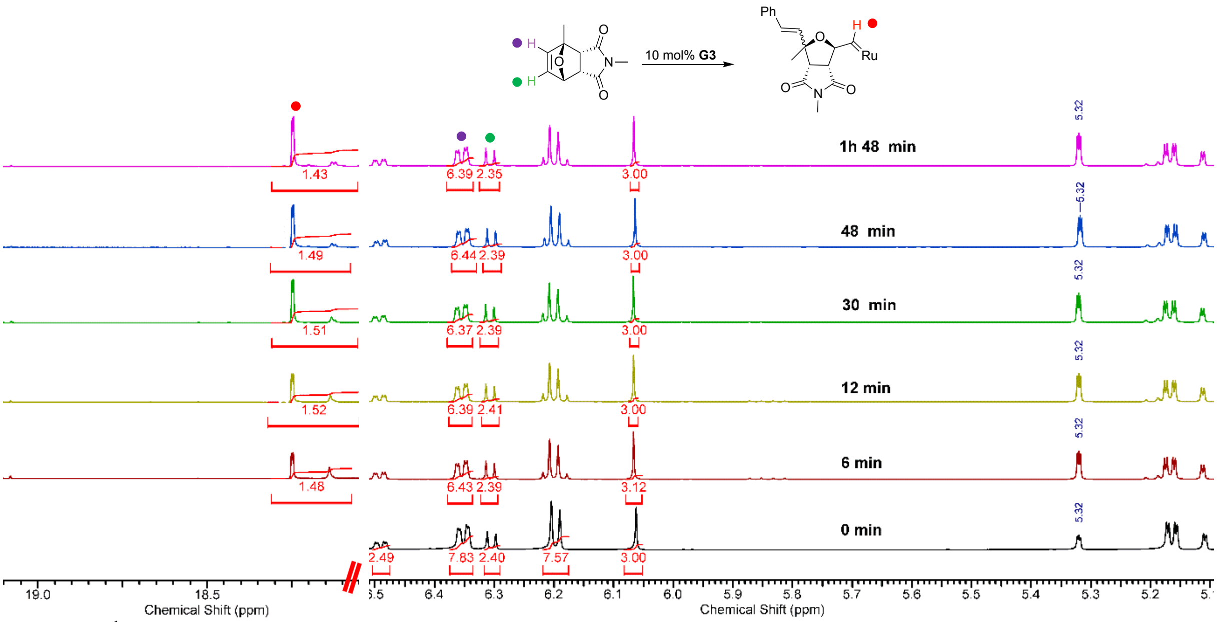

Fig S12. ^{1}H NMR spectra (CD_2Cl_2, 400 MHz) of the reactions of **2-endo** with 10 mol% **G3** in presence of internal standard 1, 3, 5 trimethoxy benzene. 24% **4-exo** impurity was present (integration 2.40, δ 6.3 ppm).

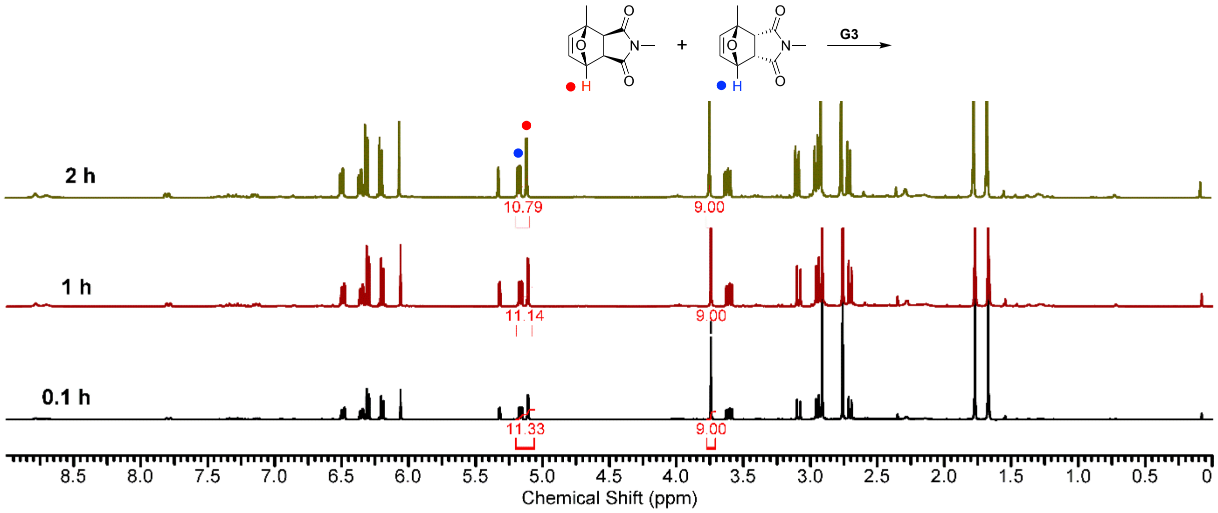

Fig S13. ^{1}H NMR spectra (CD_2Cl_2, 300MHz) of the homopolymerization of **2** (Endo-, Exo- mixture) with 6.6 mol% **G3** in the presence of the internal standard 1, 3, 5-trimethoxybenzene.

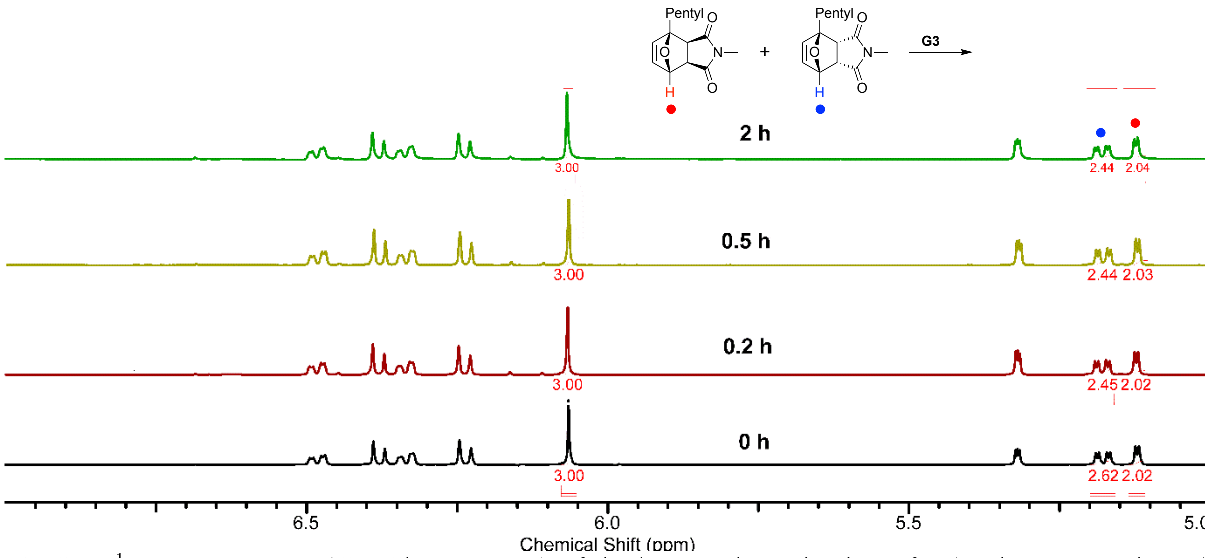

Fig S14. ^{1}H NMR spectra (CD_2Cl_2, 300MHz) of the homopolymerization of **4** (Endo-, Exo- mixture) with 6.6 mol% **G3** in the presence of the internal standard 1, 3, 5-trimethoxybenzene.

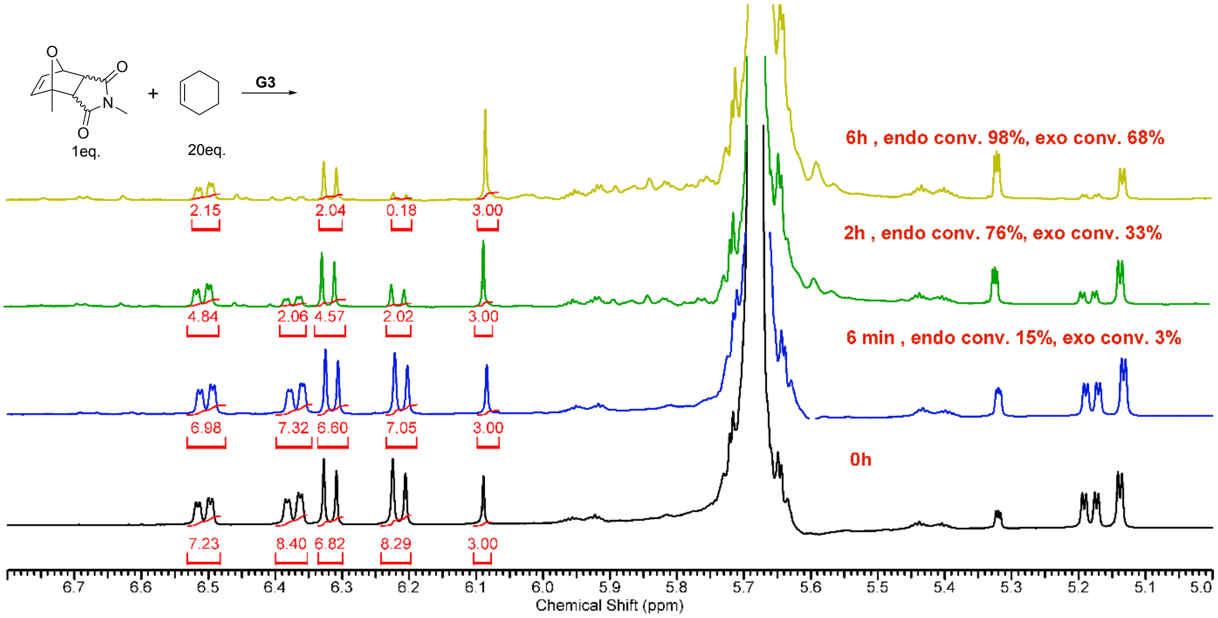

Fig S15. ^{1}H NMR spectra (CD_2Cl_2, 300MHz) of the copolymerization of **2** and cyclohexene with 6.6 mol% **G3** in the presence of the internal standard 1, 3, 5-trimethoxybenzene.

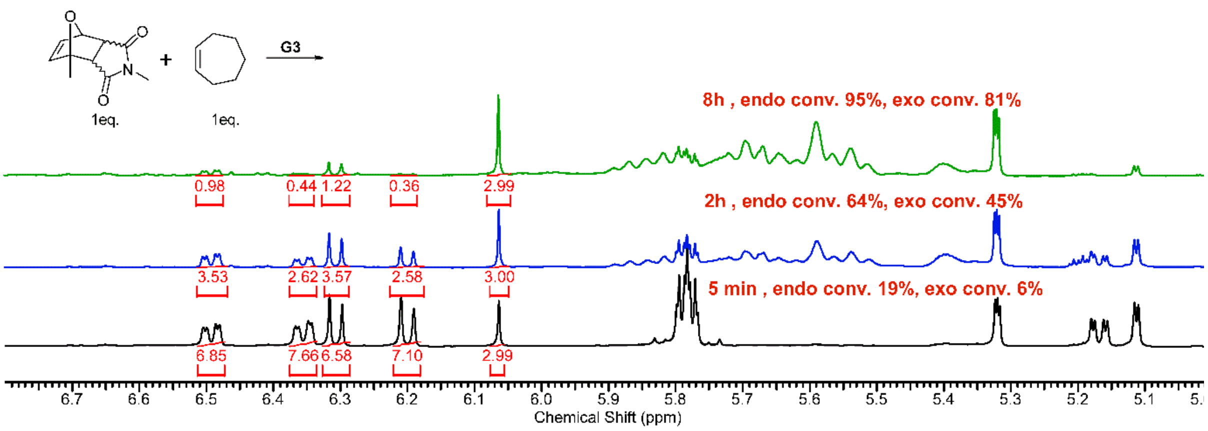

Fig S16. ^{1}H NMR spectra (CD_2Cl_2, 300MHz) of the copolymerization of **2** and cycloheptene with 6.6 mol% **G3** in the presence of the internal standard 1, 3, 5-trimethoxybenzene.

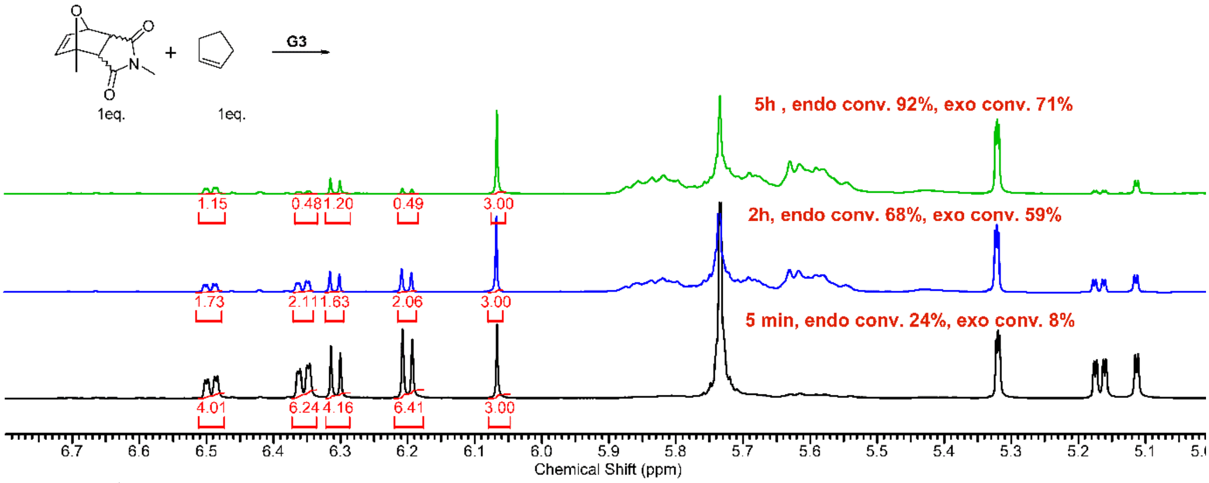

Fig S17. ^{1}H NMR spectra (CD_2Cl_2, 300MHz) of the copolymerization of **2** and cyclopentene with 6.6 mol% **G3** in the presence of the internal standard 1, 3, 5-trimethoxybenzene.

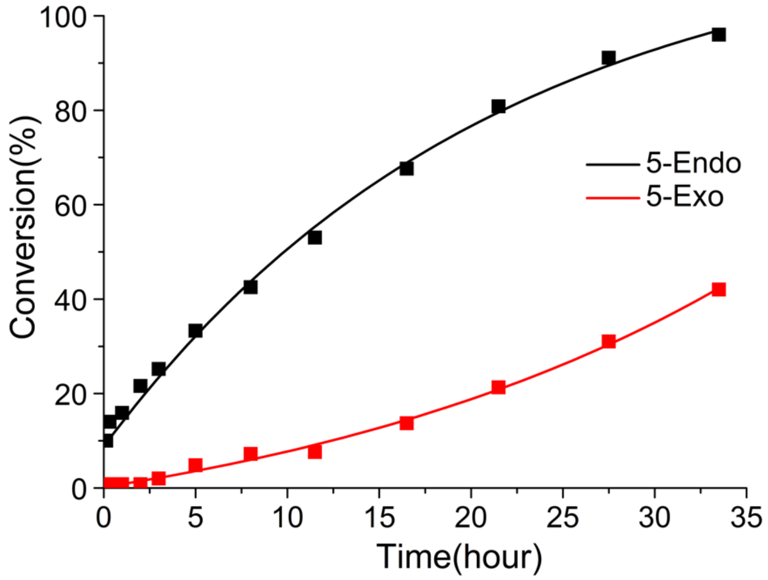

Fig S18. Plot of **5-*endo***, **5-*exo*** (70:30, 16 eq) conversion vs time in the copolymerization with cyclohexene (320 eq) and 6.6 mol% **G3** (1 eq).

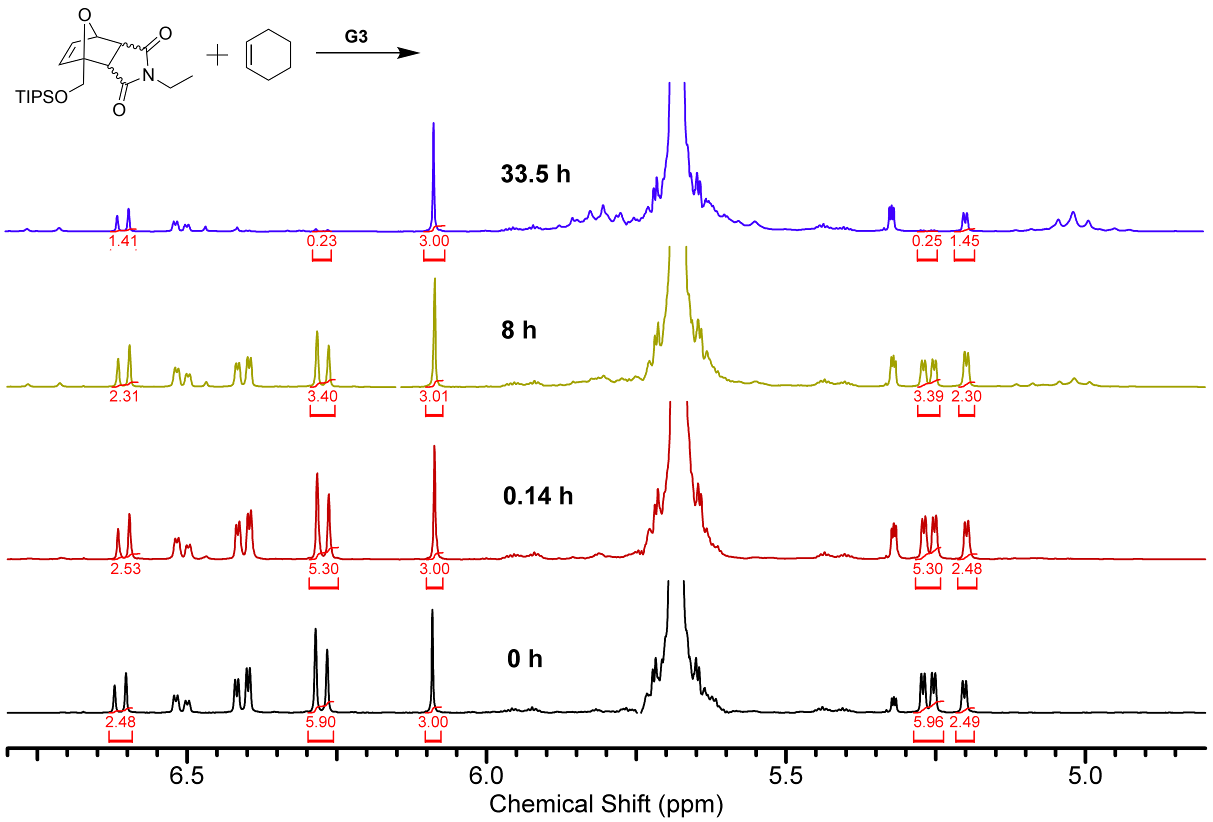

Fig S19. ^{1}H NMR spectra (CD_2Cl_2, 300MHz) of the copolymerization of **5** and cyclohexene with 6.6 mol% **G3** in the presence of the internal standard 1, 3, 5-trimethoxybenzene.

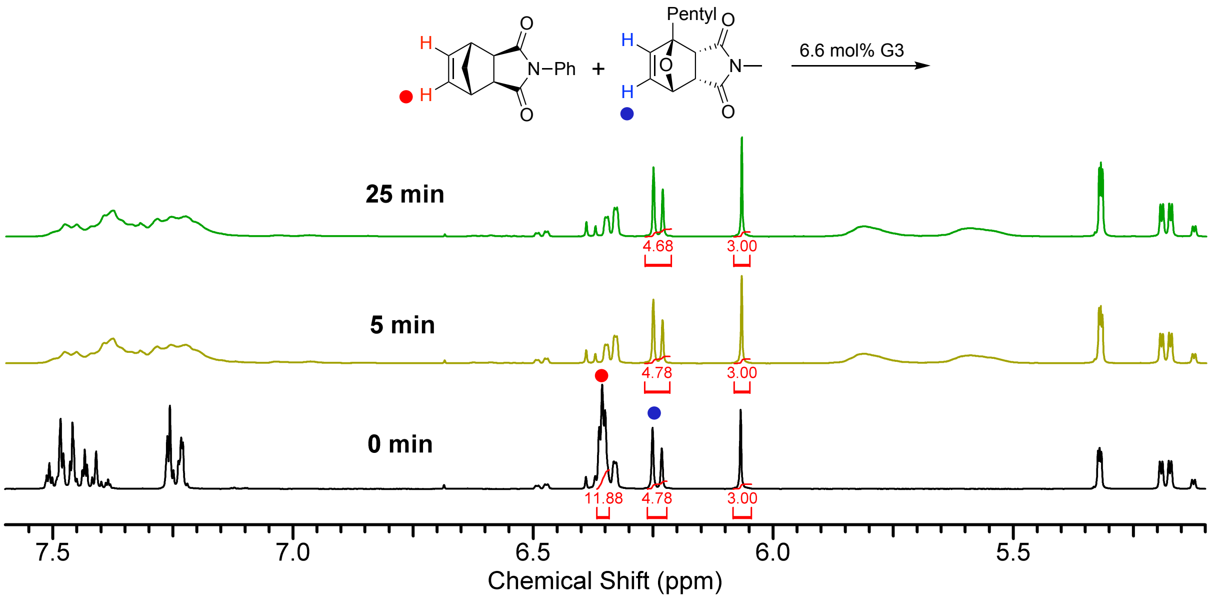

Fig S20. ^{1}H NMR spectra (CD_2Cl_2, 300MHz) of the copolymerization of equivalent mixture of **exo-*N*-phenylnorborneneimide** and **4-*endo*** with 6.6 mol% **G3** in the presence of the internal standard 1, 3, 5-trimethoxybenzene. Complete consumption of **exo-*N*-phenylnorborneneimide** was observed within 5 min and **4-*endo*** remain unreacted.

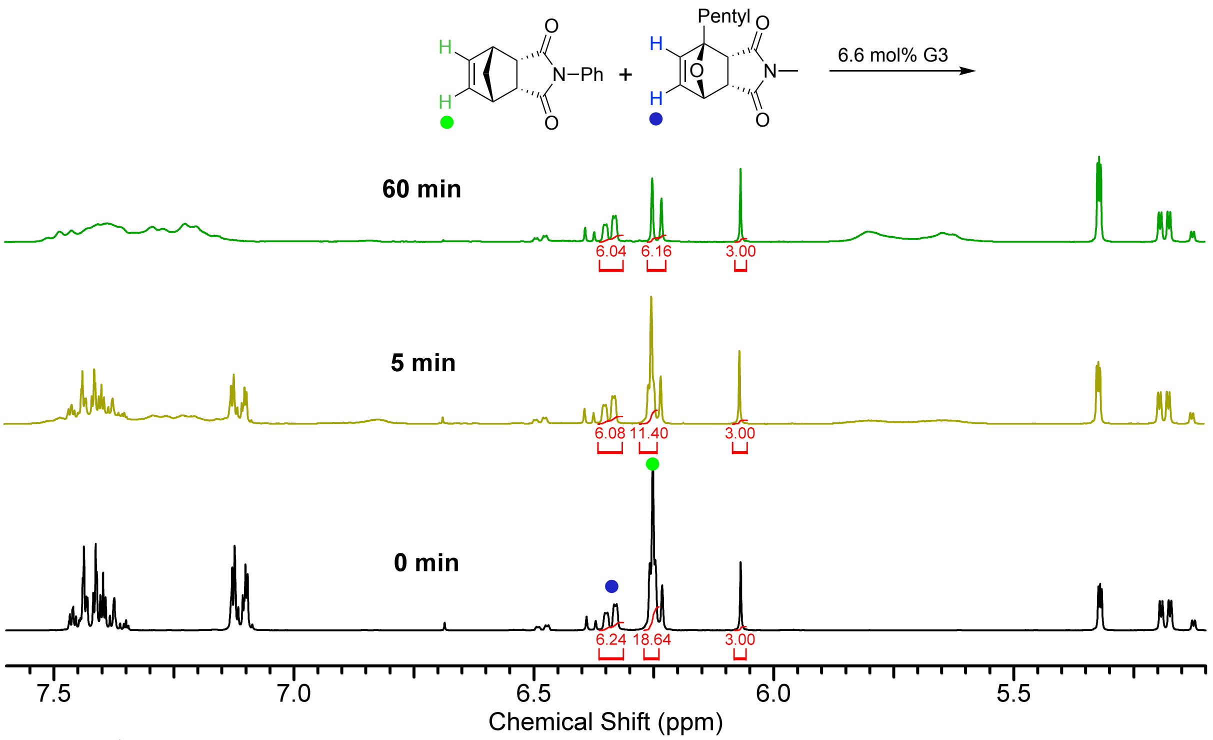

Fig S21. ^{1}H NMR spectra (CD_2Cl_2, 300MHz) of the copolymerization of equivalent mixture of **endo-*N*-phenylnorborneneimide** and **4-*endo*** with 6.6 mol% **G3** in the presence of the internal standard 1, 3, 5-trimethoxybenzene. Complete consumption of **endo- *N*-phenylnorborneneimide** was observed within 60 min and **4-*endo*** remain unreacted.

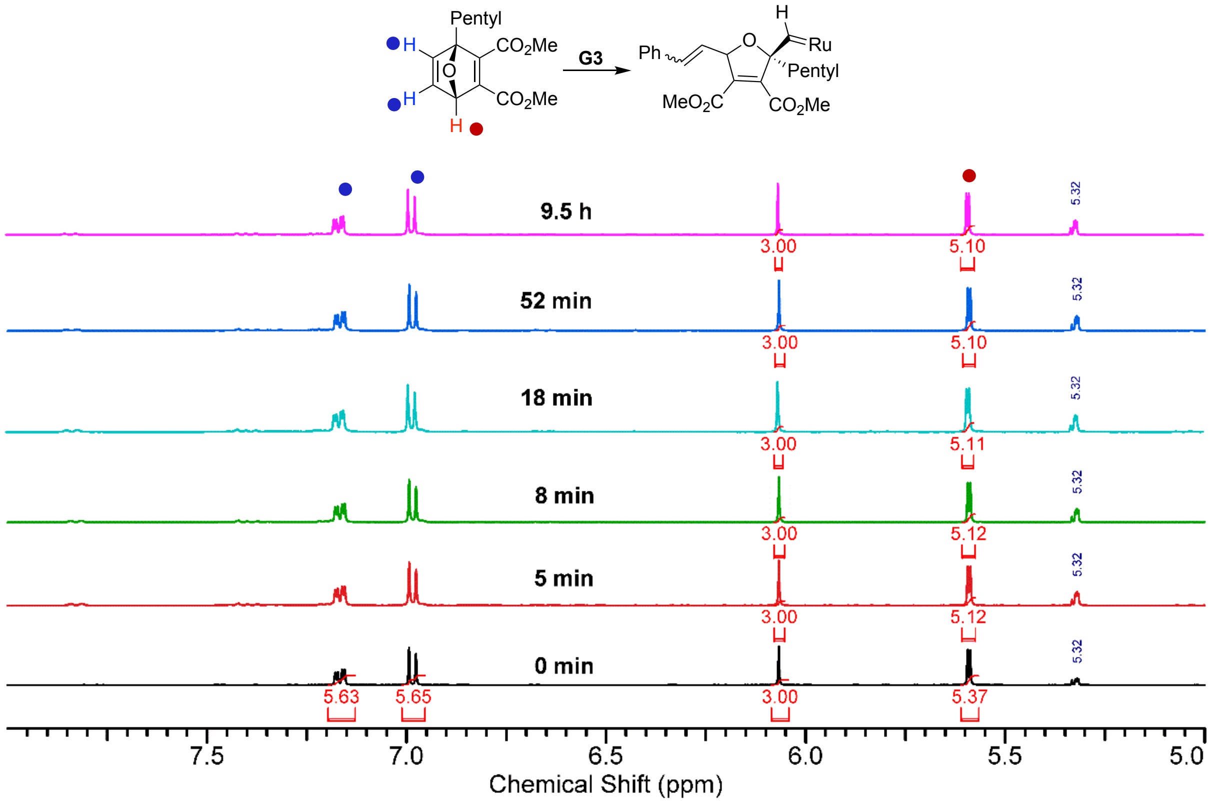

Fig S22. ^{1}H NMR spectra (CD_2Cl_2, 300 MHz) of the reactions of **5** with 5 mol% **G3** in presence of internal standard 1, 3, 5 trimethoxy benzene.

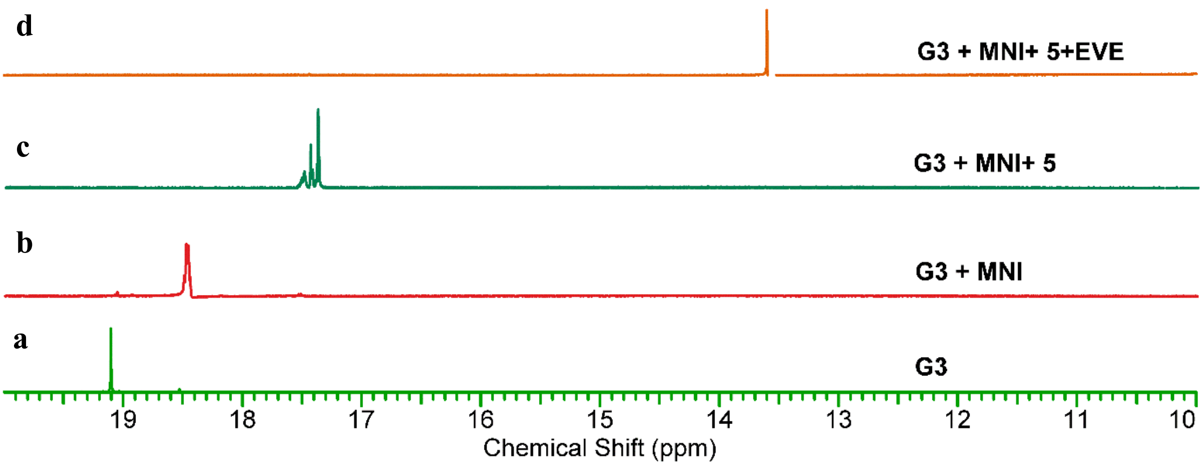

Fig S23. ^{1}H NMR spectra (CD_2Cl_2, 300 MHz) of the reactions of MNI, G3 and **5**. **(a)** Pure G3 benzylidene. (b) Alkylidene after complete consumption of MNI. (c) MNI alkylidene after reacting with 3eq of monomer **5**. (d) Fischer carbene after reacting with ethyl vinyl ether.

NMR spectra of monomers

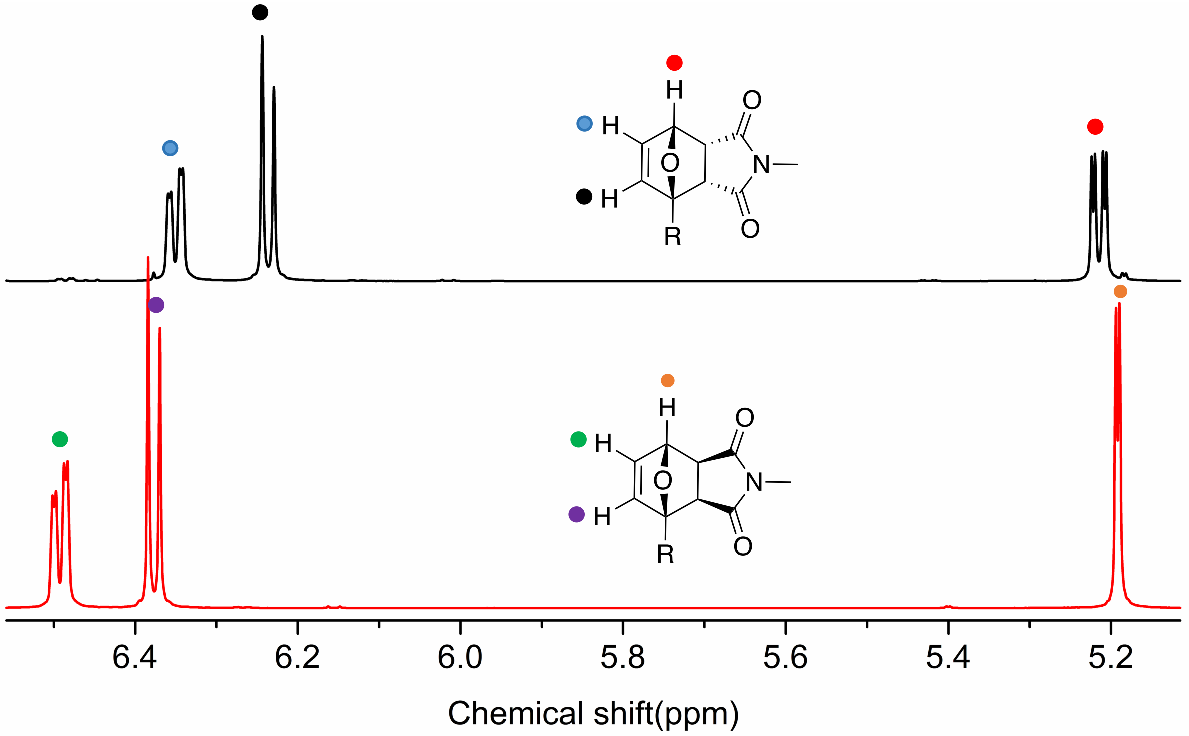

Fig S24. ^{1}H NMR (chloroform-d, 300 MHz) spectra of *endo* (top) and *exo* (bottom) *N*-methyl-7-oxanorborneneimide.

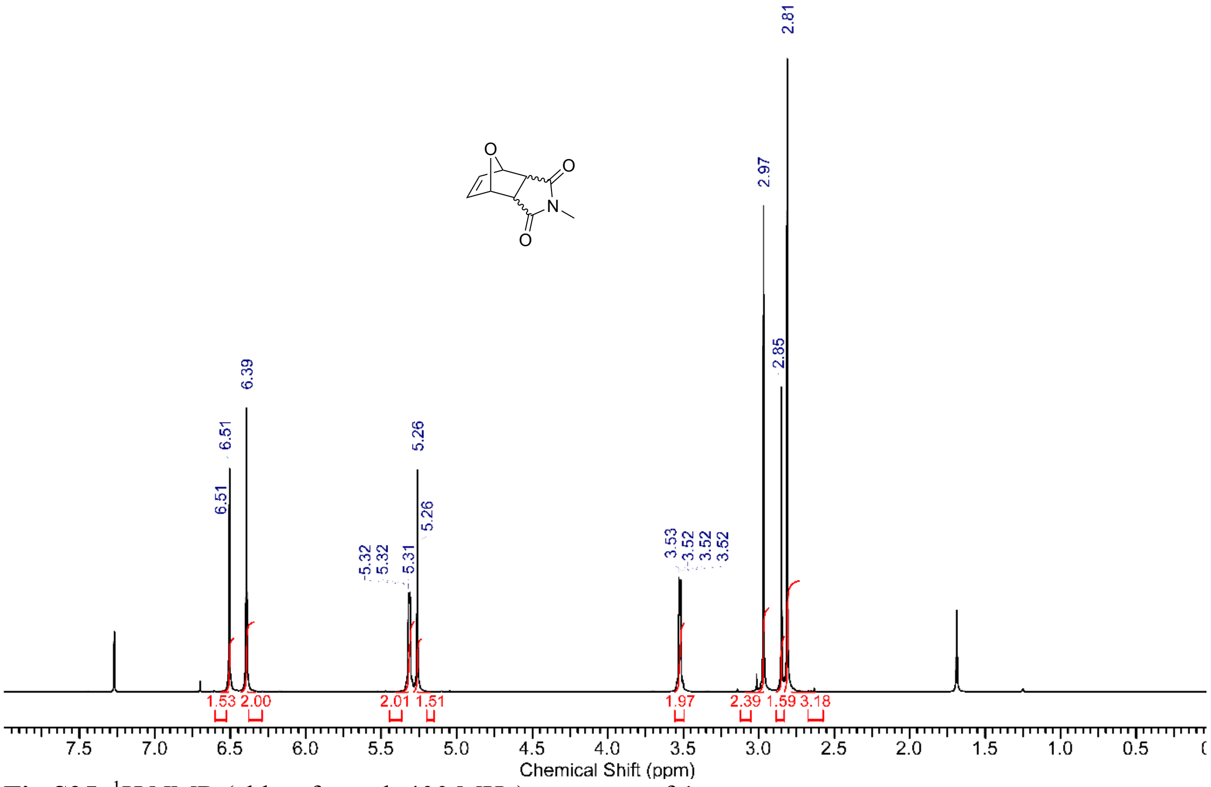

Fig S25. ^{1}H NMR (chloroform-d, 400 MHz) spectrum of **1**.

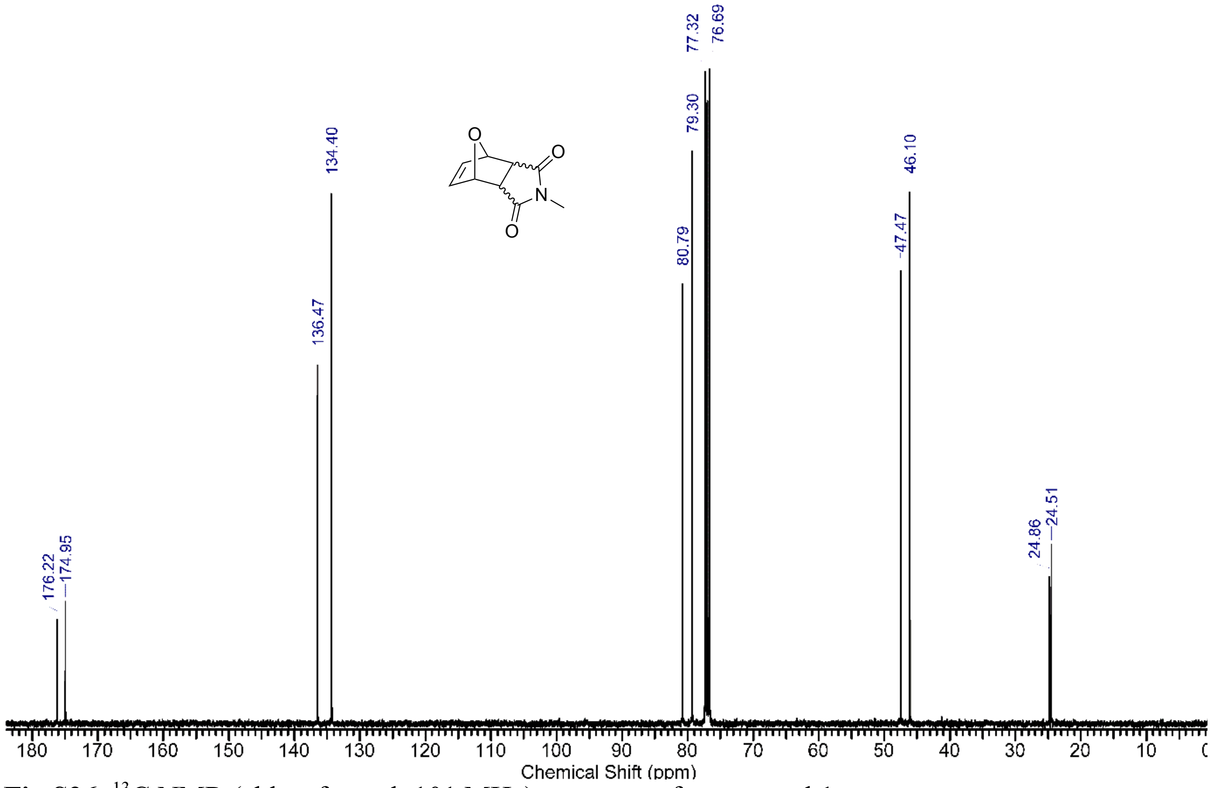

Fig S26. ^{13}C NMR (chloroform-d, 101 MHz) spectrum of compound **1**.

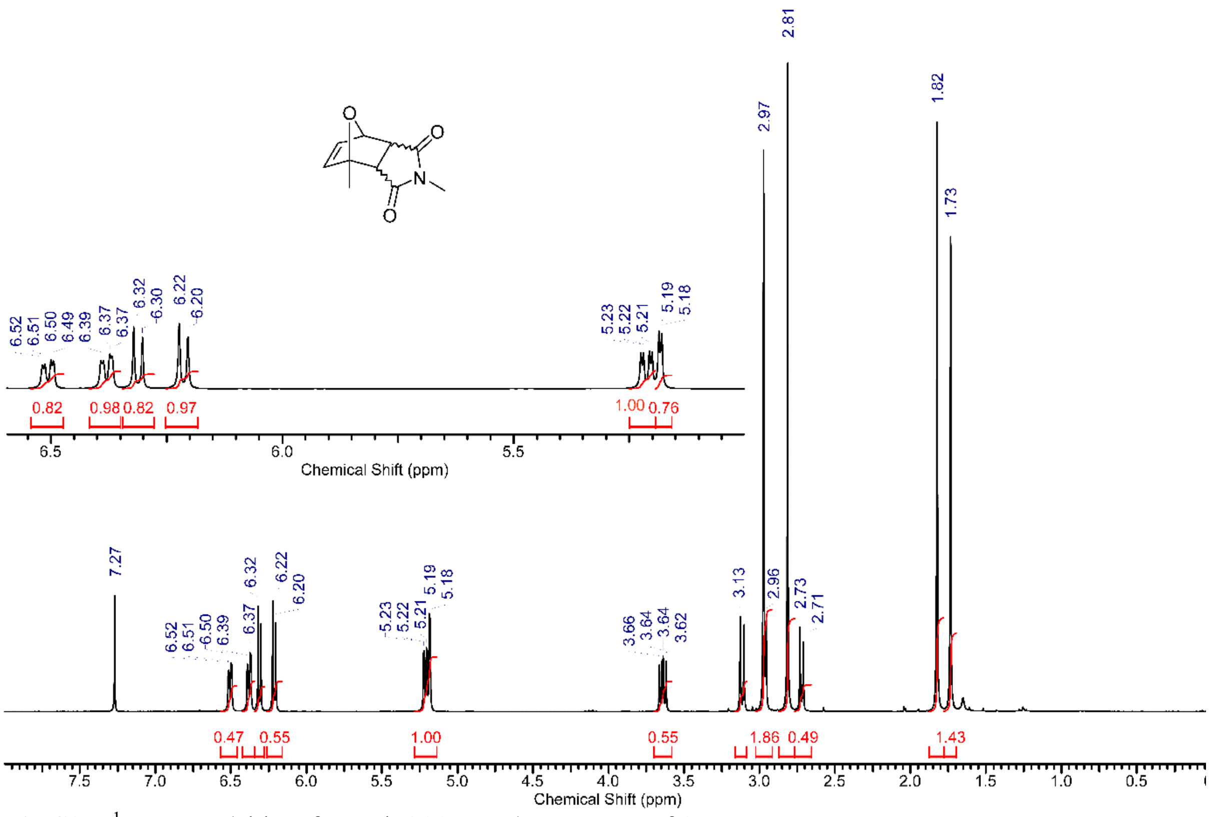

Fig S27. ^{1}H NMR (chloroform-d, 300 MHz) spectrum of **2**.

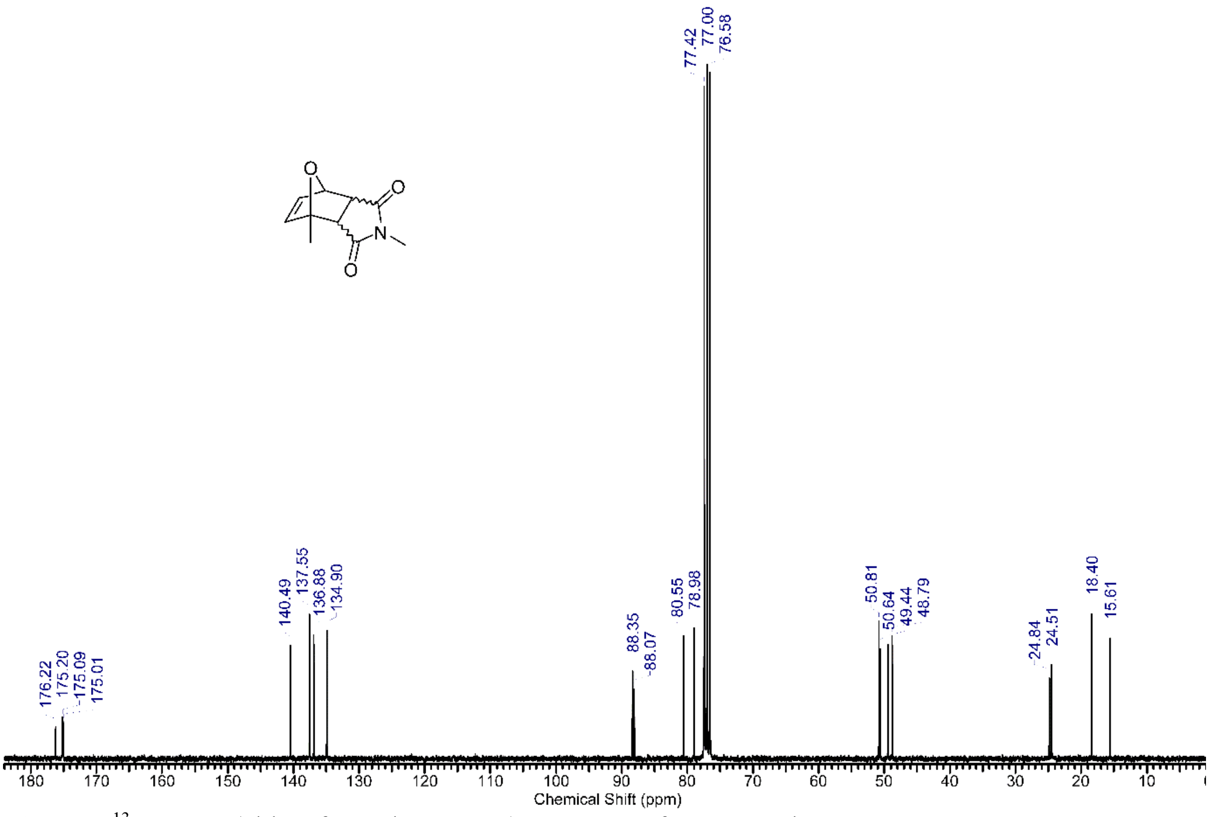

Fig S28. ^{13}C NMR (chloroform-d, 75 MHz) spectrum of compound **2**.

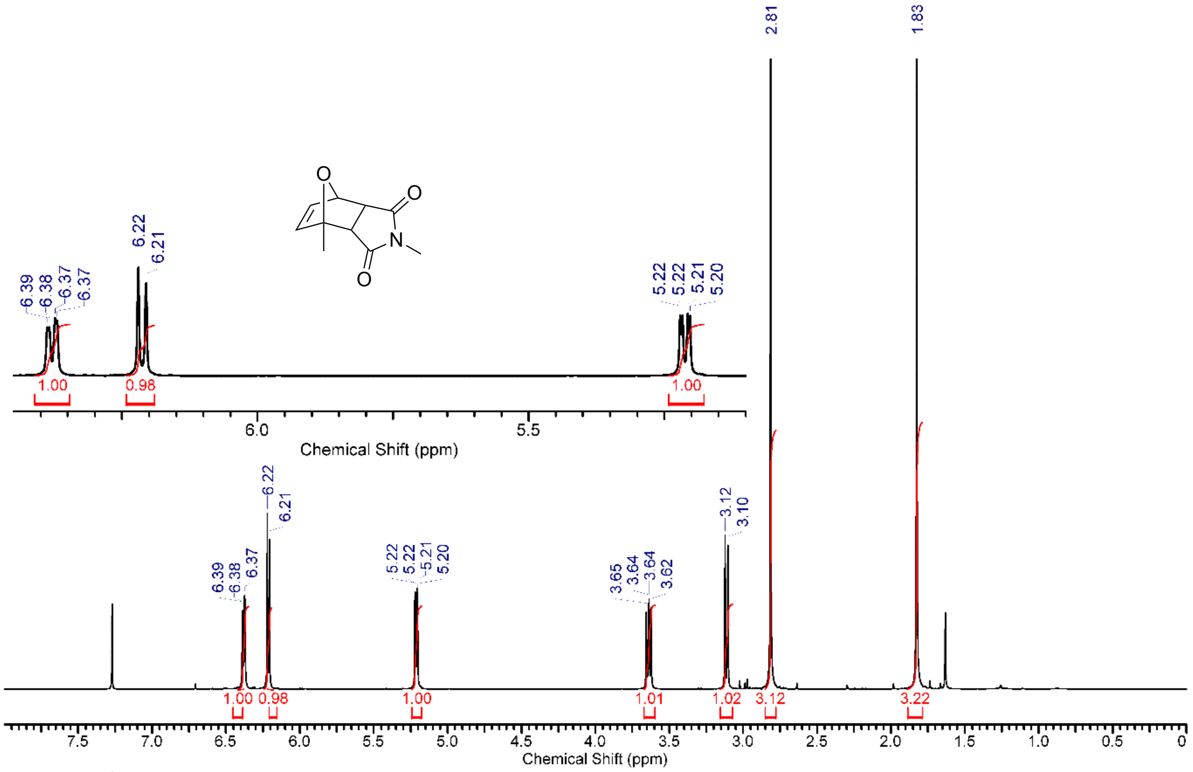

Fig S29. ^{1}H NMR (chloroform-d, 400 MHz) spectrum of **2-endo**.

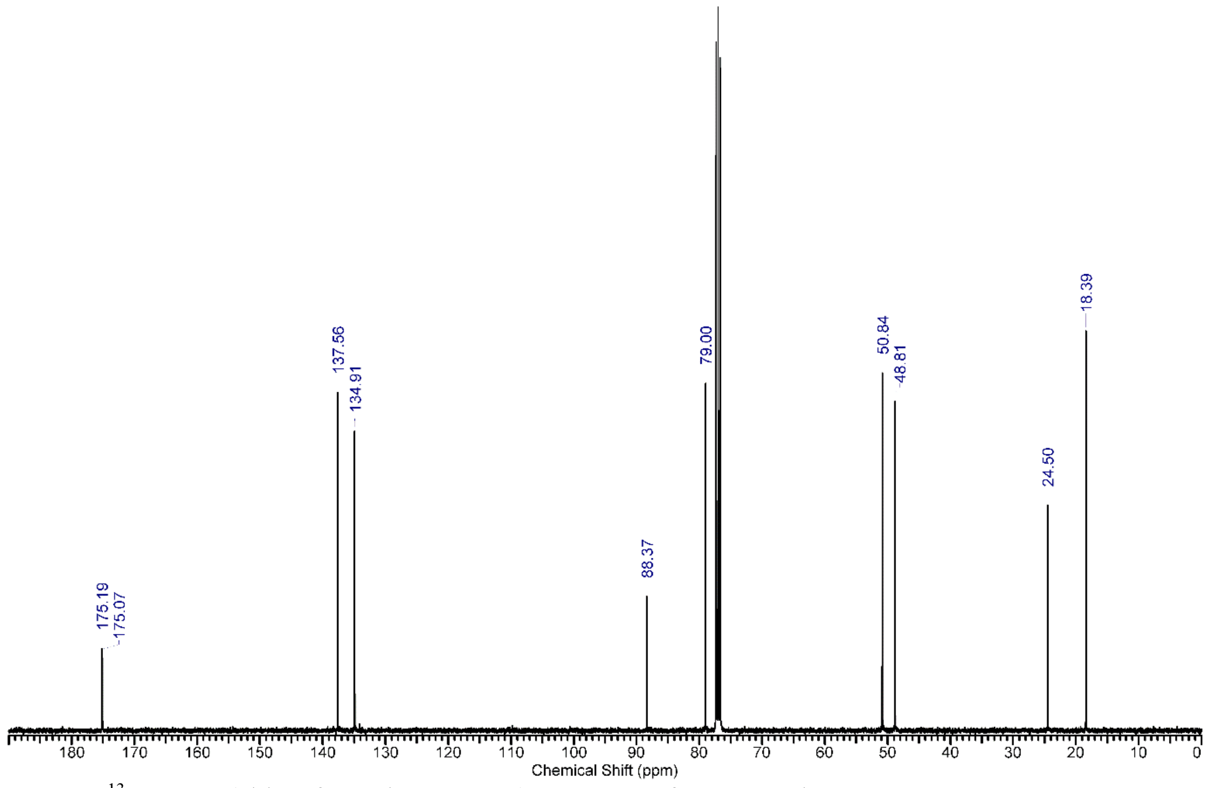

Fig S30. ^{13}C NMR (chloroform-d, 101 MHz) spectrum of compound **2-endo**.

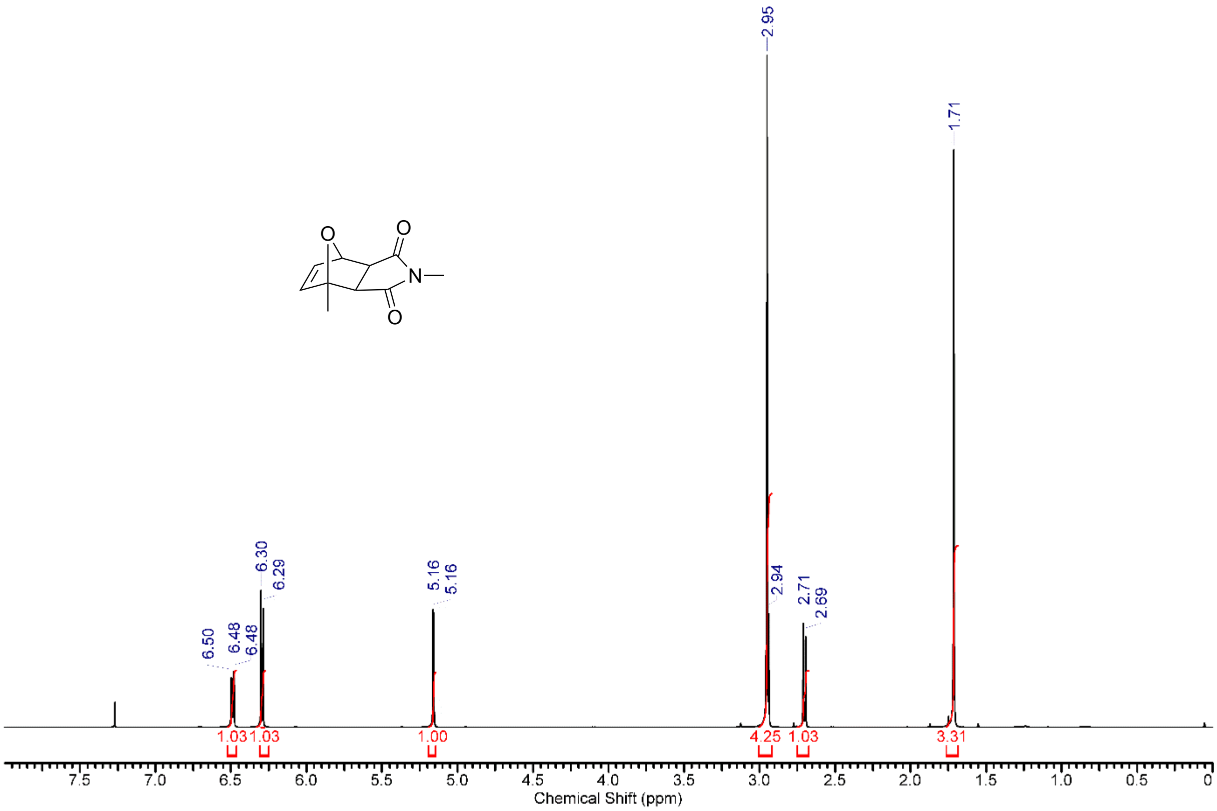

Fig S31. ^{1}H NMR (chloroform-d, 400 MHz) spectrum of **2-exo**.

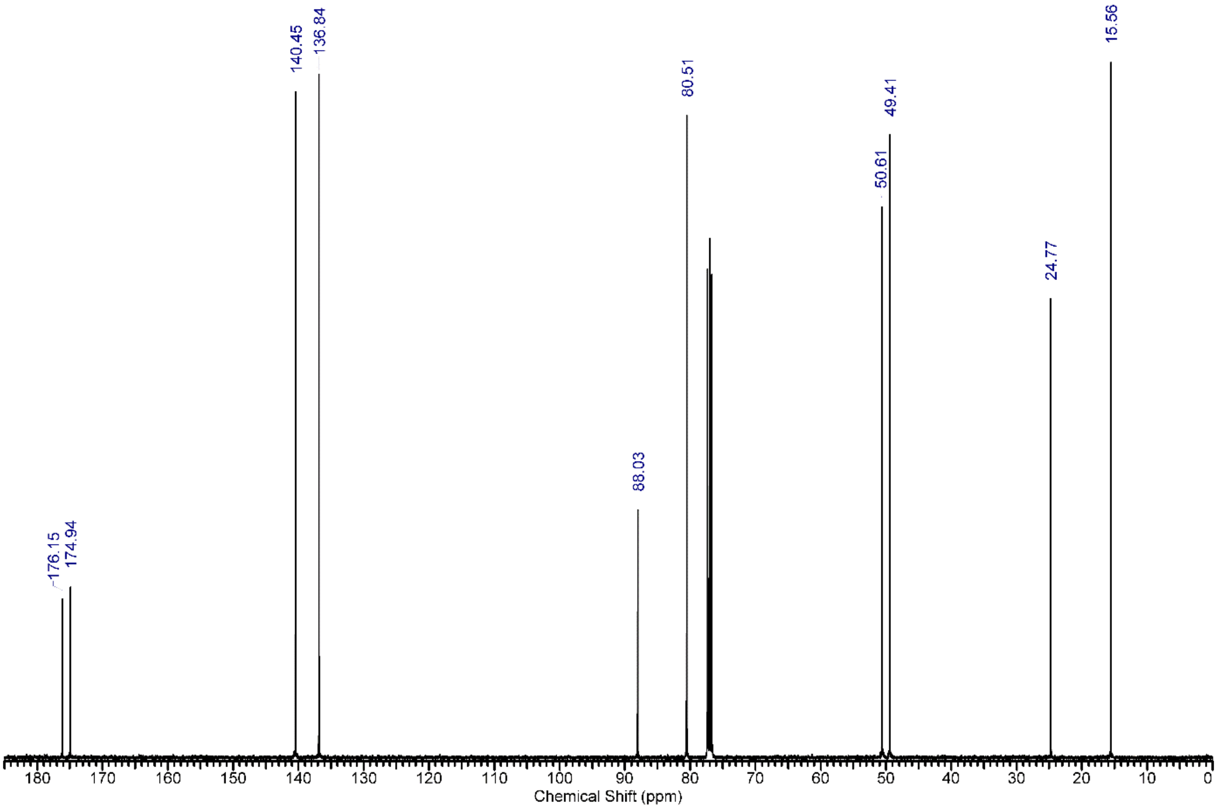

Fig S32. ^{13}C NMR (chloroform-d, 101 MHz) spectrum of compound **2-exo**.

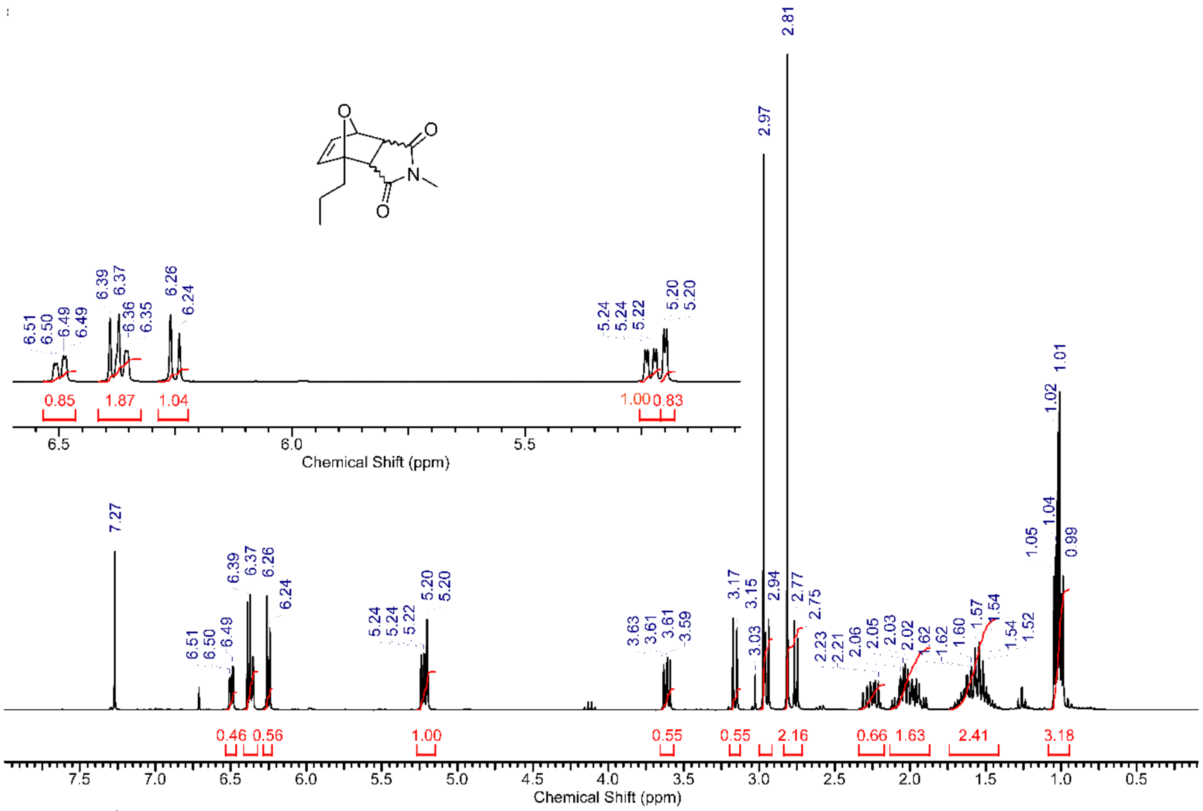

Fig S33. ^{1}H NMR (chloroform-d, 300 MHz) spectrum of **3.**

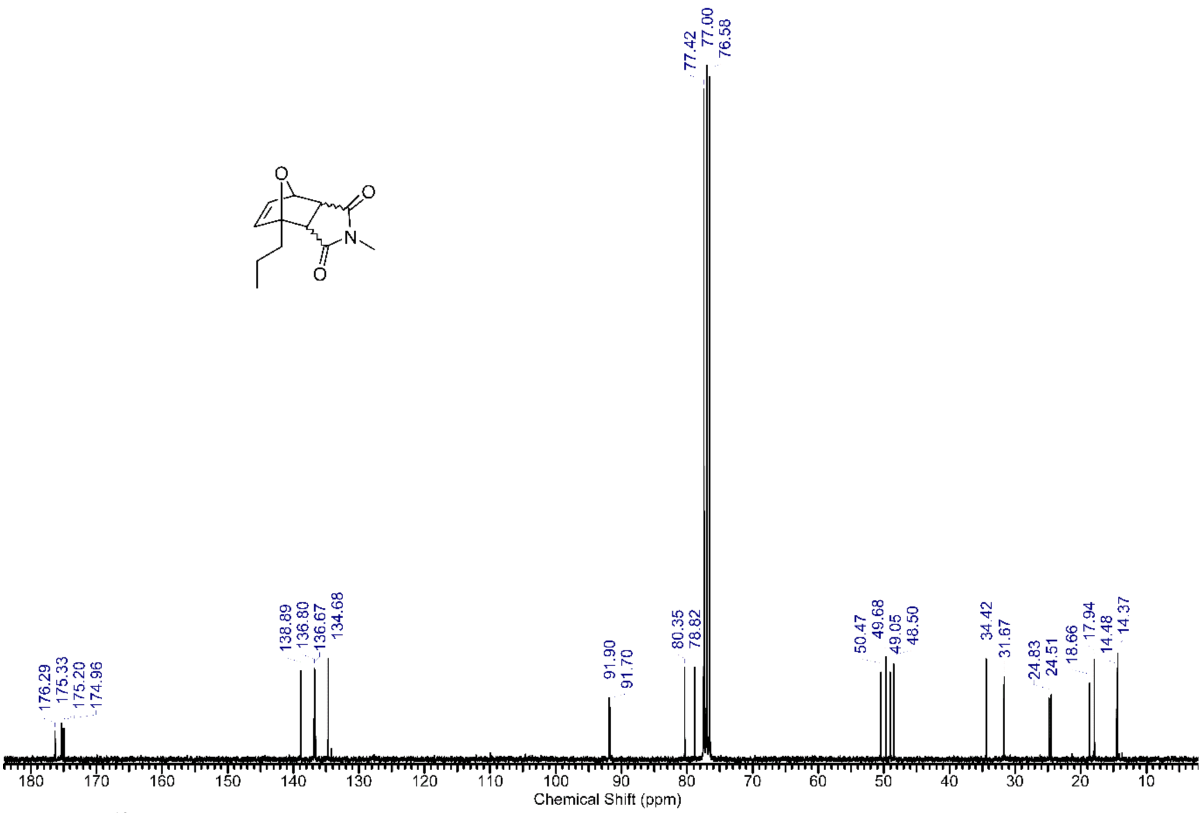

Fig S34. ^{13}C NMR (chloroform-d, 75 MHz) spectrum of compound **3.**

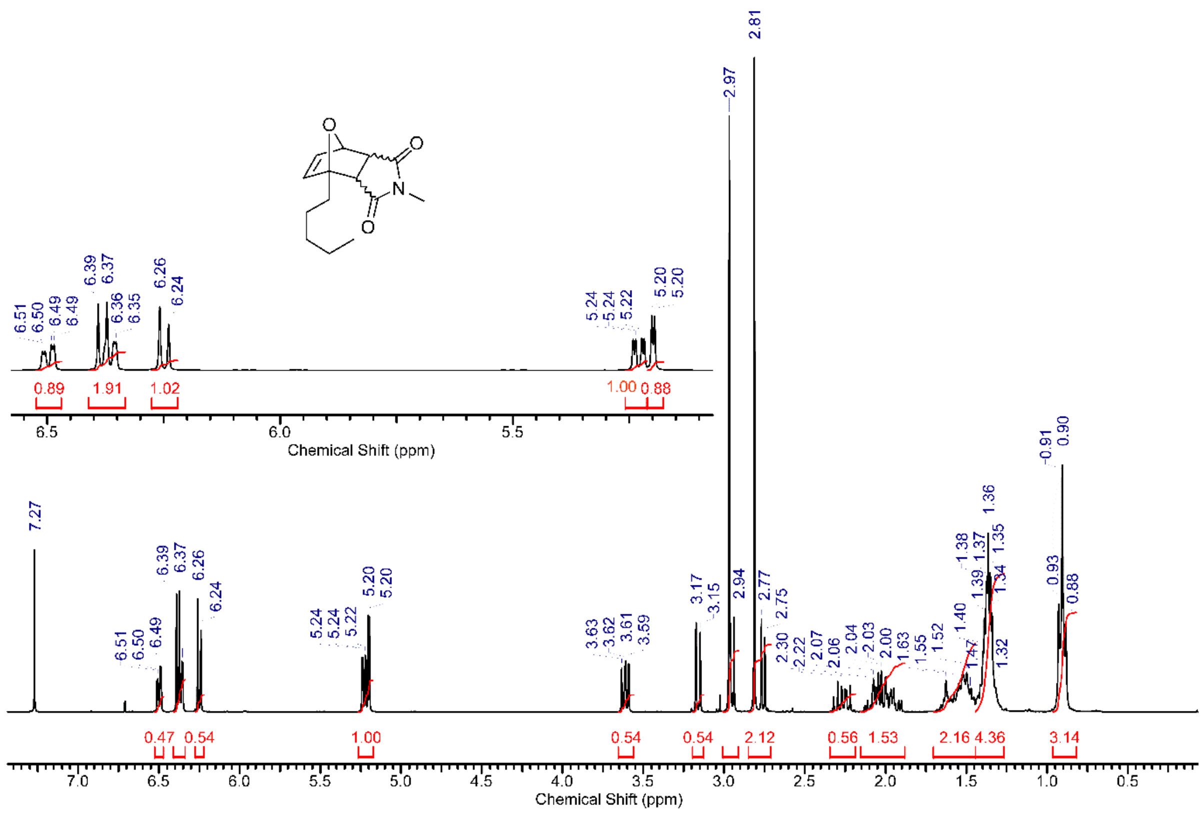

Fig S35. ^{1}H NMR (chloroform-d, 300 MHz) spectrum of **4.**

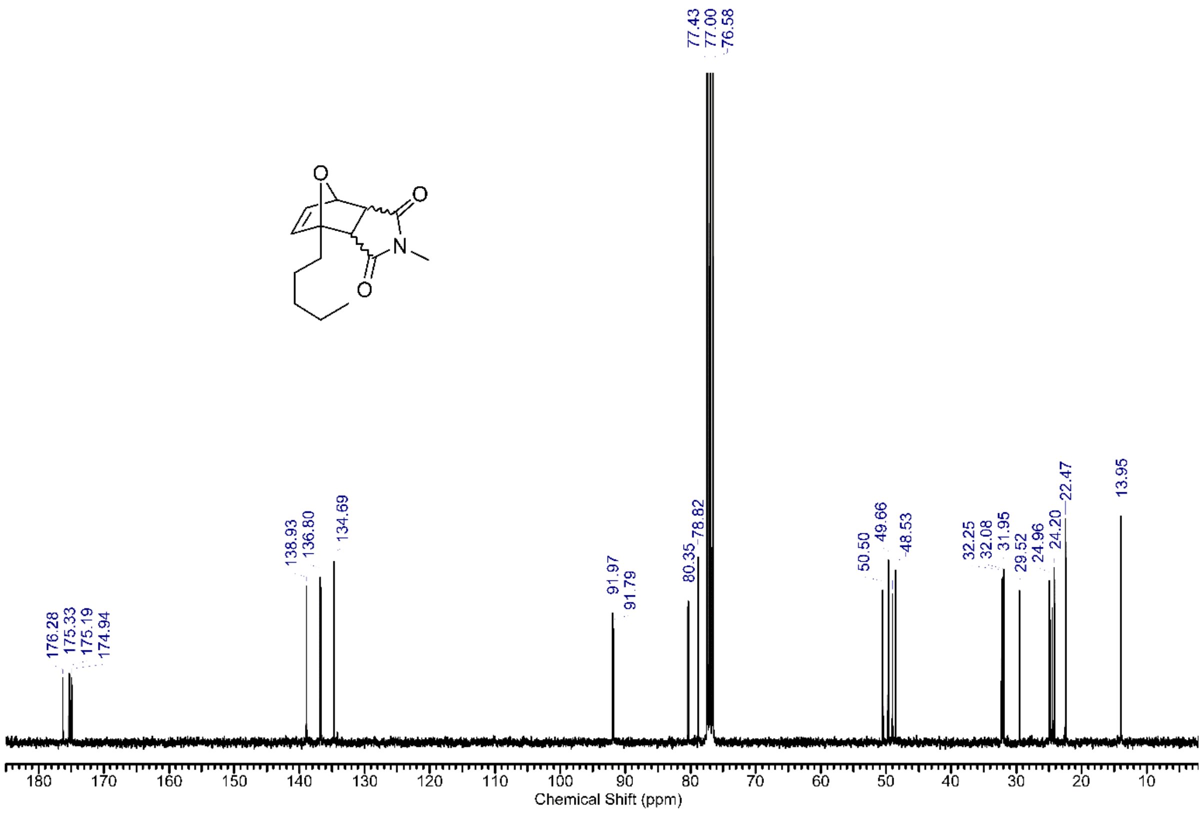

Fig S36. ^{13}C NMR (chloroform-d, 75 MHz) spectrum of compound **4.**

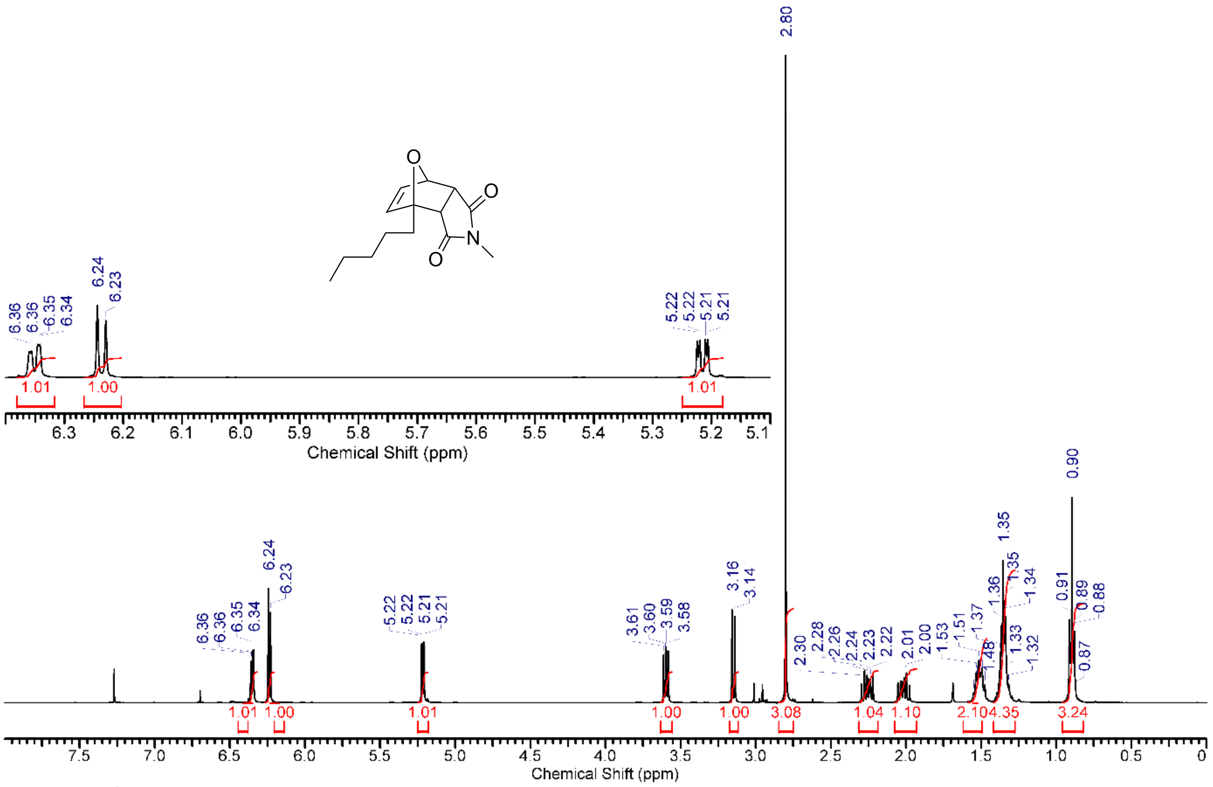

Fig S37. ^{1}H NMR (chloroform-d, 400 MHz) spectrum of **4-endo.**

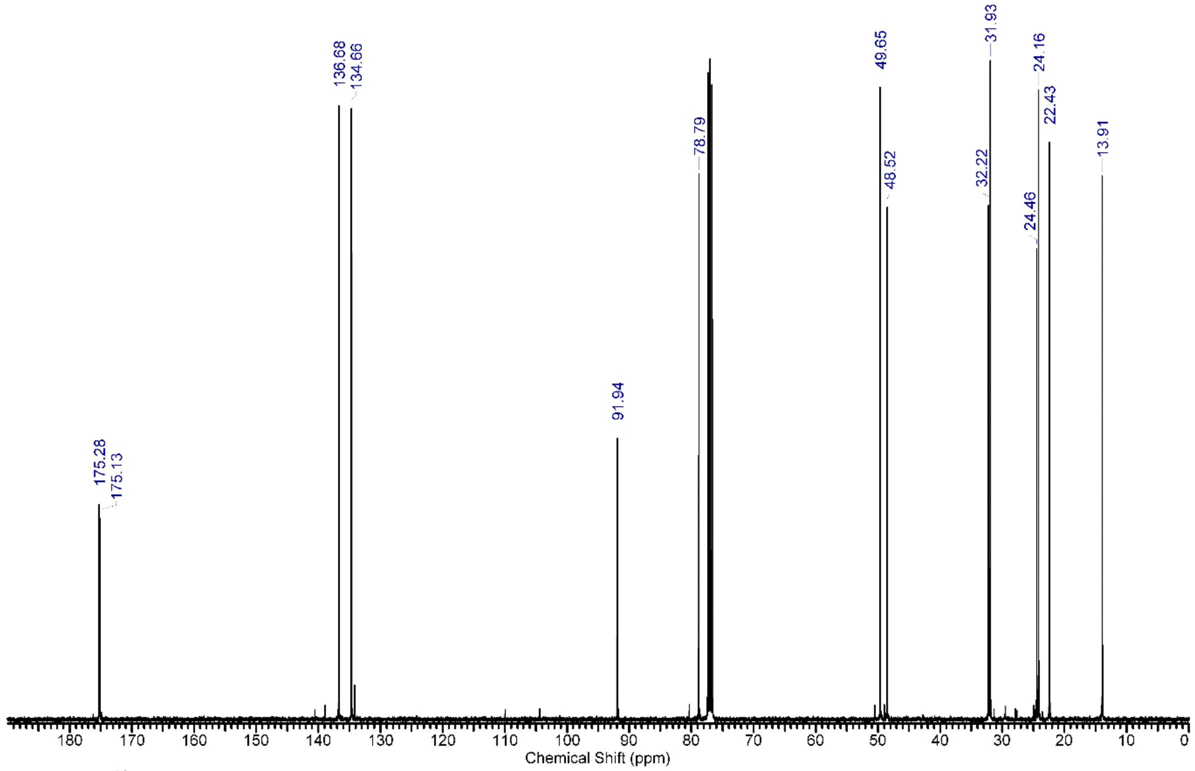

Fig S38. ^{13}C NMR (chloroform-d, 101 MHz) spectrum of compound **4-endo.**

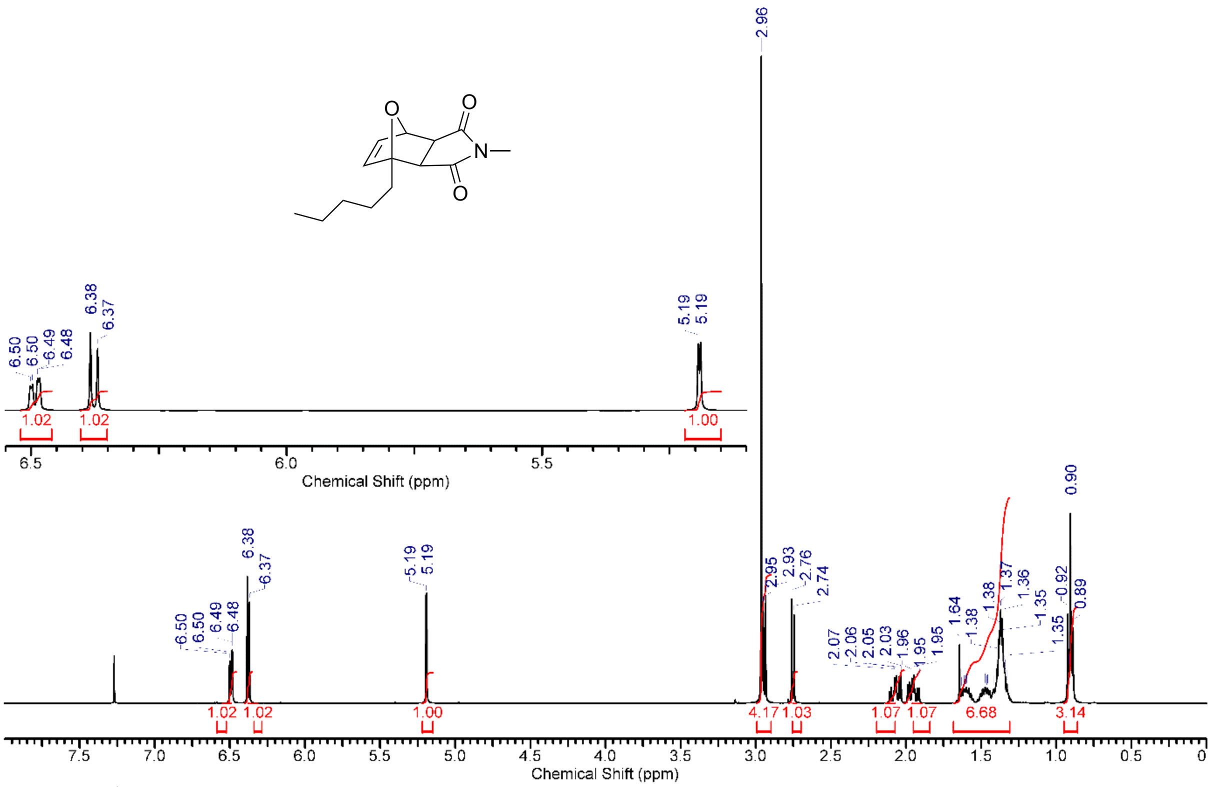

Fig S39. ^{1}H NMR (chloroform-d, 400 MHz) spectrum of **4-exo.**

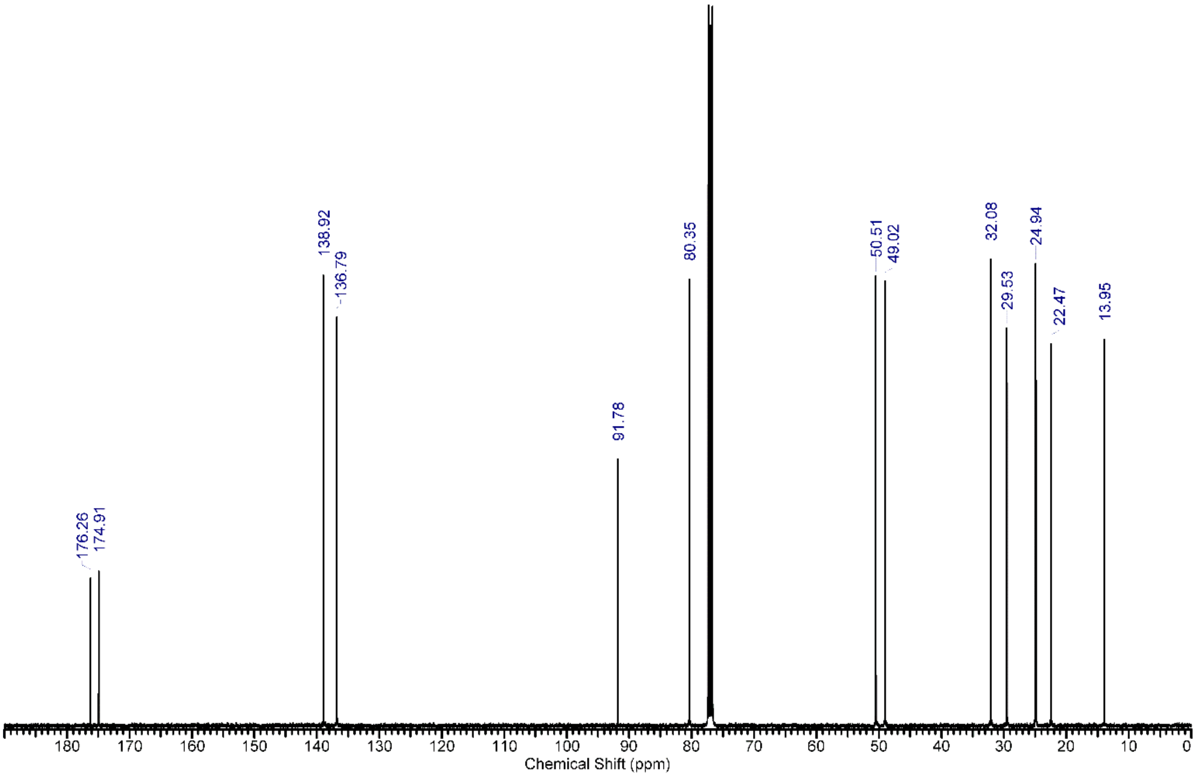

Fig S40. ^{13}C NMR (chloroform-d, 101 MHz) spectrum of compound **4-exo.**

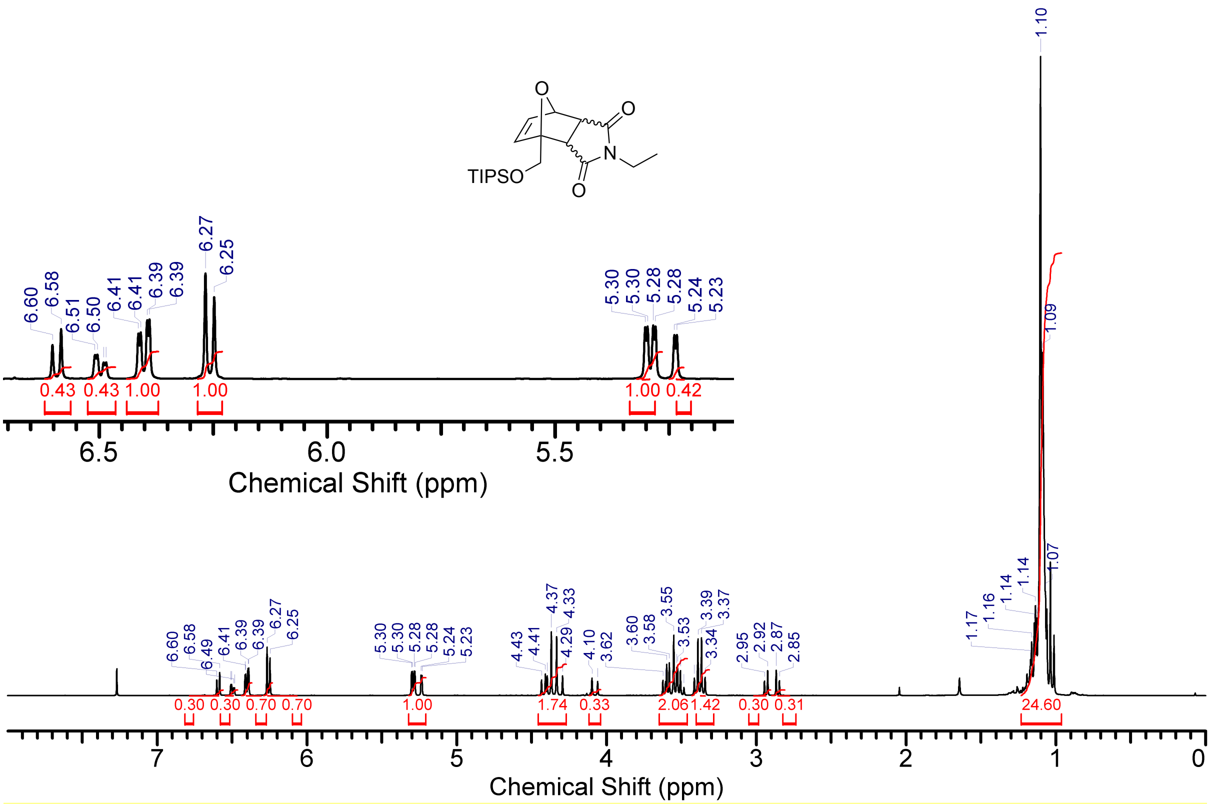

Fig S41. ^{1}H NMR (chloroform-d, 300 MHz) spectrum of **5.**

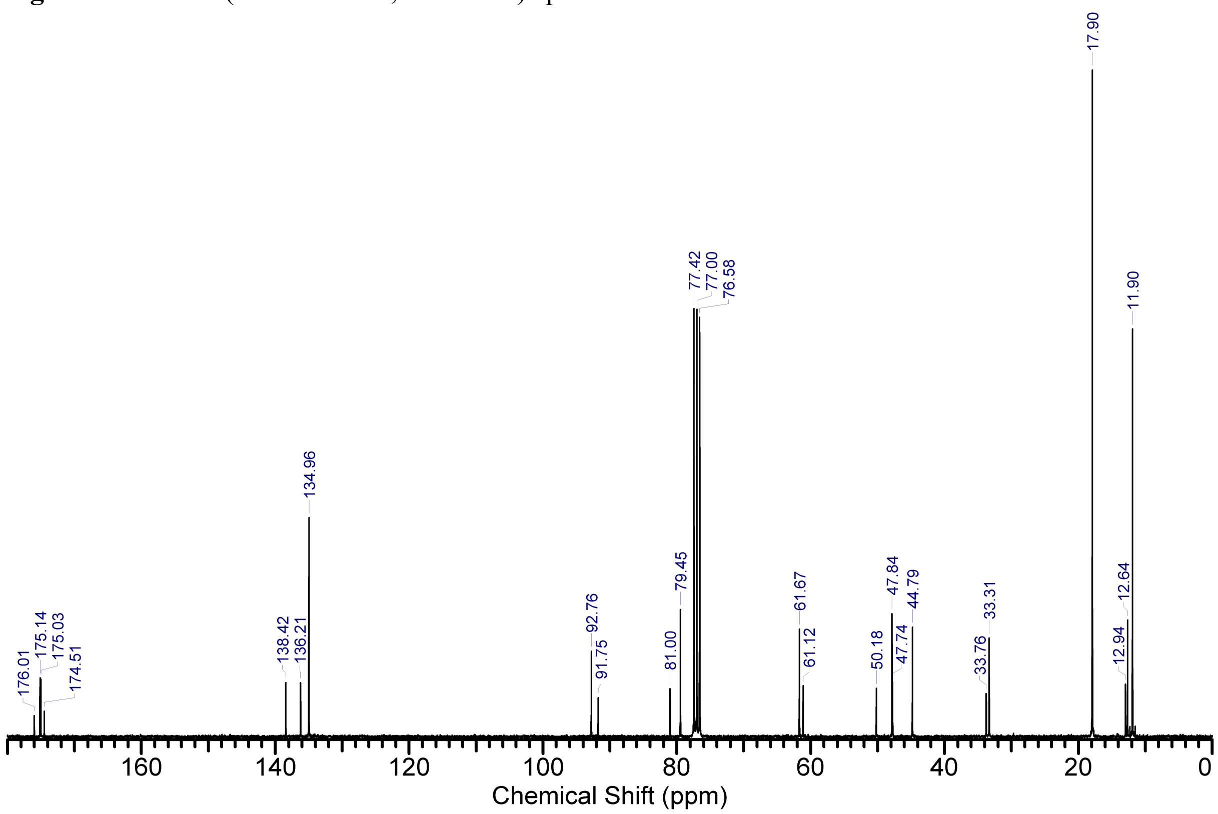

Fig S42. ^{13}C NMR (chloroform-d, 75 MHz) spectrum of compound **5.**

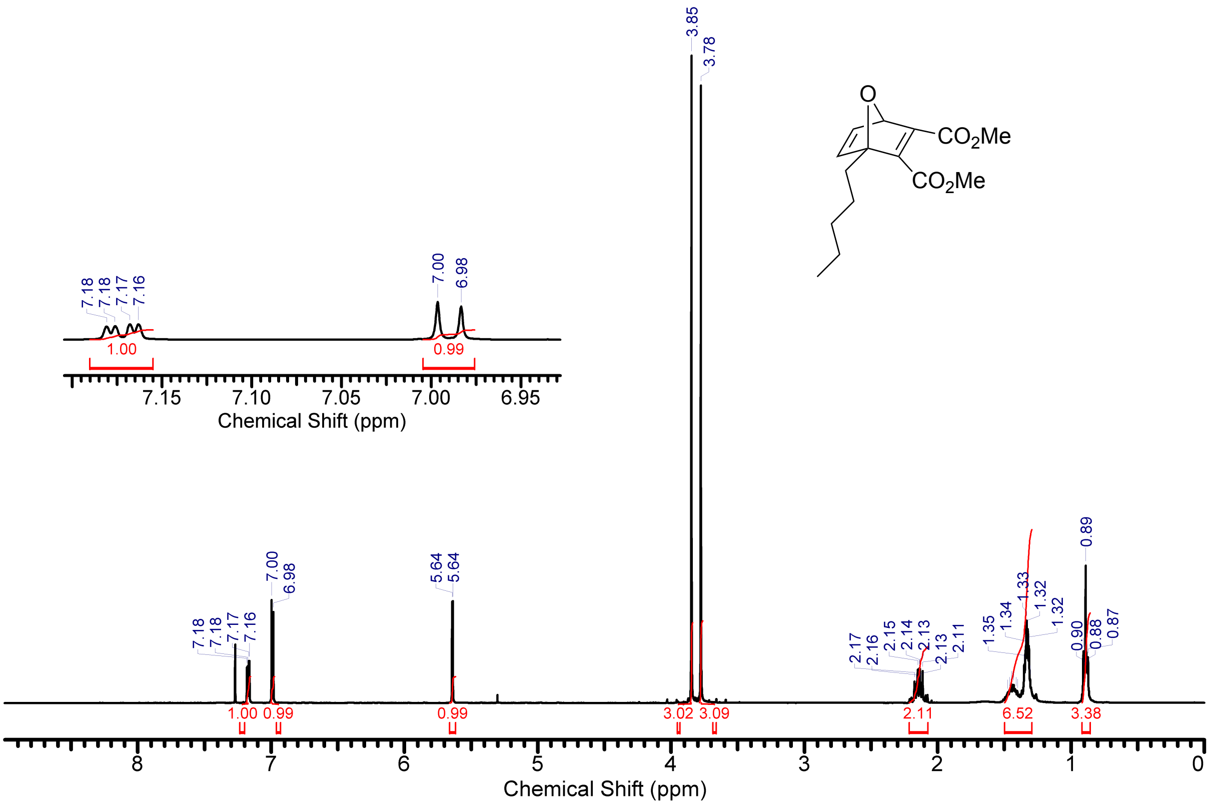

Fig S43. ^{1}H NMR (chloroform-d, 400 MHz) spectrum of **6.**

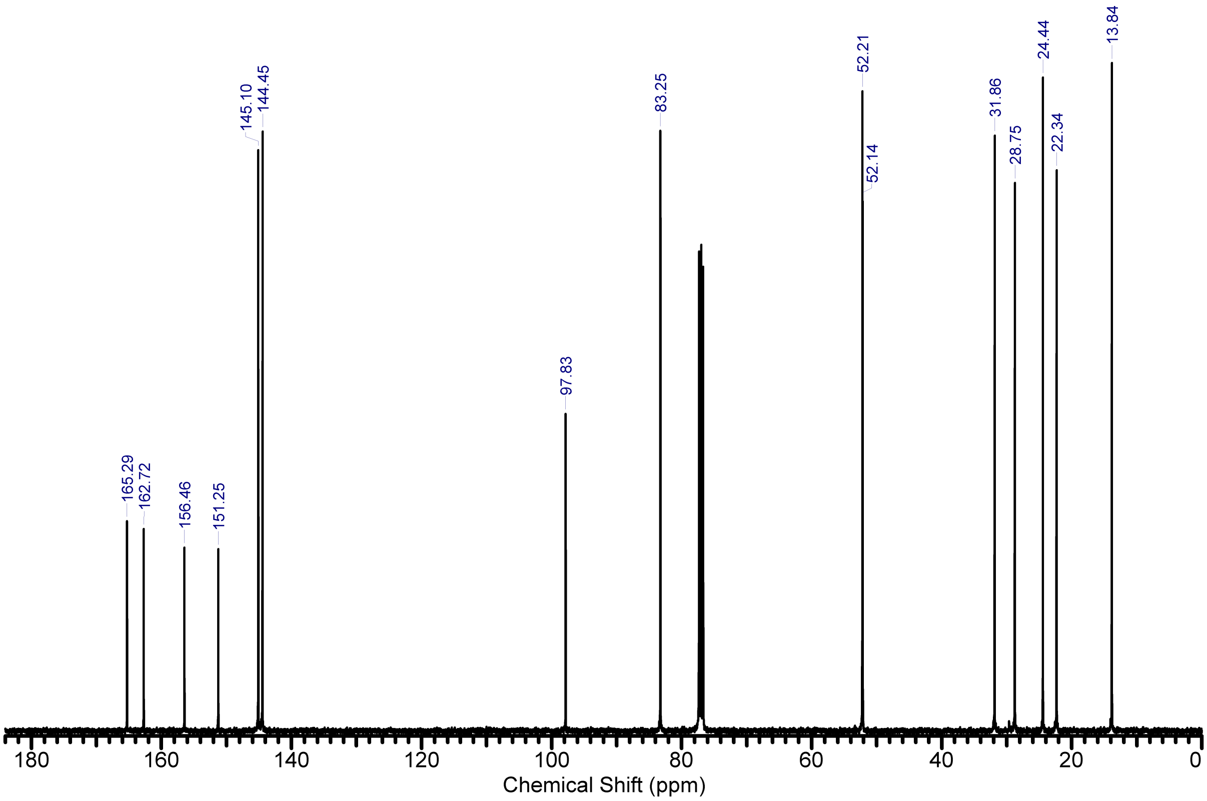

Fig S44. ^{13}C NMR (chloroform-d, 101 MHz) spectrum of compound **6.**

GPC elugrams of polymers

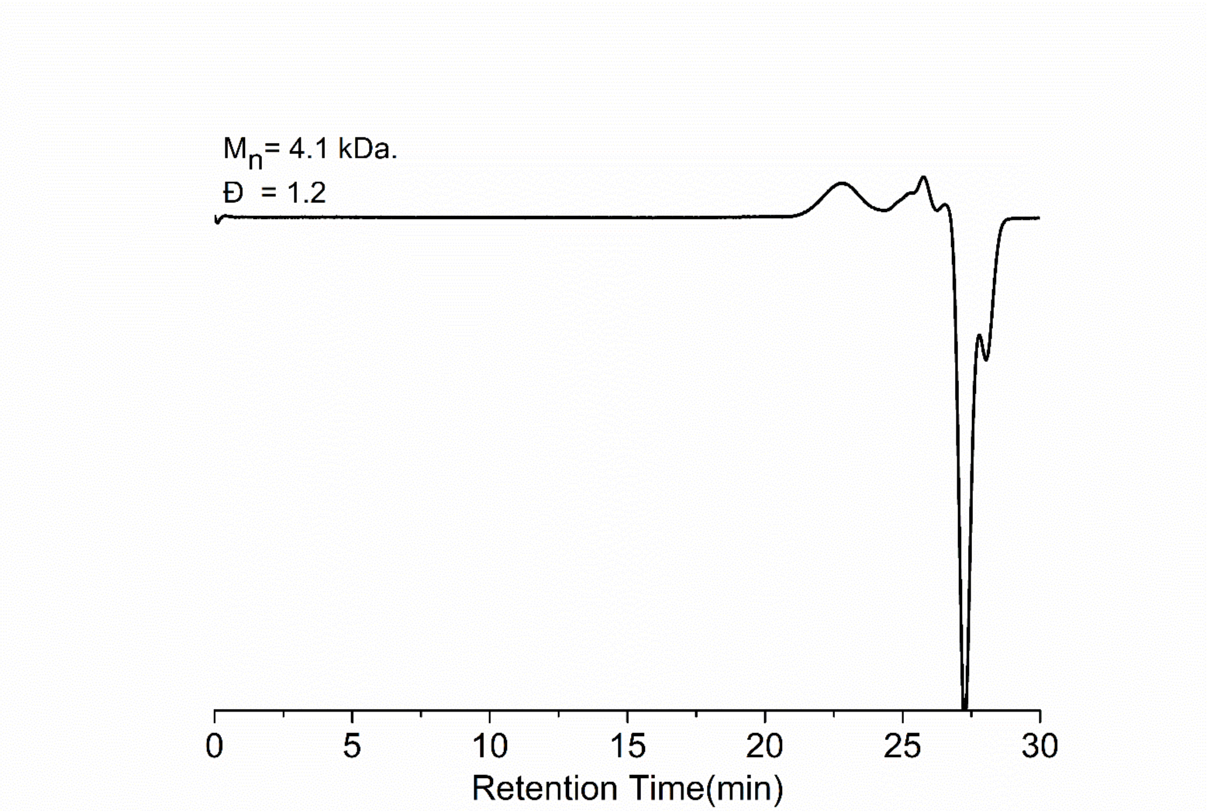

Fig S45. GPC (THF) trace of the crude polymer **P1.**

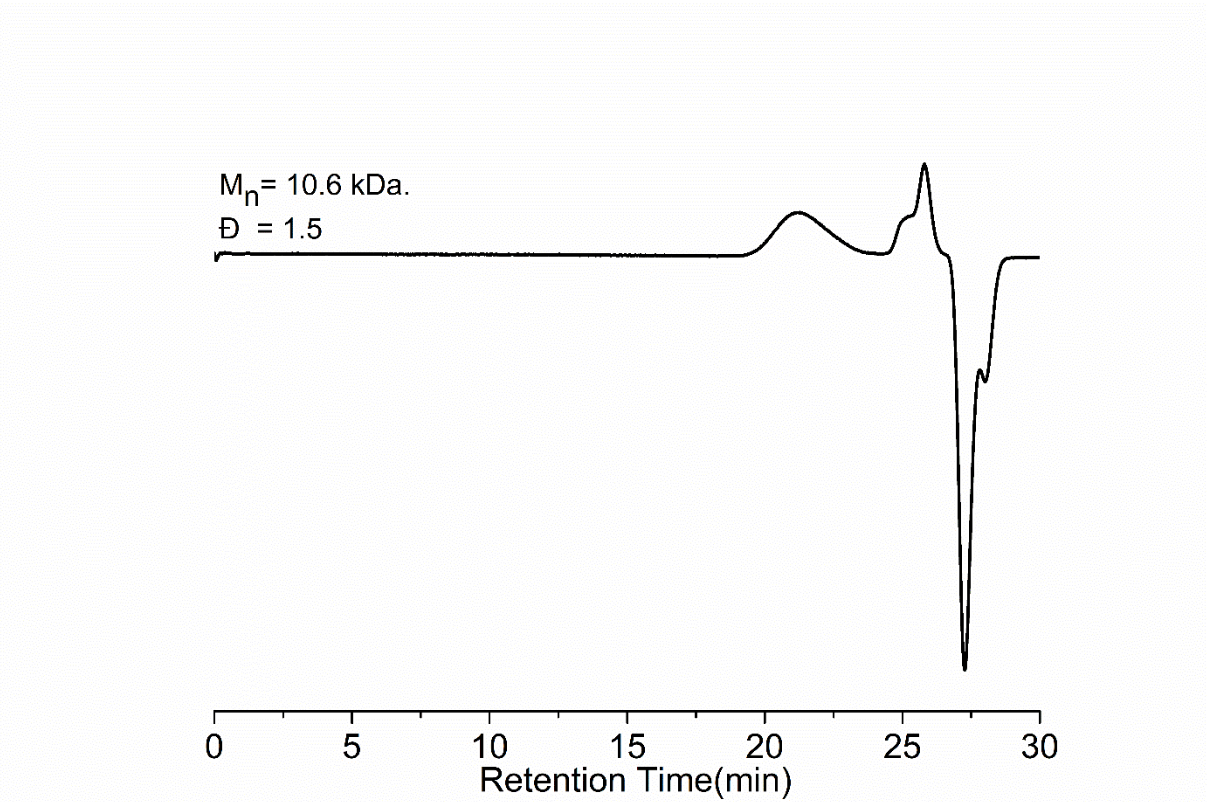

Fig S46. GPC (THF) trace of the crude polymer **P2.**

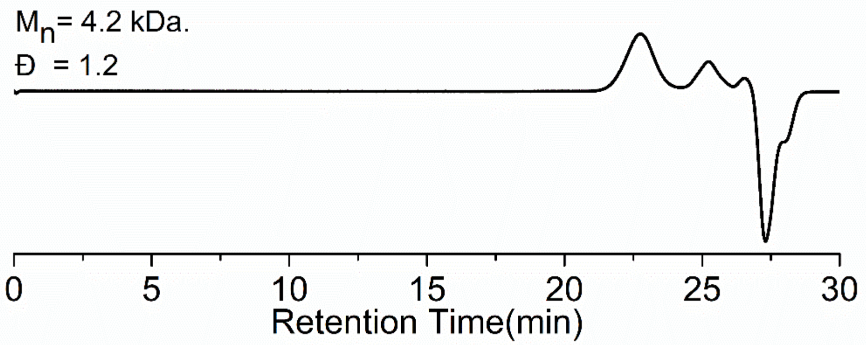

Fig S47. GPC (THF) trace of the crude polymer **P3.**

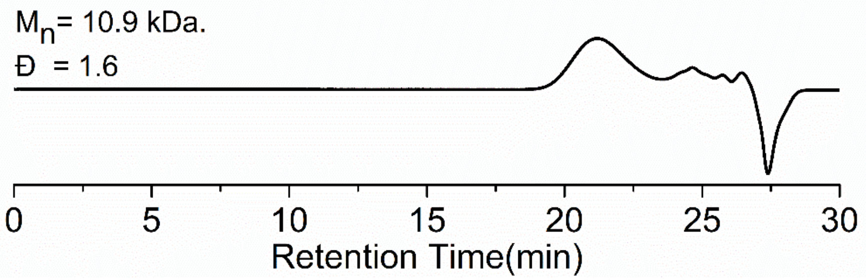

Fig S48. GPC (THF) trace of the crude polymer **P4.**

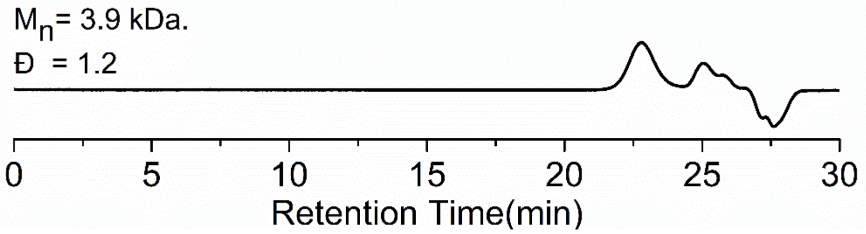

Fig S49. GPC (THF) trace of the crude polymer **P5.**

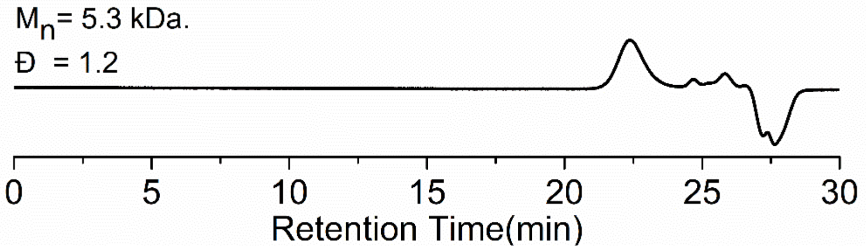

Fig S50. GPC (THF) trace of the crude polymer **P6.**

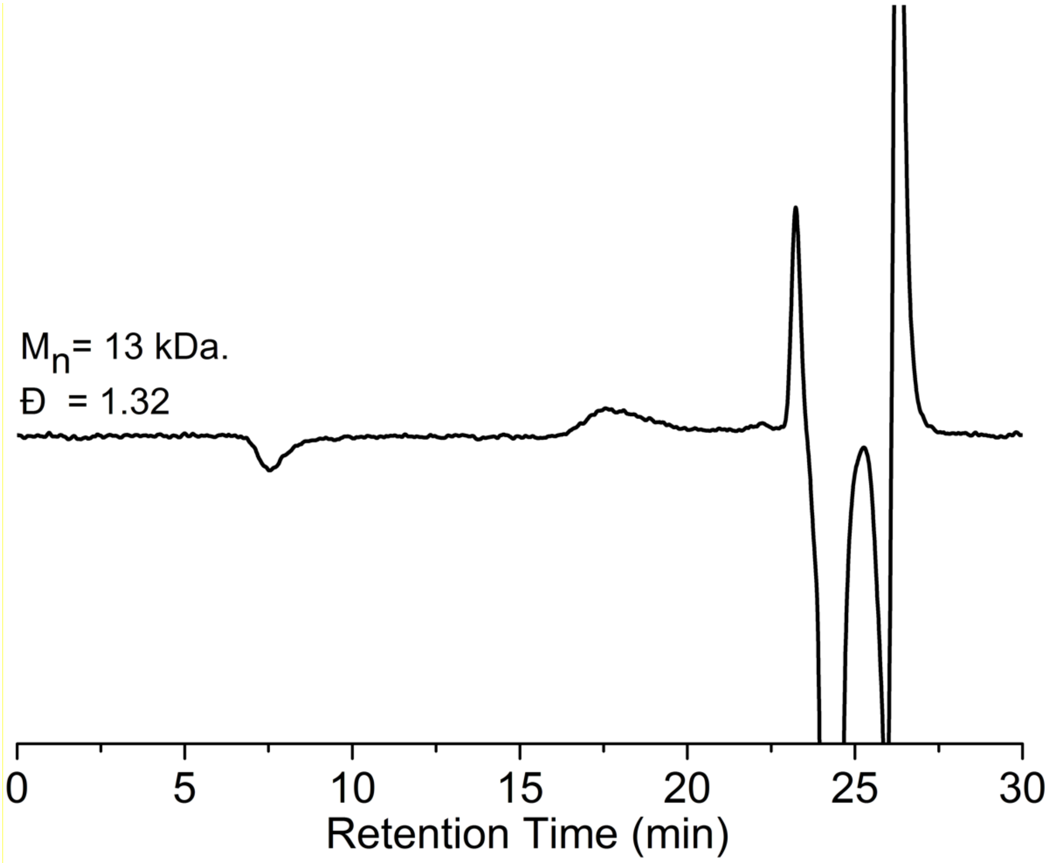

Fig S51. GPC ($CHCl_3$) trace of the crude polymer **P7.**

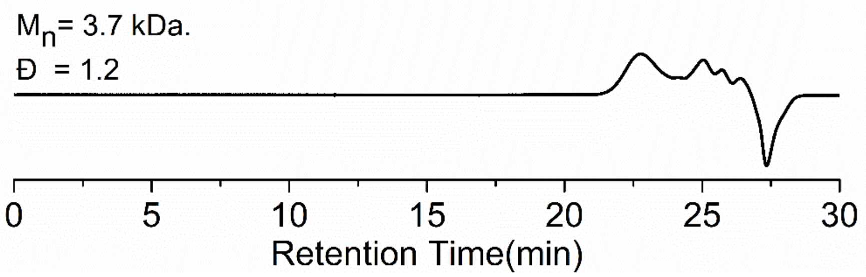

Fig S52. GPC (THF) trace of the crude polymer **P8.**

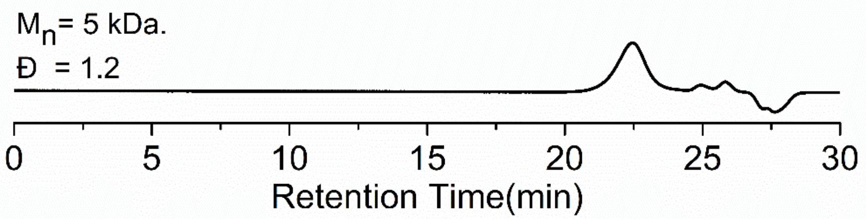

Fig S53. GPC (THF) trace of the crude polymer **P9.**

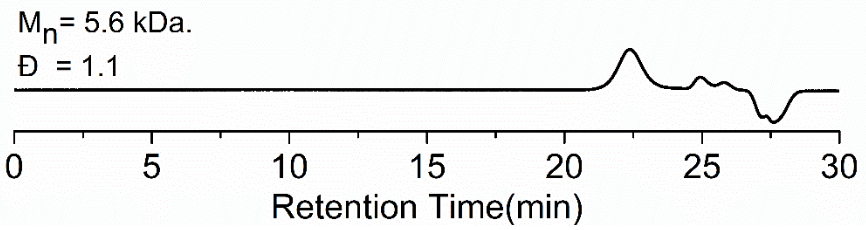

Fig S54. GPC (THF) trace of the crude polymer **P10.**

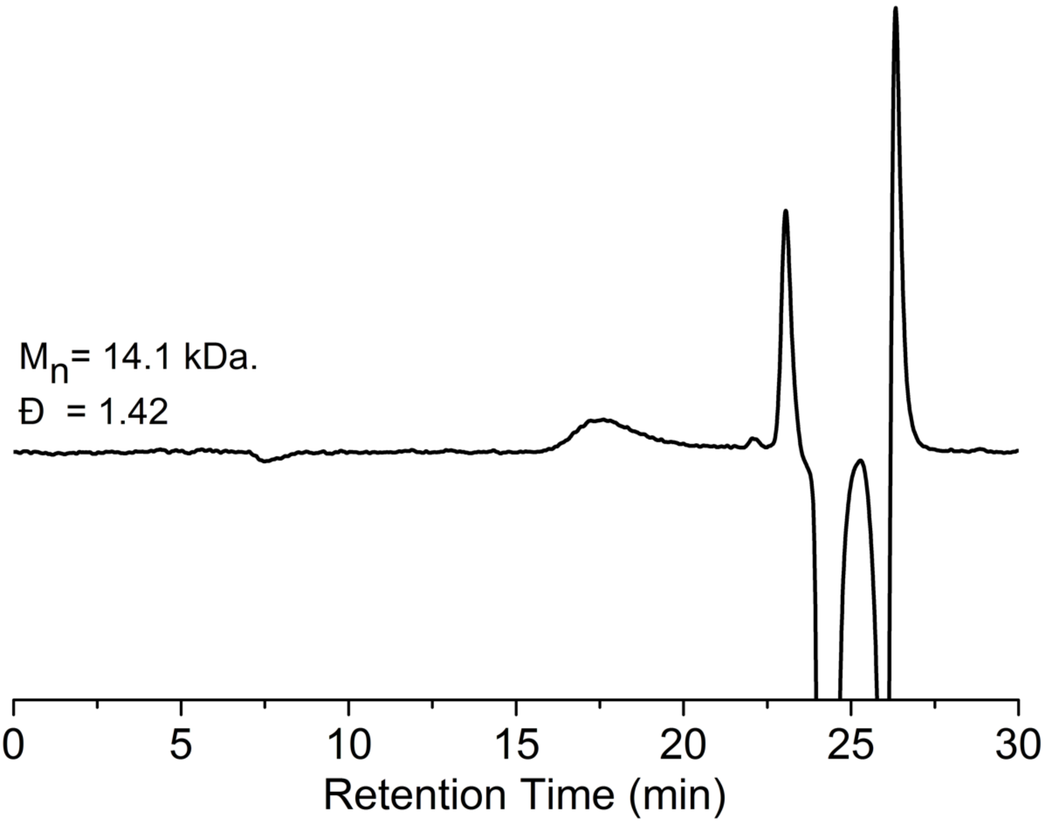

Fig S55. GPC ($CHCl_3$) trace of the crude polymer **P11.**

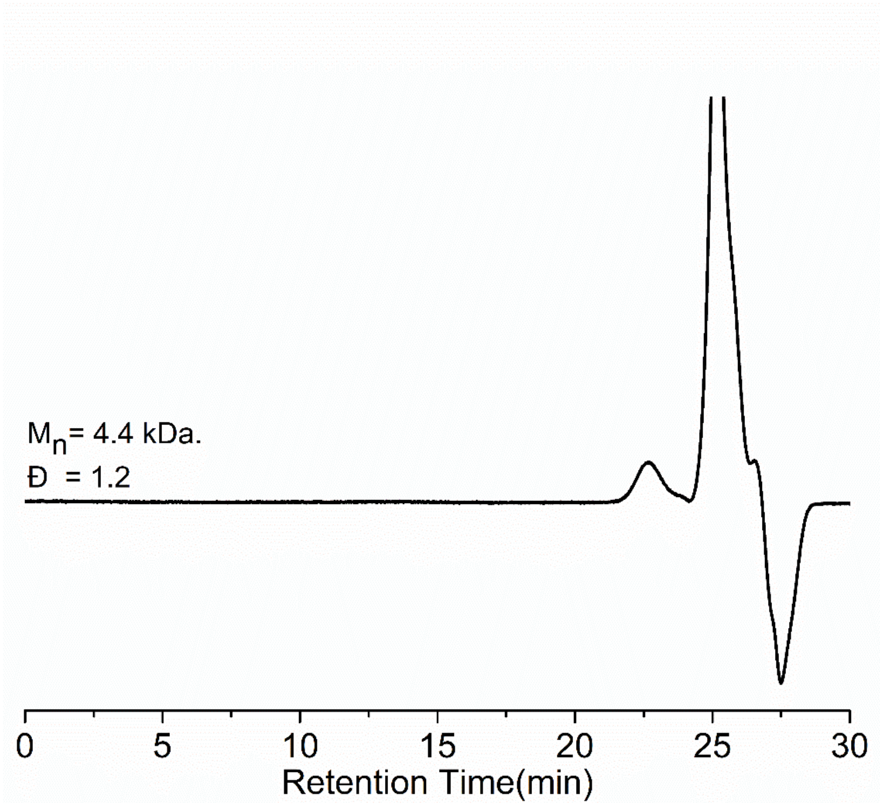

Fig S56. GPC (THF) trace of the crude polymer **P12.**

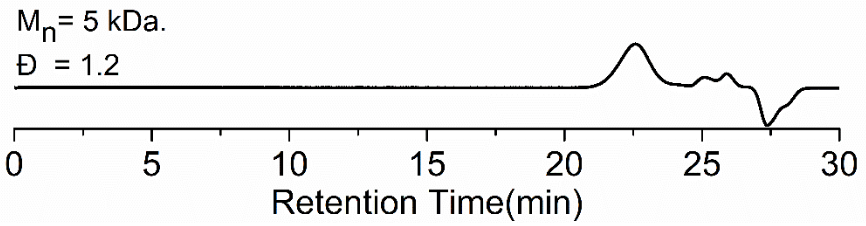

Fig S57. GPC (THF) trace of the crude polymer **P13.**

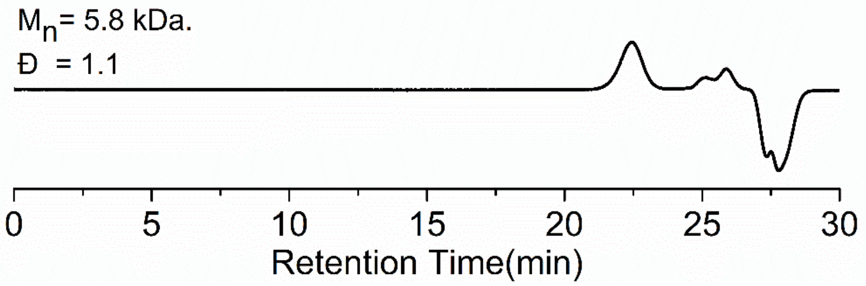

Fig S58. GPC (THF) trace of the crude polymer **P14.**

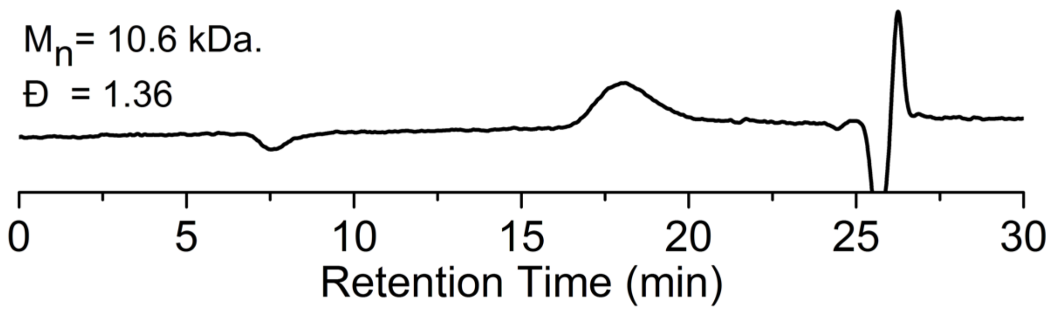

Fig S59. GPC ($CHCl_3$) trace of the crude polymer **P15.**

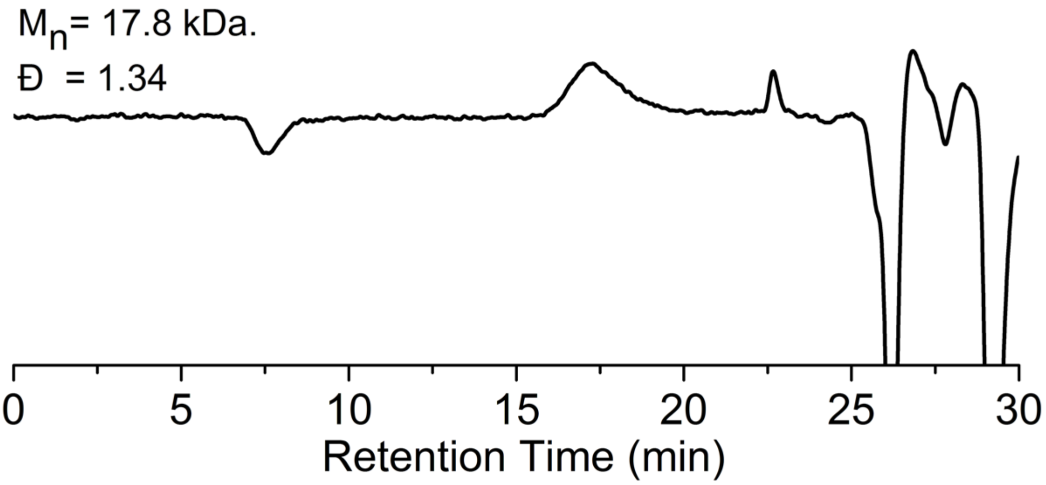

Fig S60. GPC ($CHCl_3$) trace of the crude polymer **P16.**

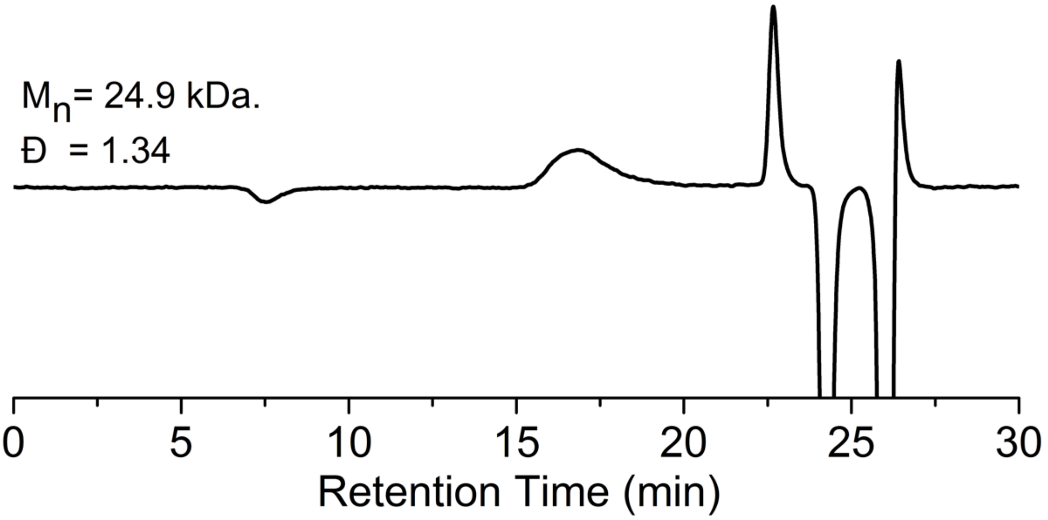

Fig S61. GPC ($CHCl_3$) trace of the crude polymer **P17.**

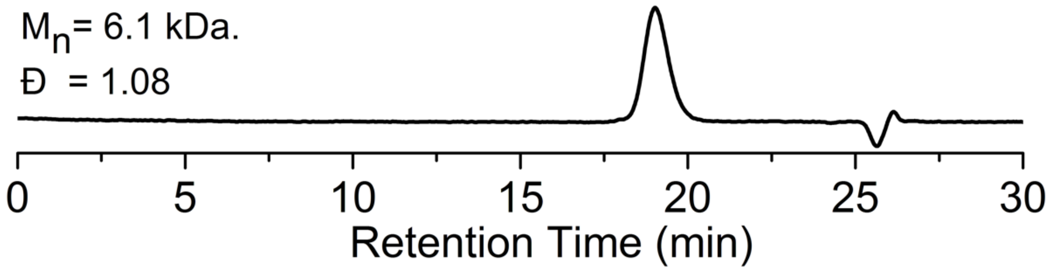

Fig S62. GPC ($CHCl_3$) trace of the crude polymer **P18.**

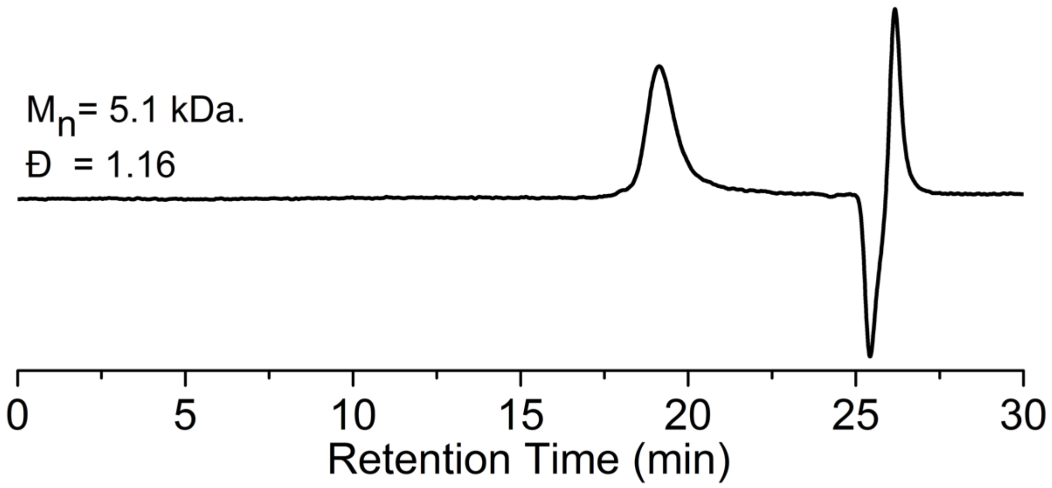

Fig S63. GPC ($CHCl_3$) trace of the crude polymer **P19.**

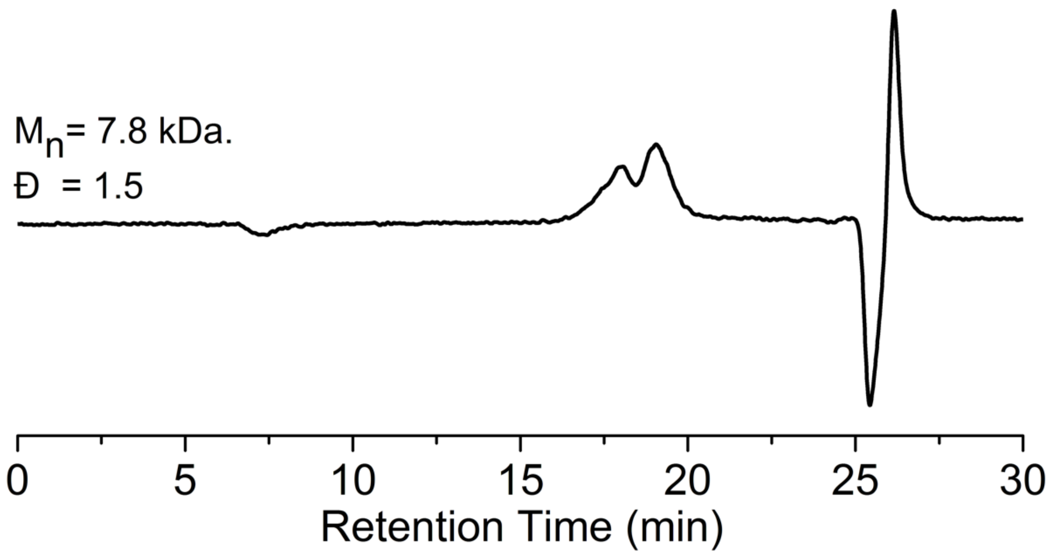

Fig S64. GPC ($CHCl_3$) trace of the crude polymer **P20.** We believe formation of disulfide is the origin of bimodal distribution.

MALDI-ToF mass spectra of polymers

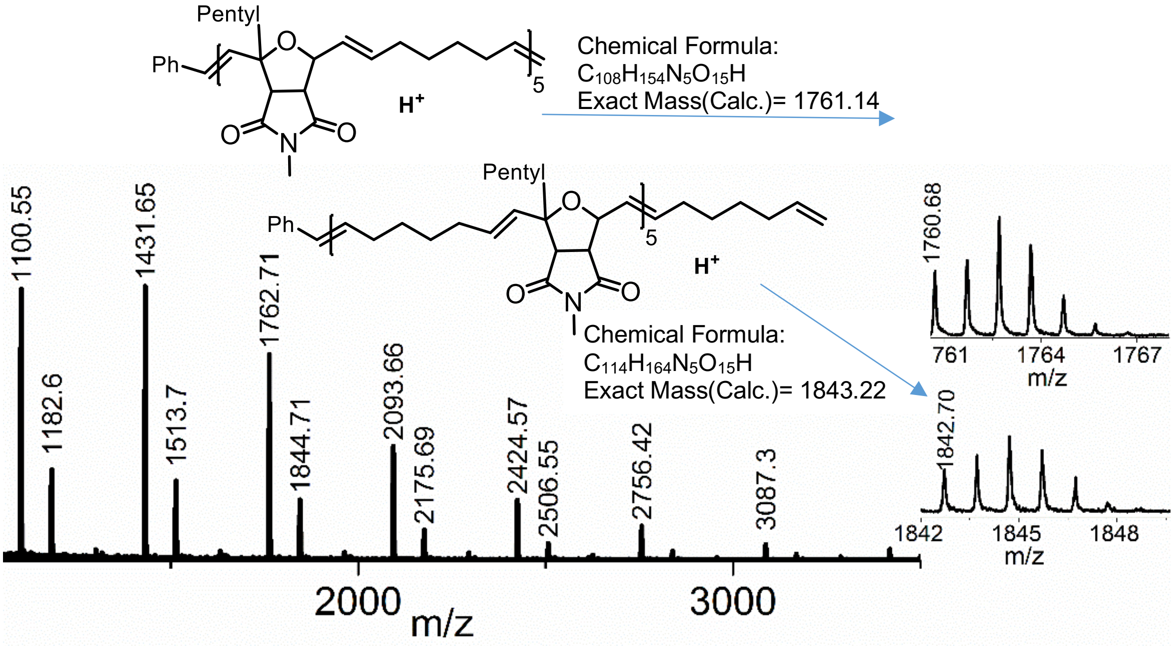

Fig S65. MALDI-ToF mass spectrum (DCTB) of polymer **P14**.

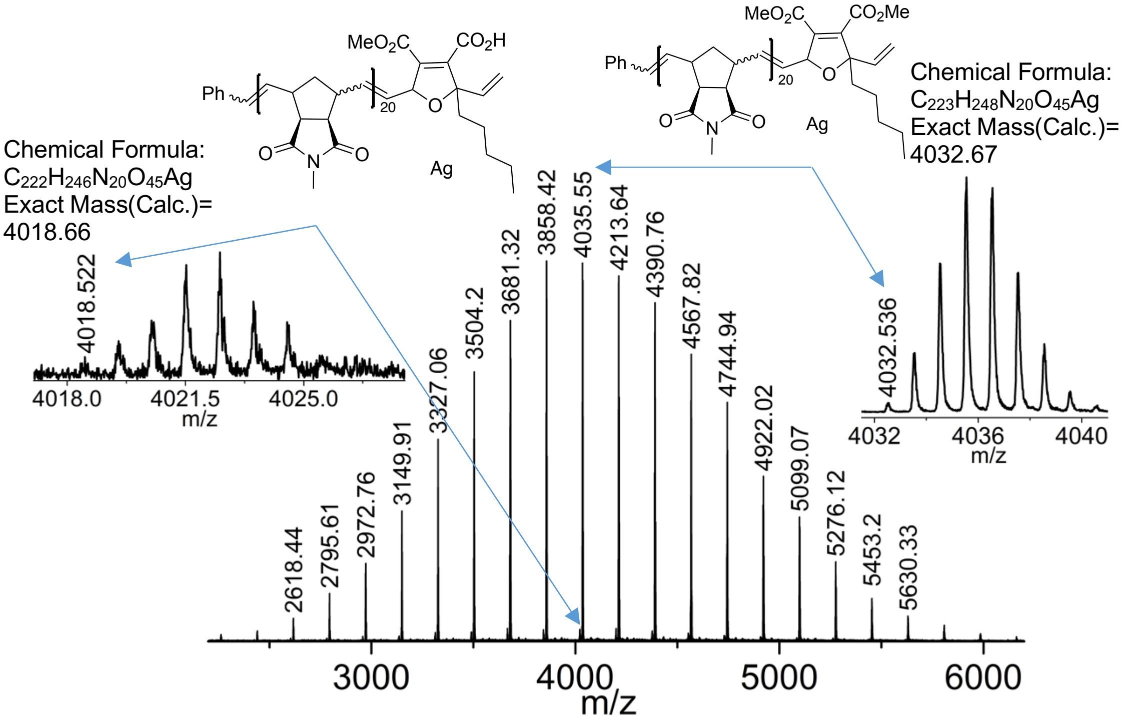

Fig S66. MALDI-ToF mass spectrum (DCTB, AgTFA) of polymer **P18**. The smaller distribution most likely generated under ionization conditions in the MALDI-ToF mass spectrometer.

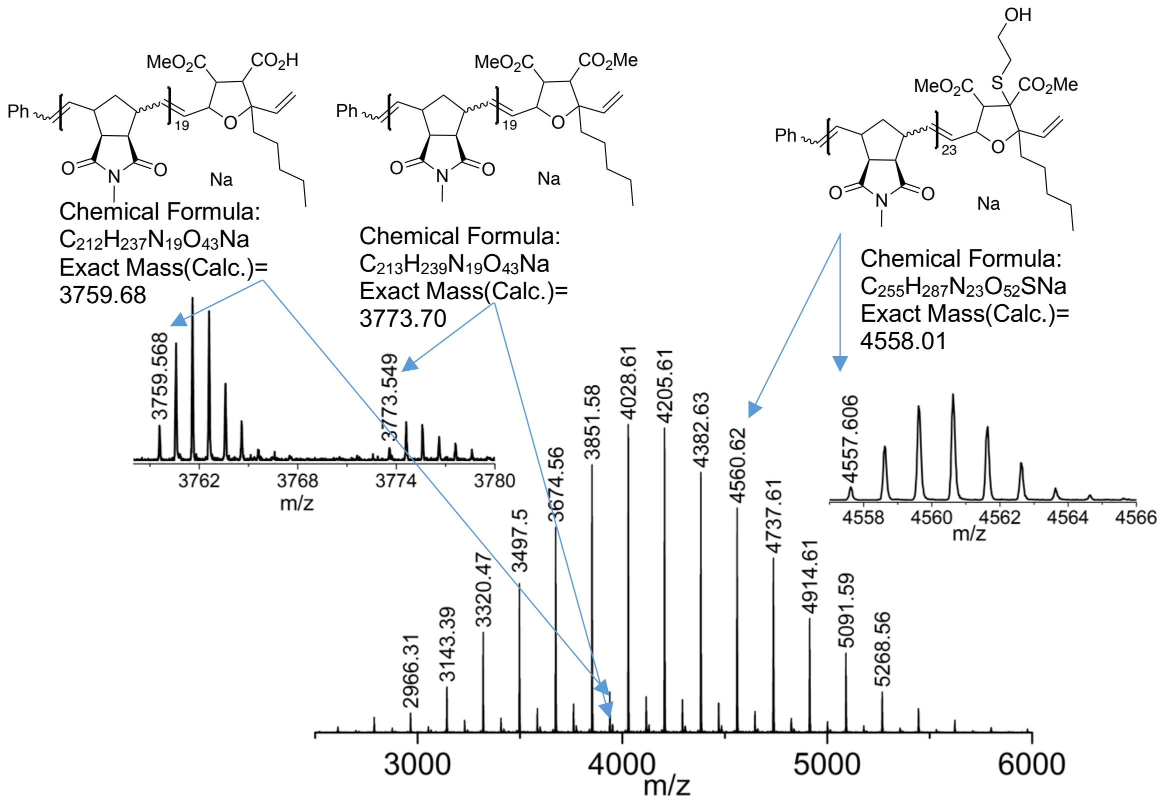

Fig S67. MALDI-ToF mass spectrum (DCTB, NaTFA) of polymer **P19**. The same smaller distribution appeared in both cases, most likely generated under ionization conditions in the MALDI-ToF mass spectrometer.

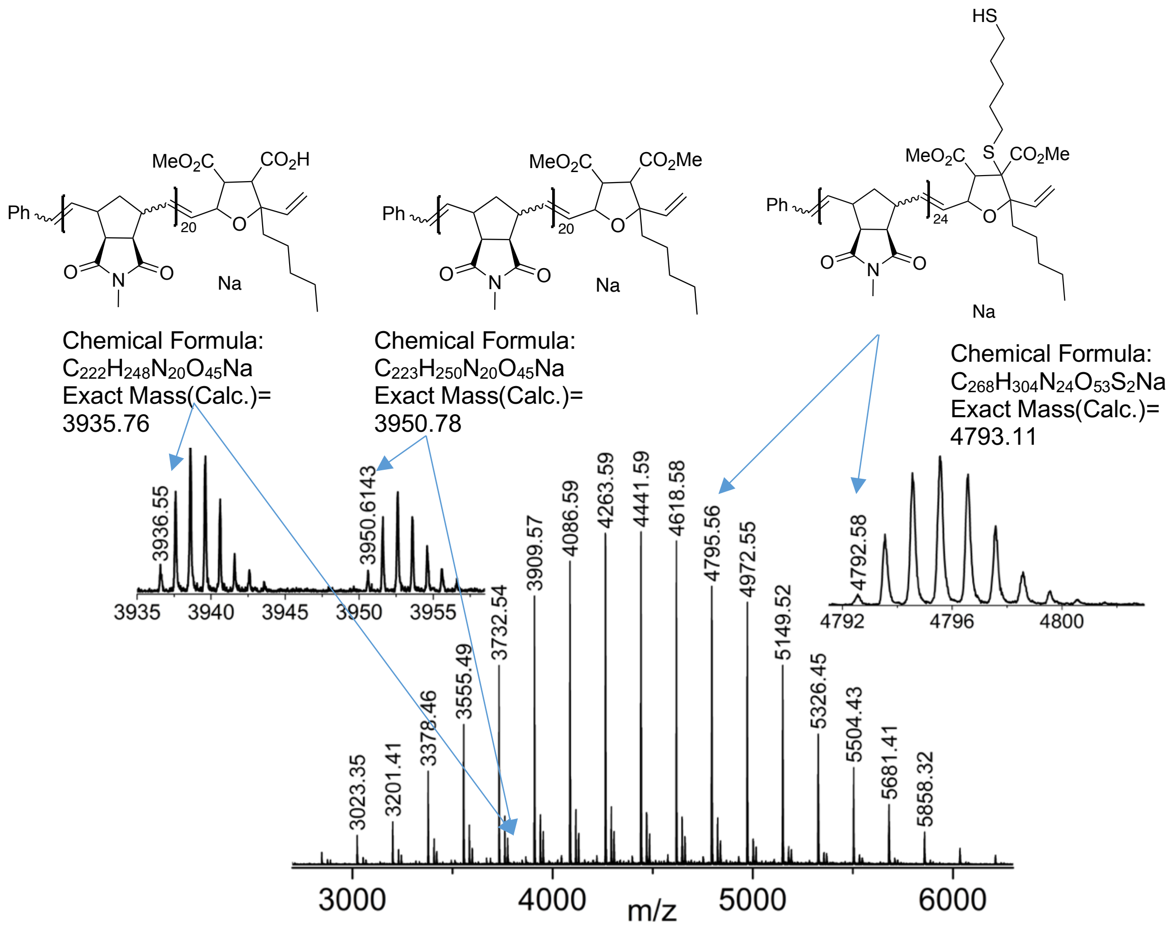

Fig S68. MALDI-ToF mass spectrum (DCTB, NaTFA) of polymer **P20**. The same smaller distribution appeared in both cases, most likely generated under ionization conditions in the MALDI-ToF mass spectrometer.

NMR spectra of polymers

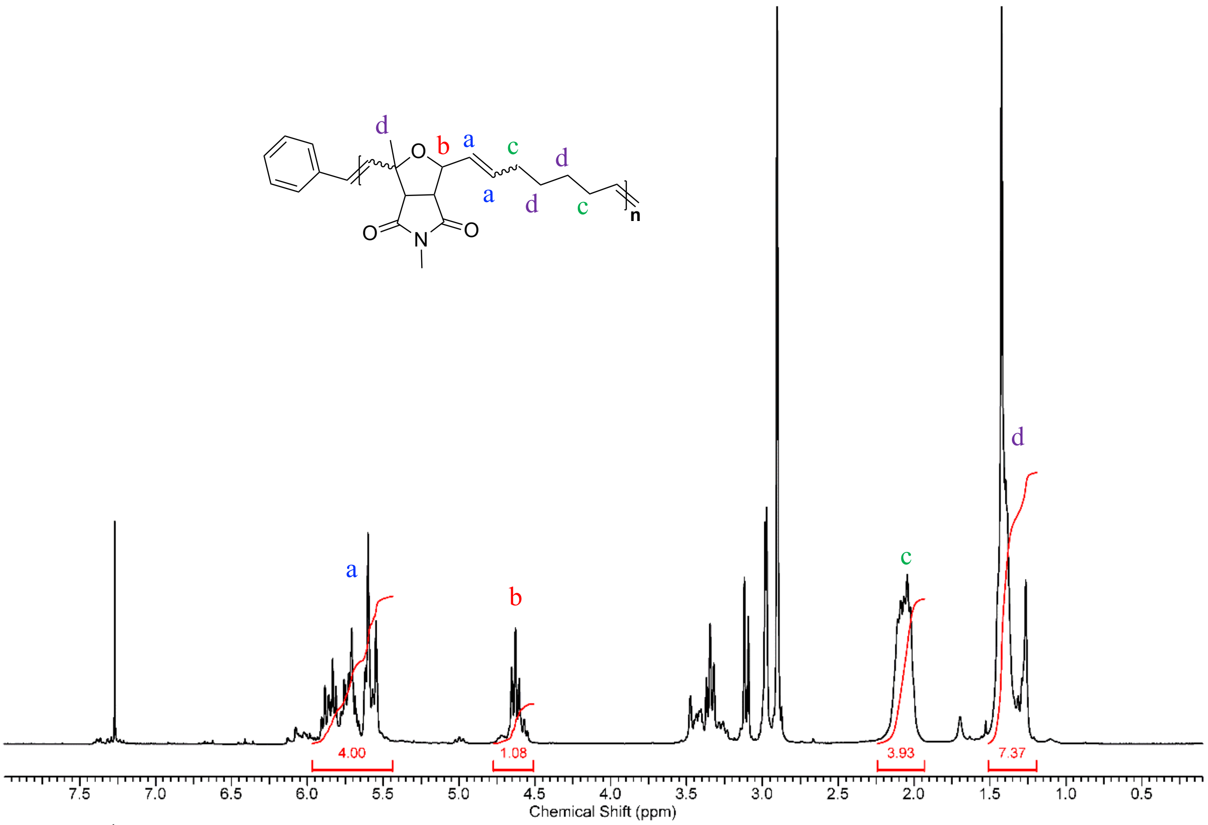

Fig S69. ^{1}H NMR (chloroform-d, 300 MHz) spectrum of Poly **P1.**

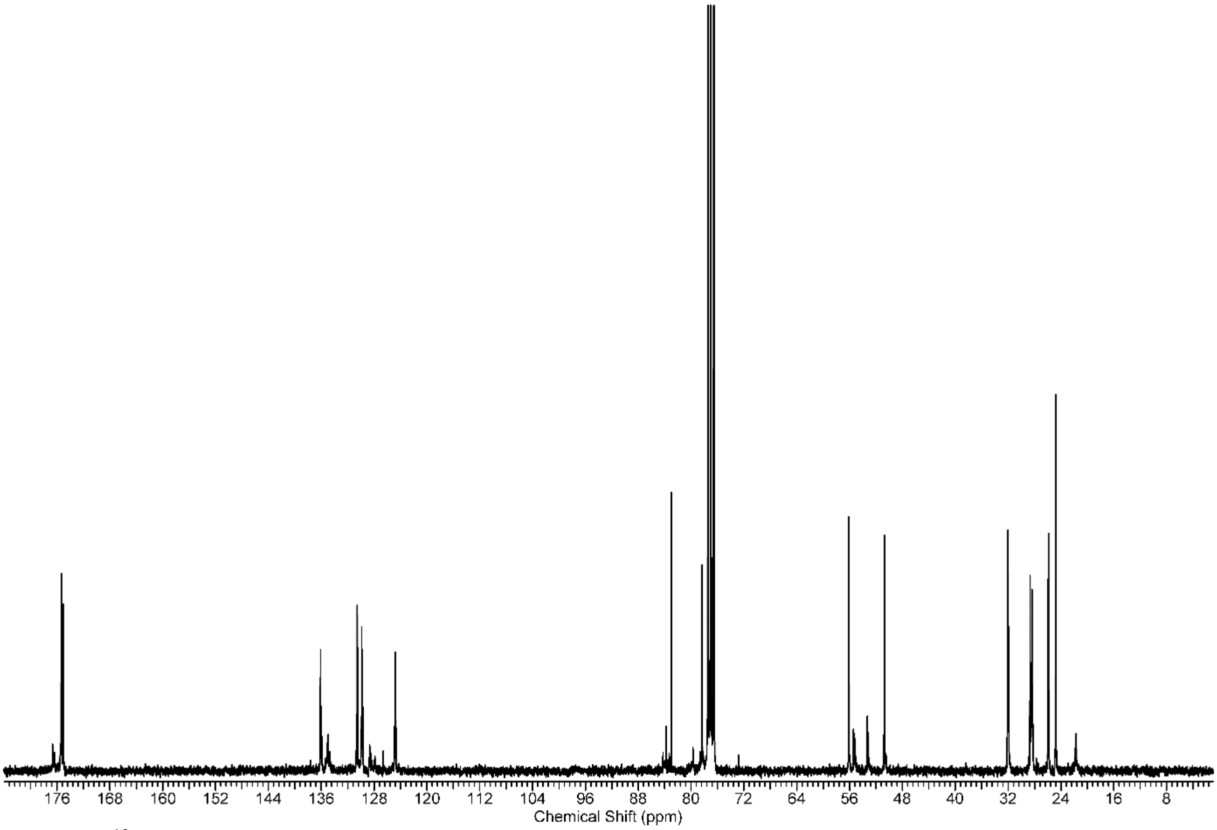

Fig S70. ^{13}C NMR (chloroform-d, 75 MHz) spectrum of Poly **P1.**

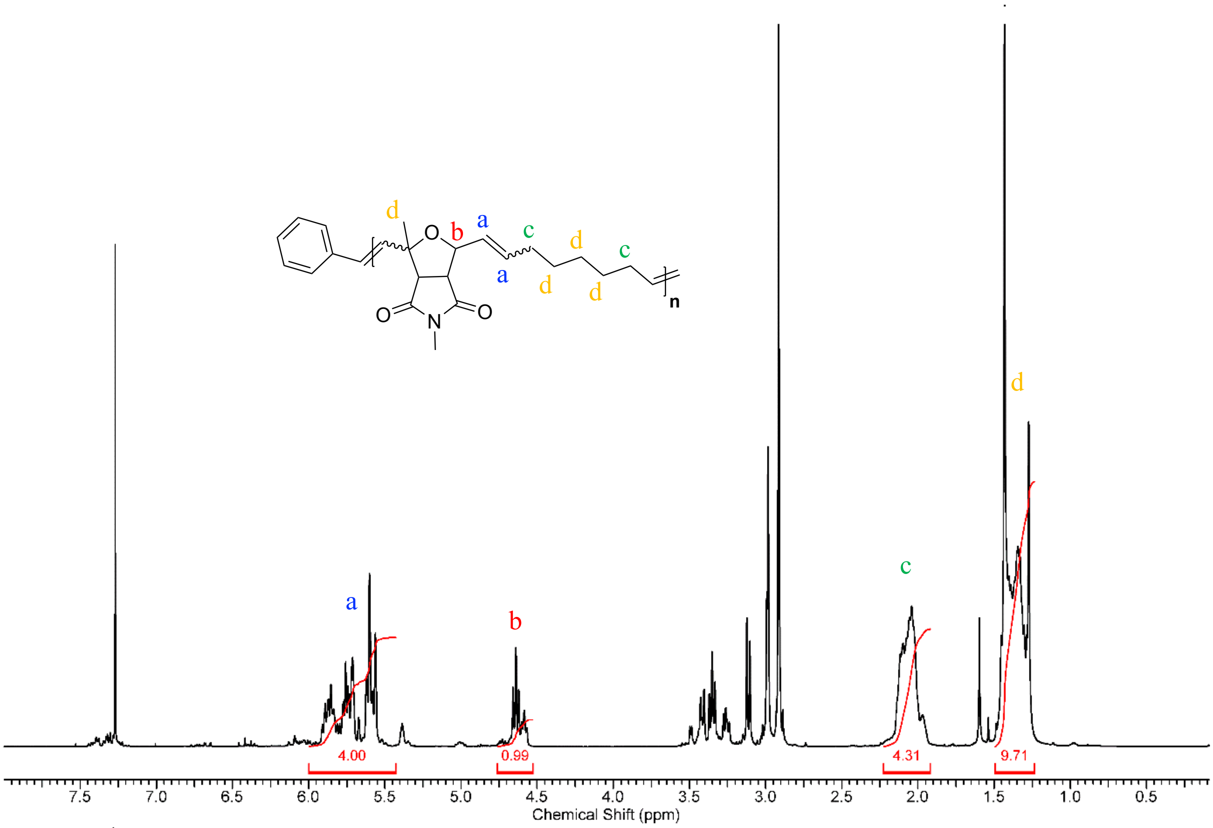

Fig S71. ^{1}H NMR (chloroform-d, 400 MHz) spectrum of Poly **P3.**

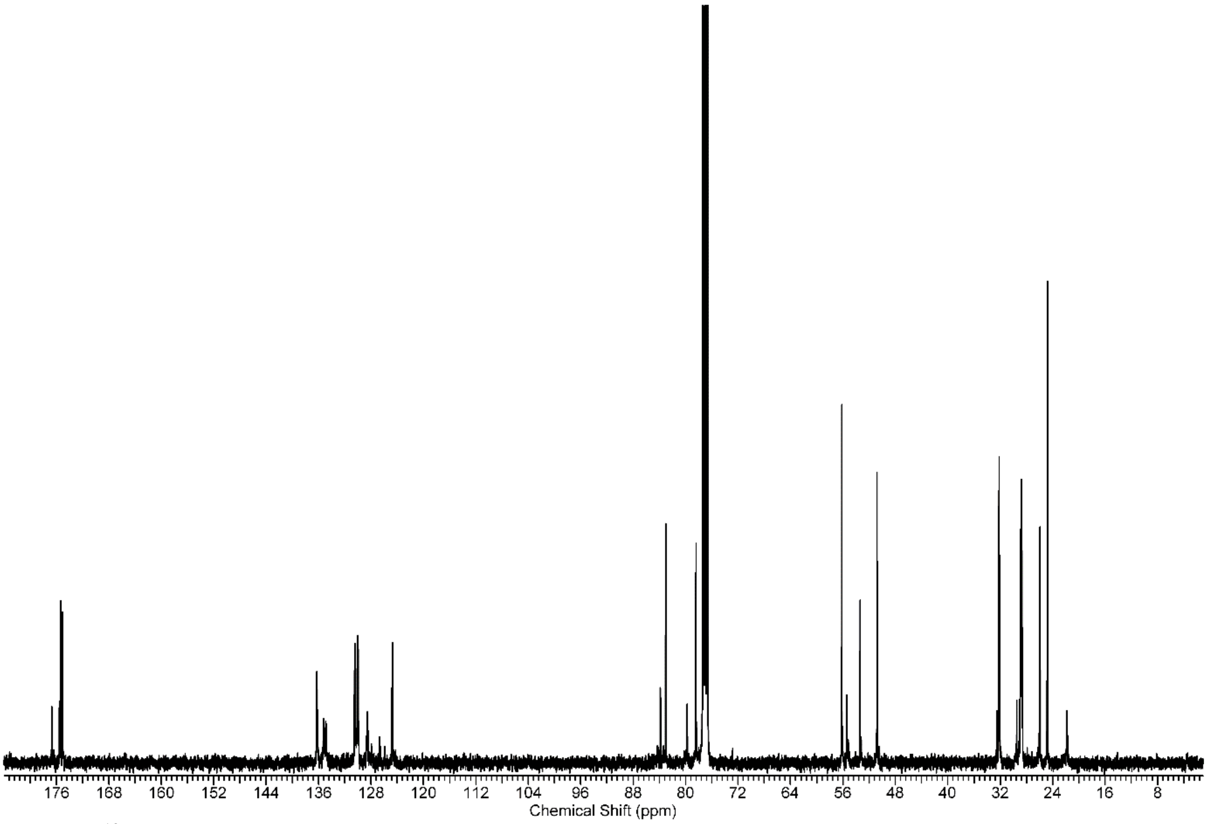

Fig S72. ^{13}C NMR (chloroform-d, 101 MHz) spectrum of Poly **P3.**

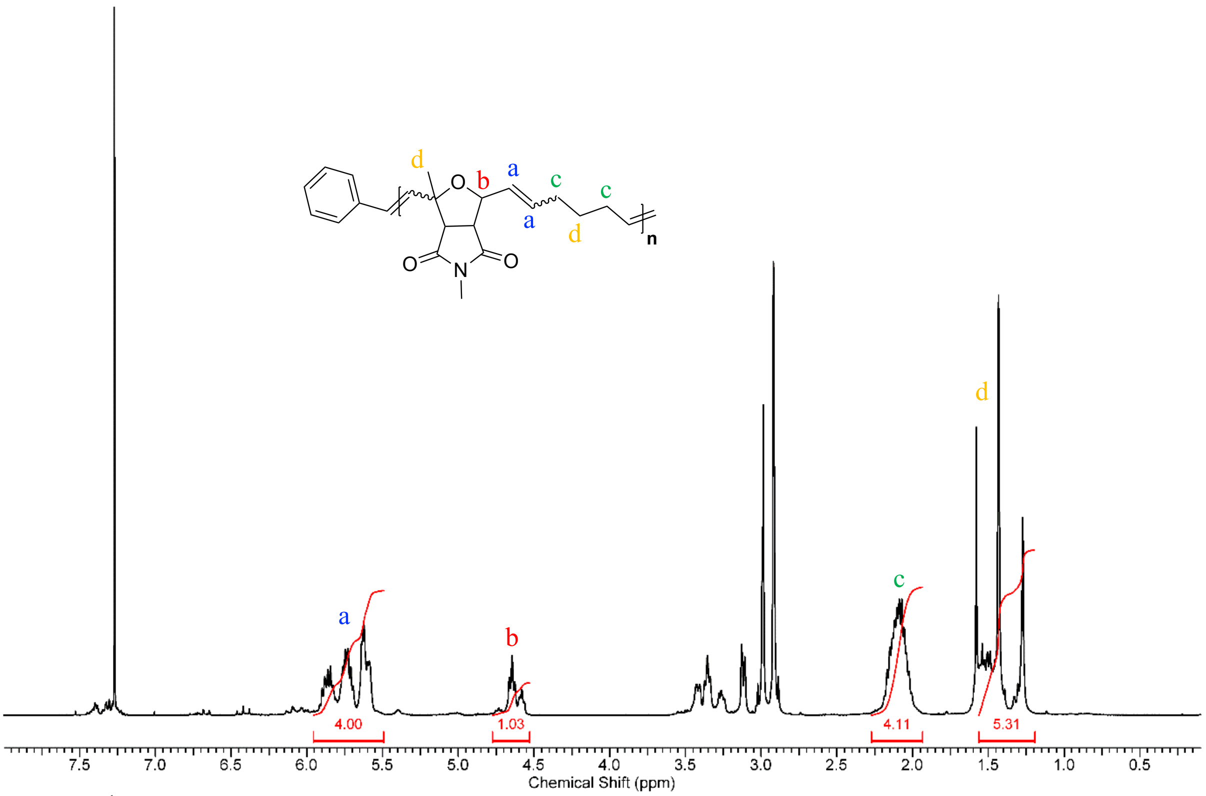

Fig S73. ^{1}H NMR (chloroform-d, 400 MHz) spectrum of Poly **P5.**

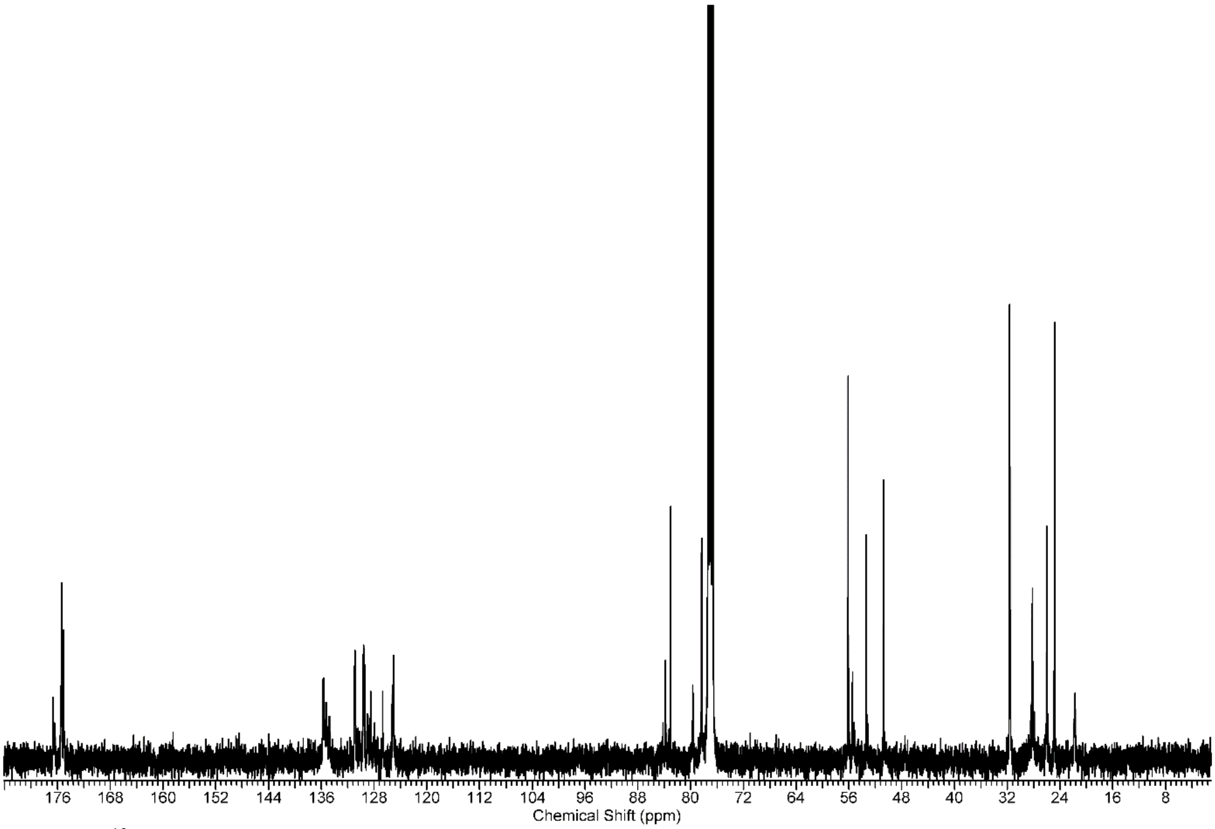

Fig S74. ^{13}C NMR (chloroform-d, 101 MHz) spectrum of Poly **P5.**

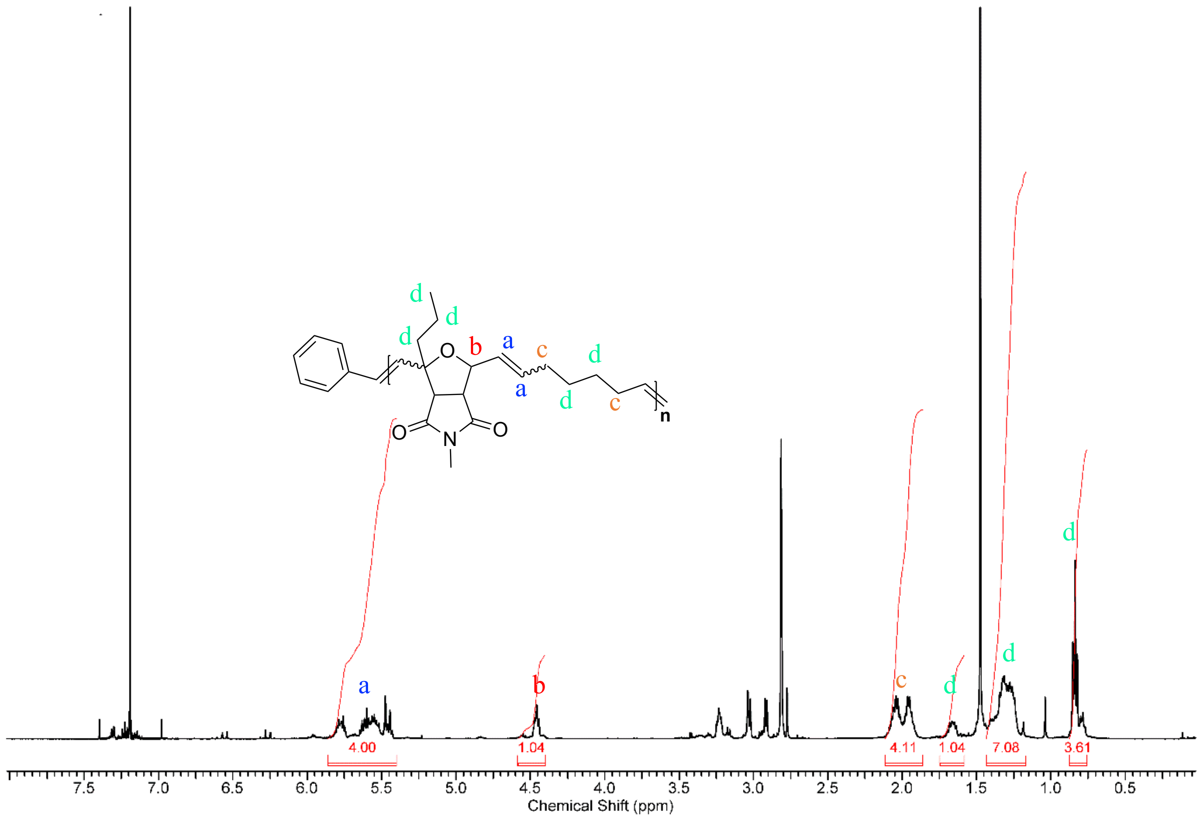

Fig S75. ¹H NMR (chloroform-d, 500 MHz) spectrum of Poly **P6.**

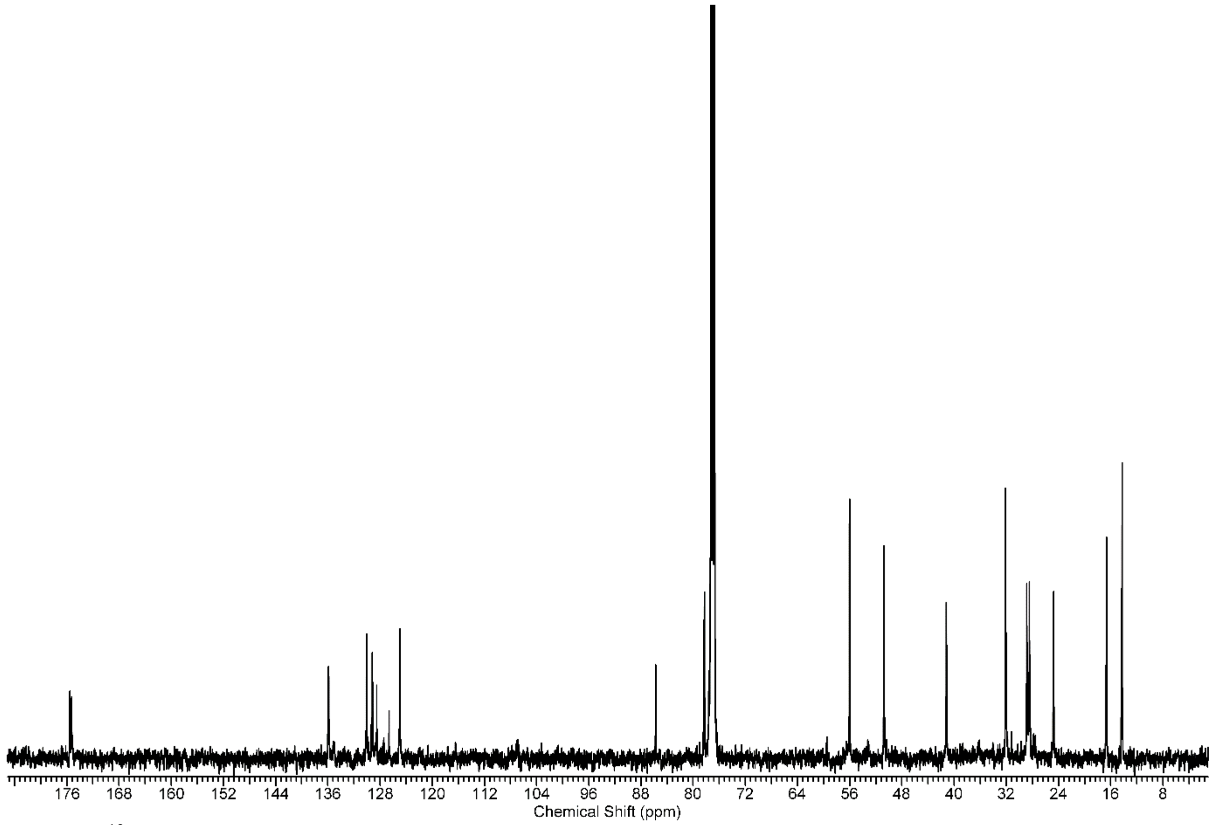

Fig S76. ¹³C NMR (chloroform-d, 125 MHz) spectrum of Poly **P6.**

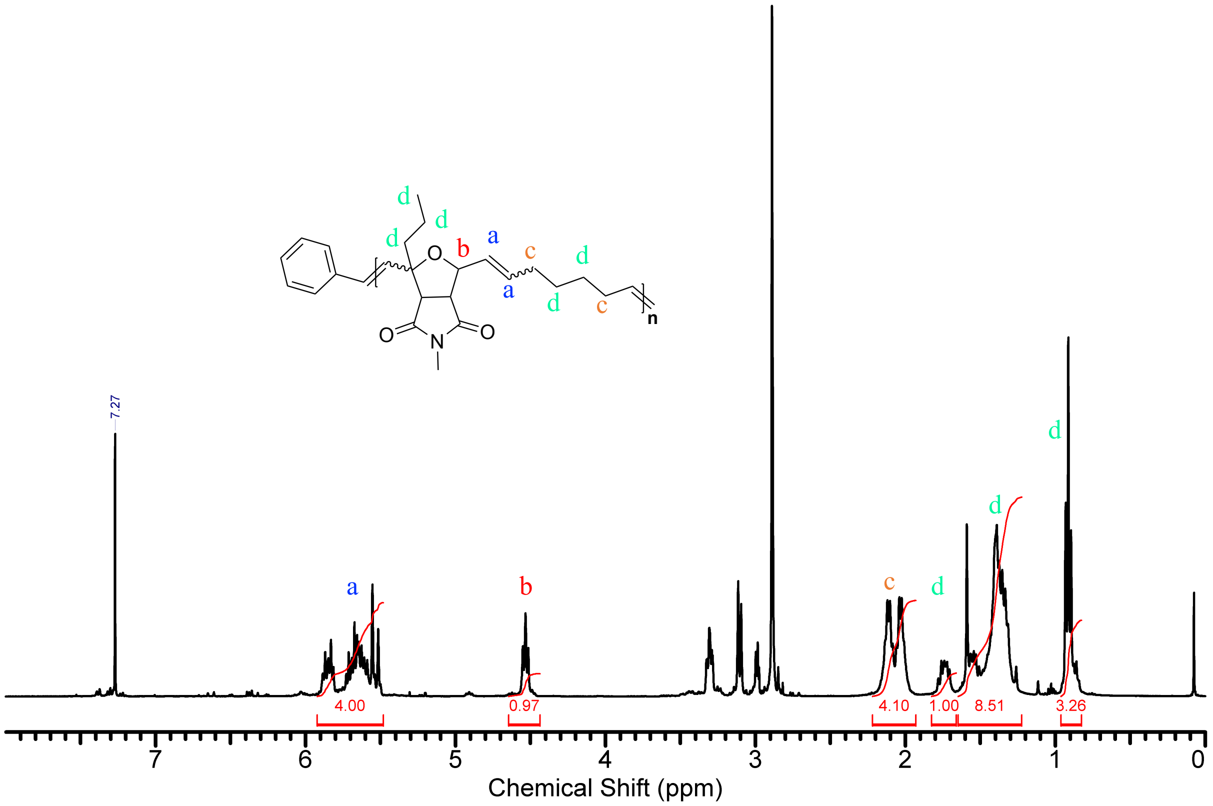

Fig S77. ^{1}H NMR (chloroform-d, 400 MHz) spectrum of Poly **P7.**

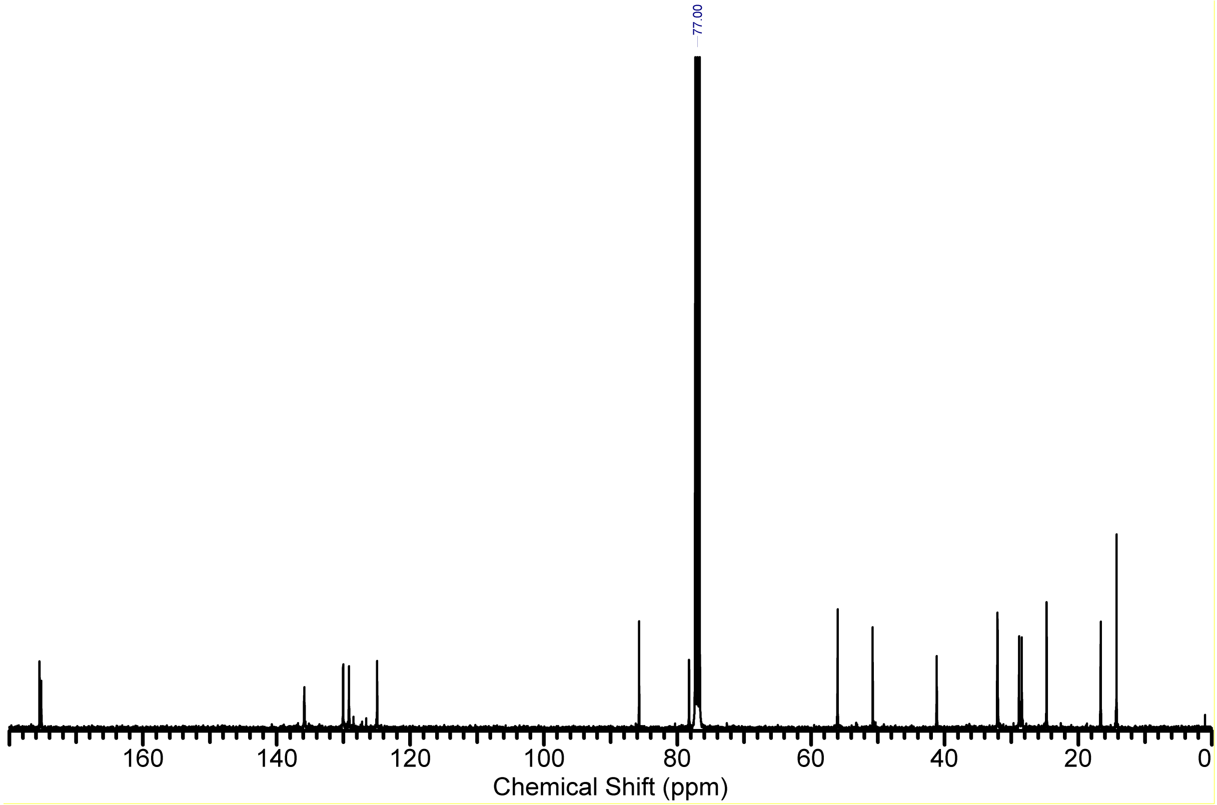

Fig S78. ^{13}C NMR (chloroform-d, 101 MHz) spectrum of Poly **P7.**

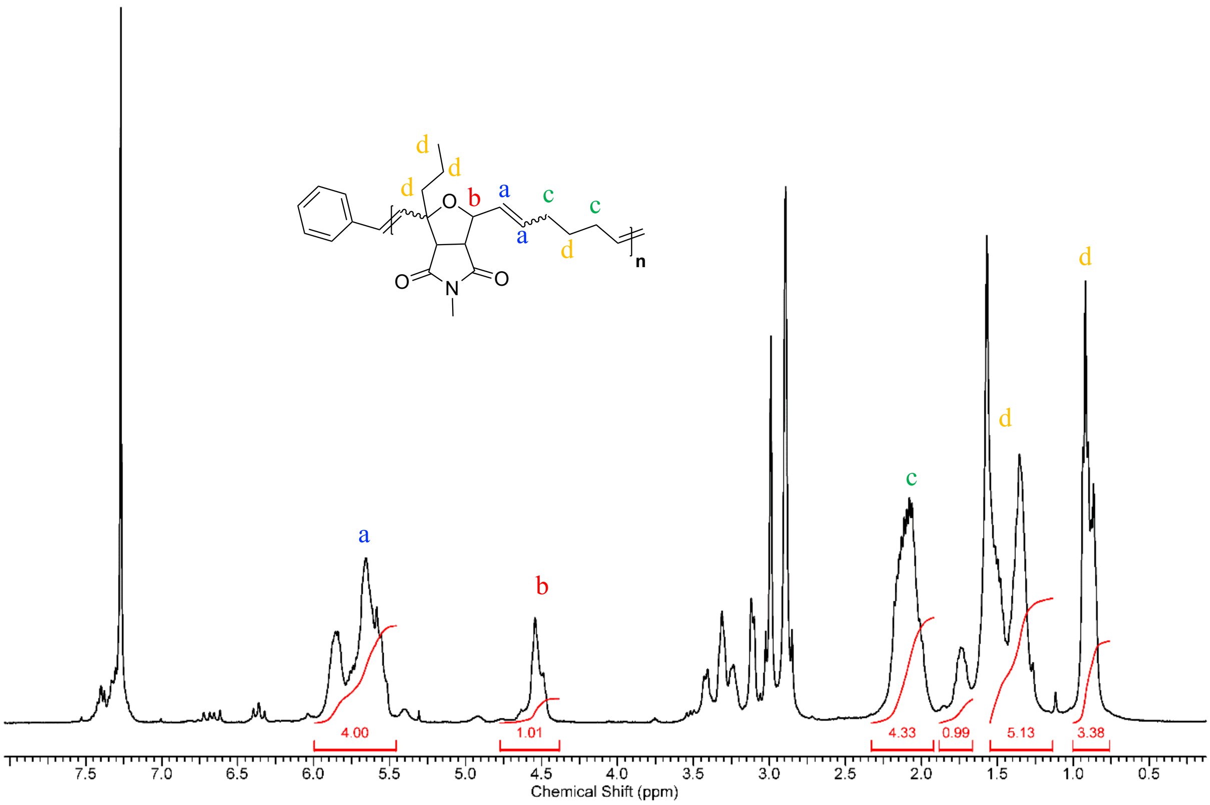

Fig S79. ^{1}H NMR (chloroform-d, 400 MHz) spectrum of Poly **P8.**

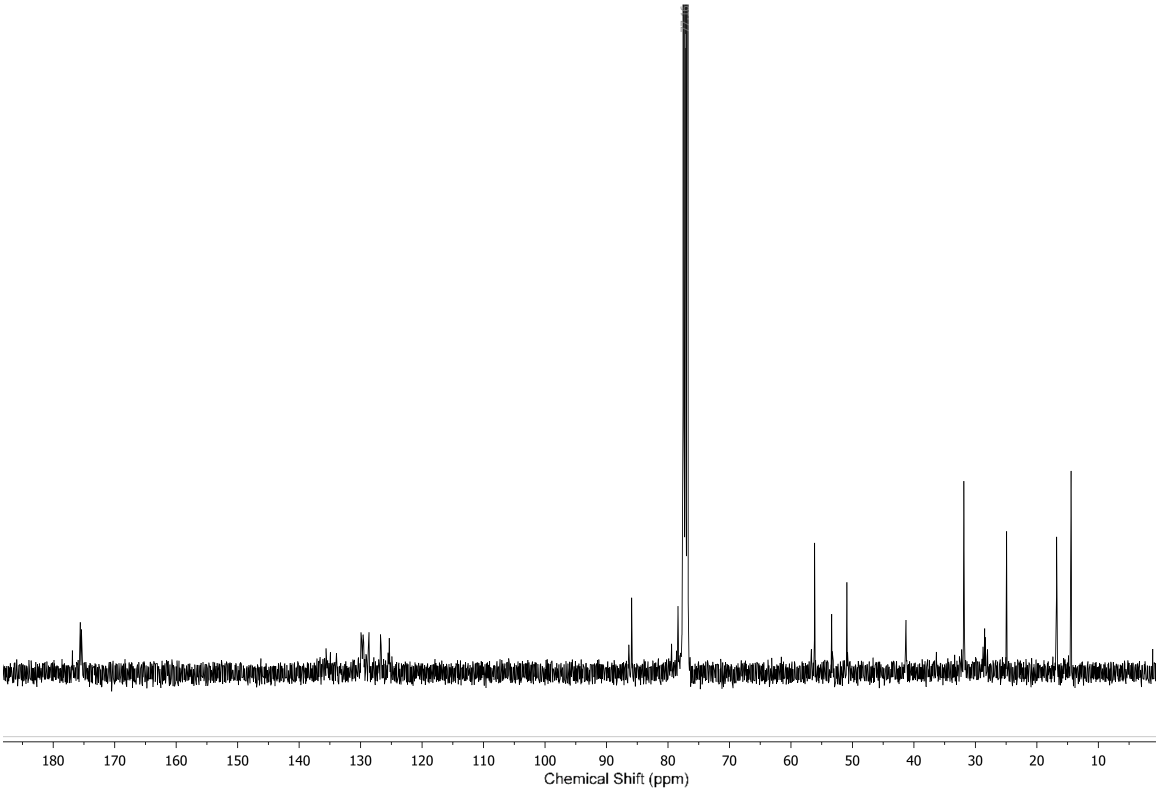

Fig S80. ^{13}C NMR (chloroform-d, 101 MHz) spectrum of Poly **P8.**

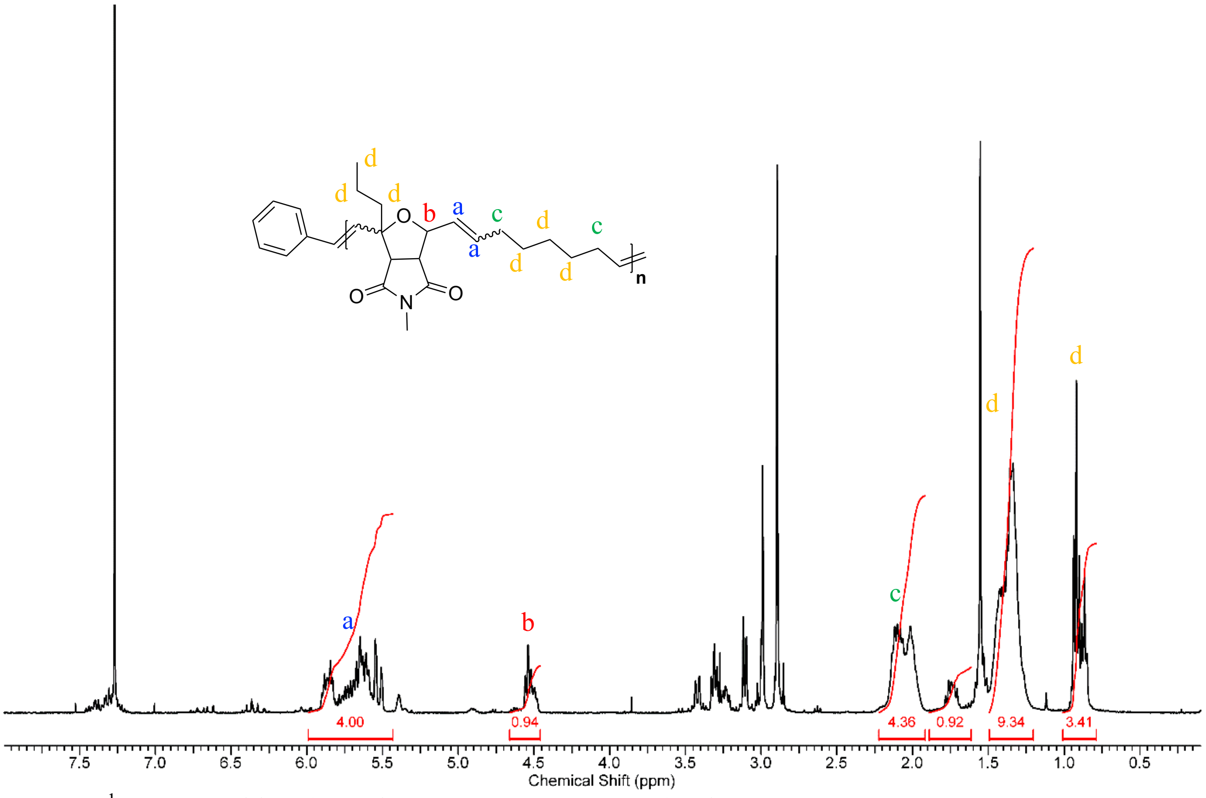

Fig S81. ^{1}H NMR (chloroform-d, 400 MHz) spectrum of Poly **P9.**

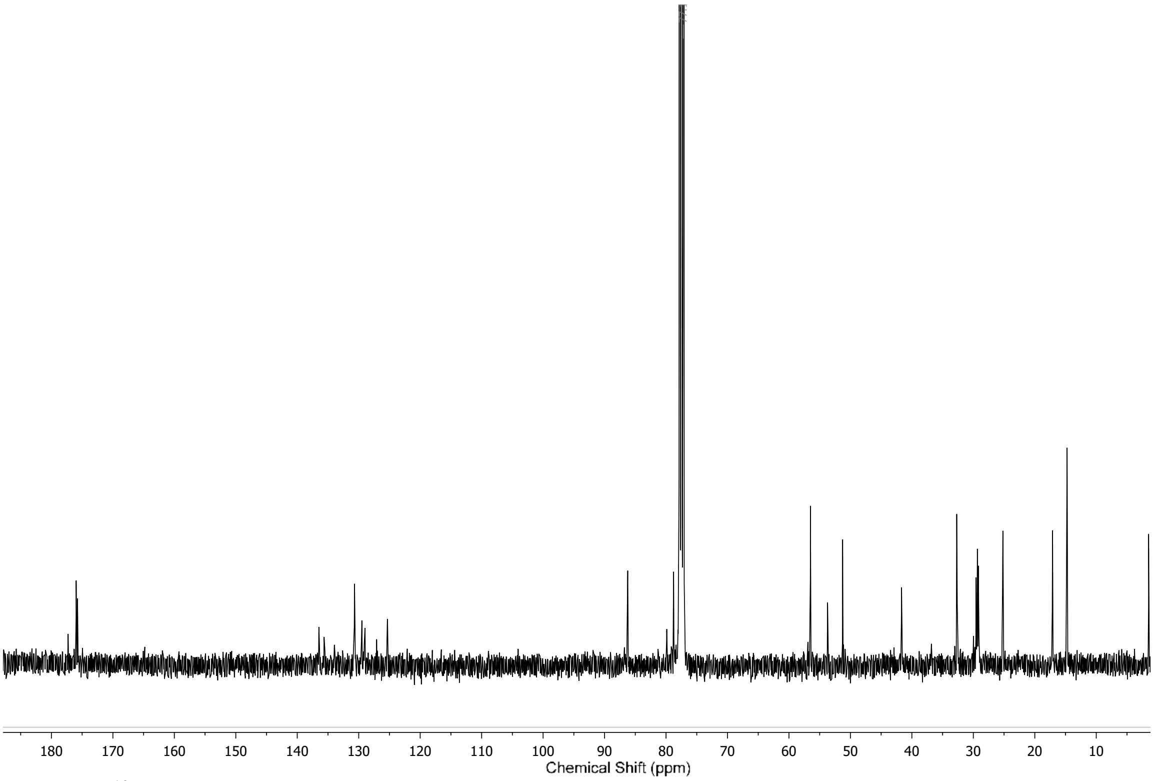

Fig S82. ^{13}C NMR (chloroform-d, 101 MHz) spectrum of Poly **P9.**

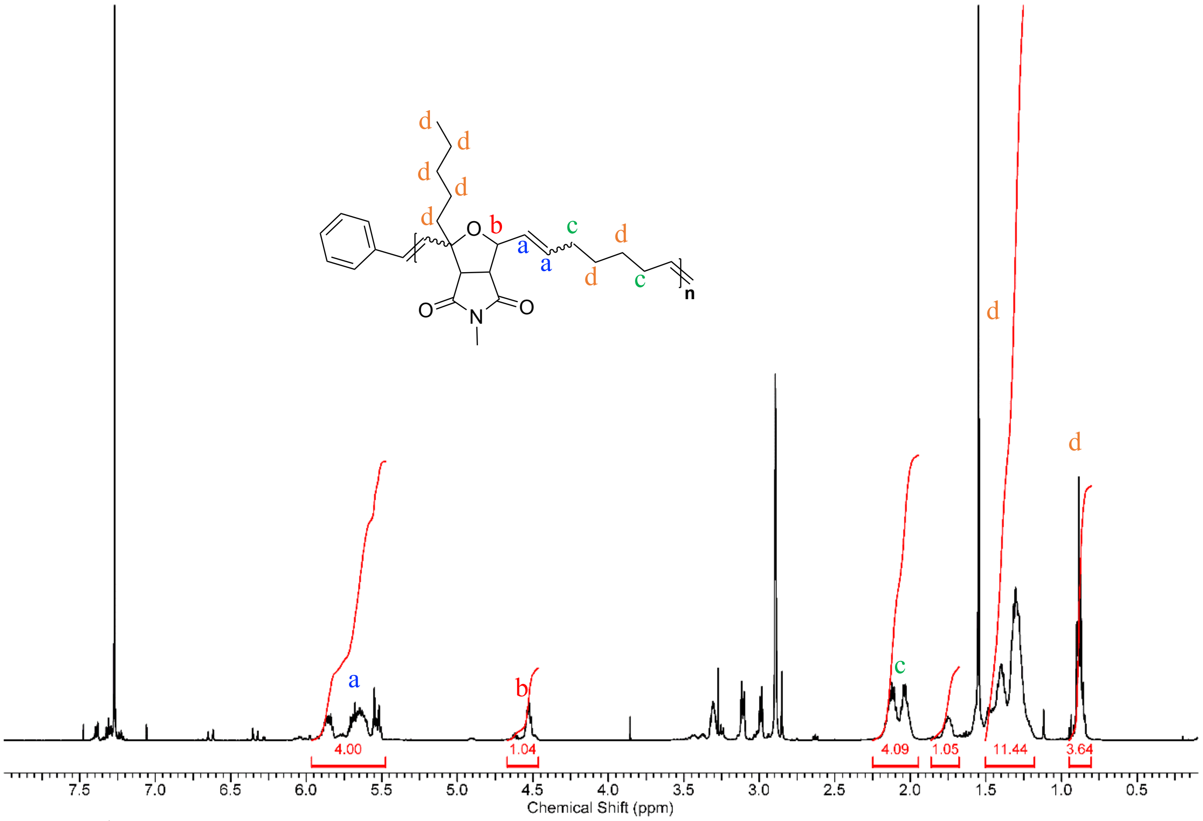

Fig S83. ^{1}H NMR (chloroform-d, 500 MHz) spectrum of Poly **P10.**

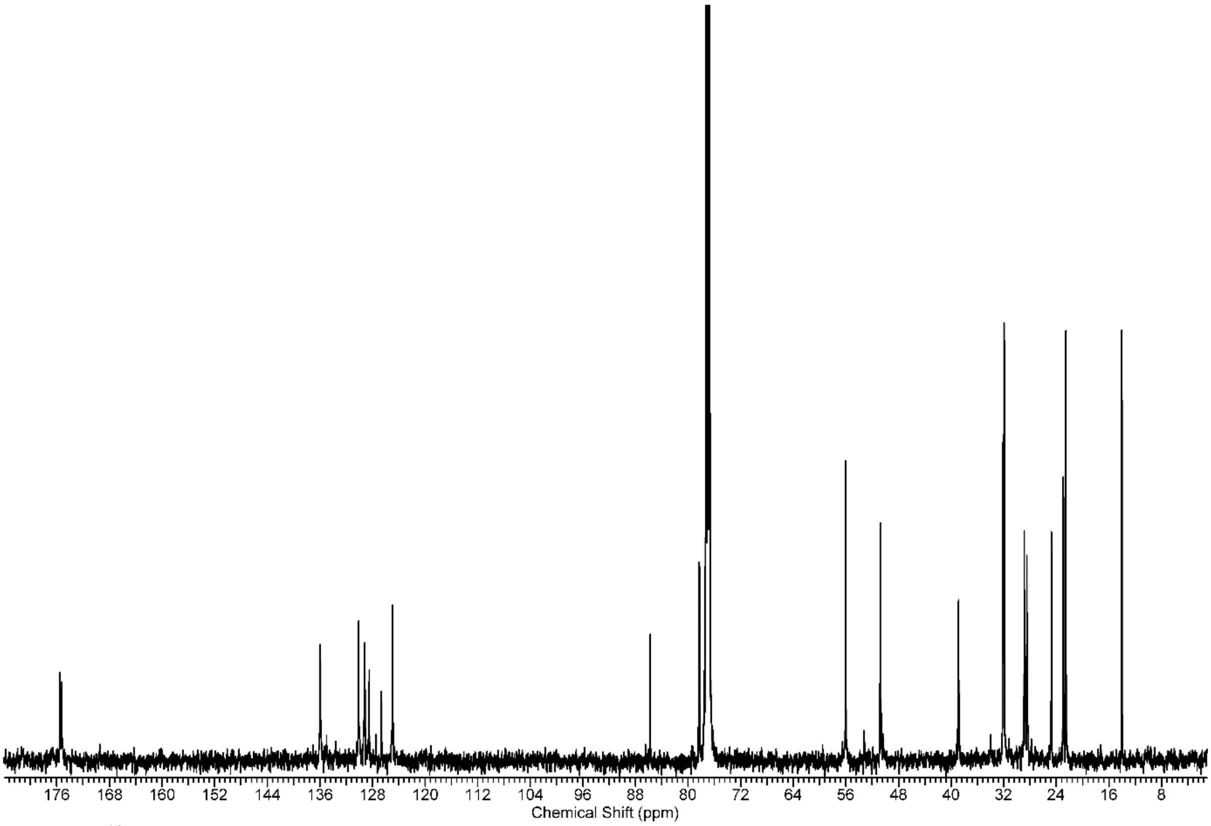

Fig S84. ^{13}C NMR (chloroform-d, 125 MHz) spectrum of Poly **P10.**

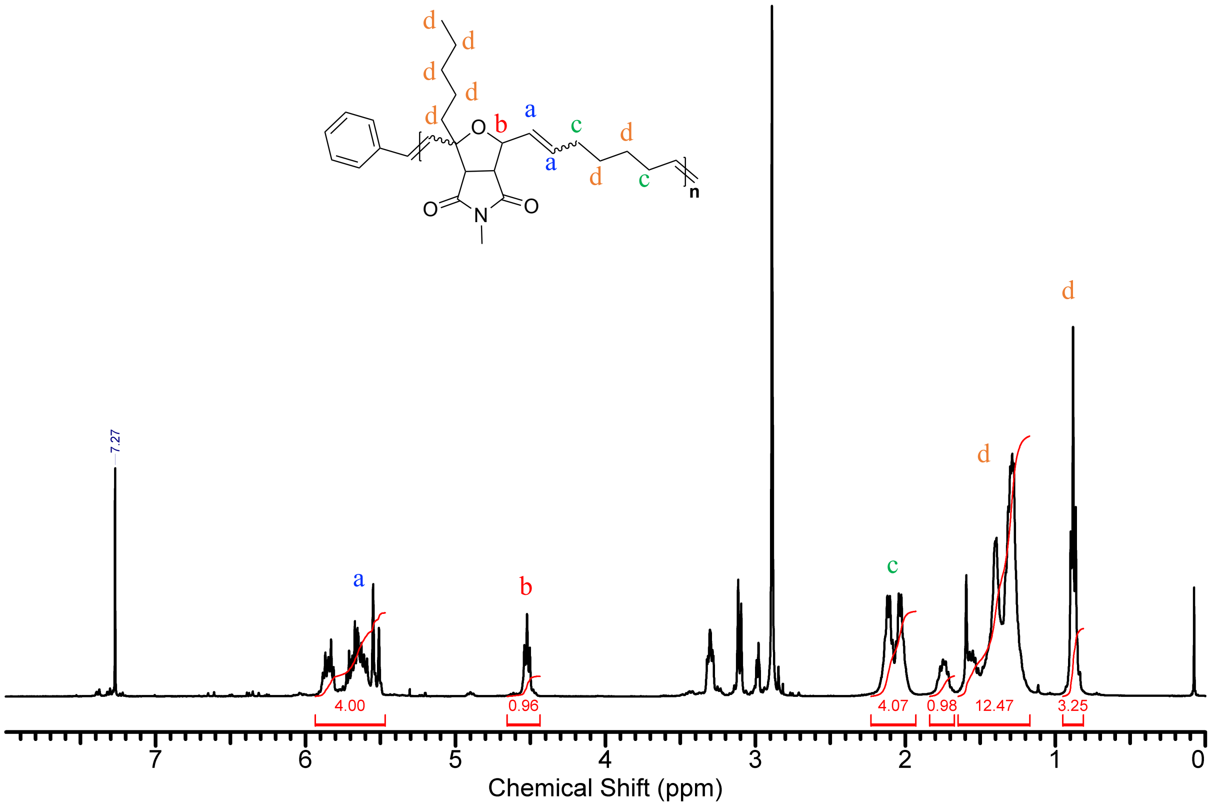

Fig S85. ^{1}H NMR (chloroform-d, 400 MHz) spectrum of Poly **P11.**

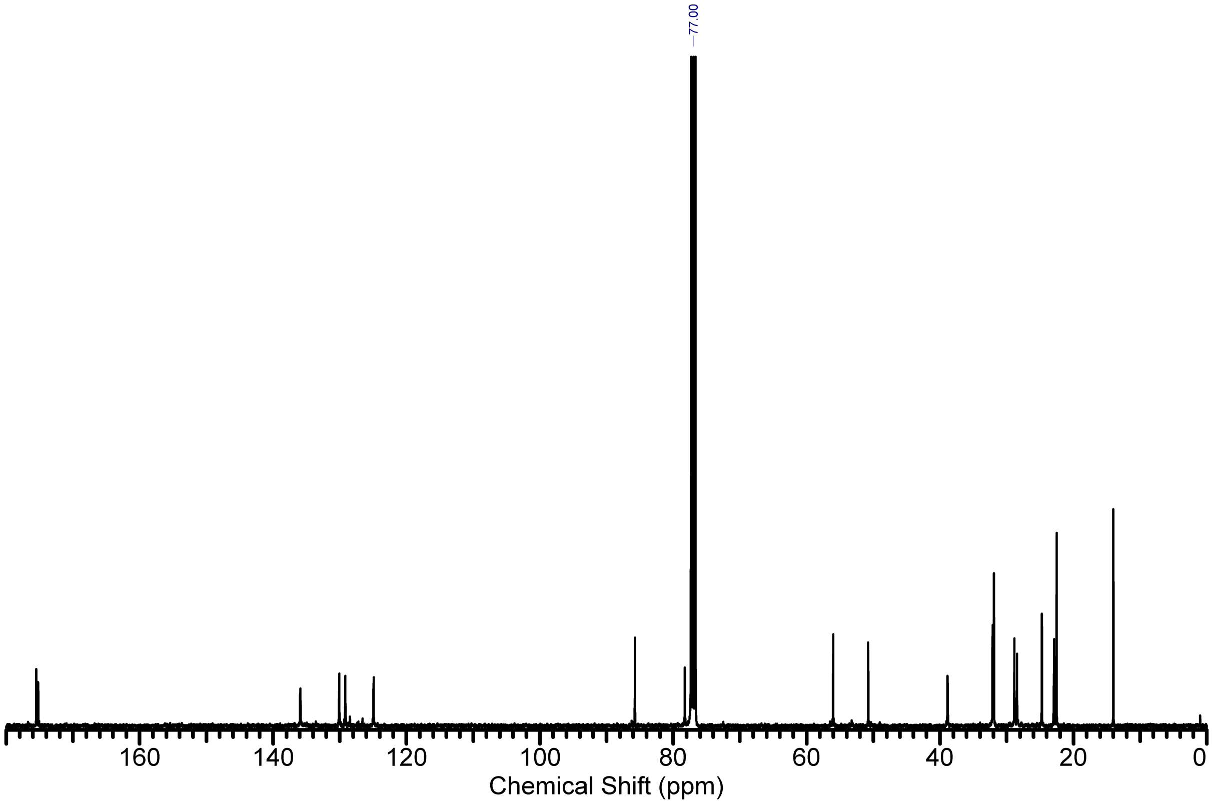

Fig S86. ^{13}C NMR (chloroform-d, 101 MHz) spectrum of Poly **P11.**

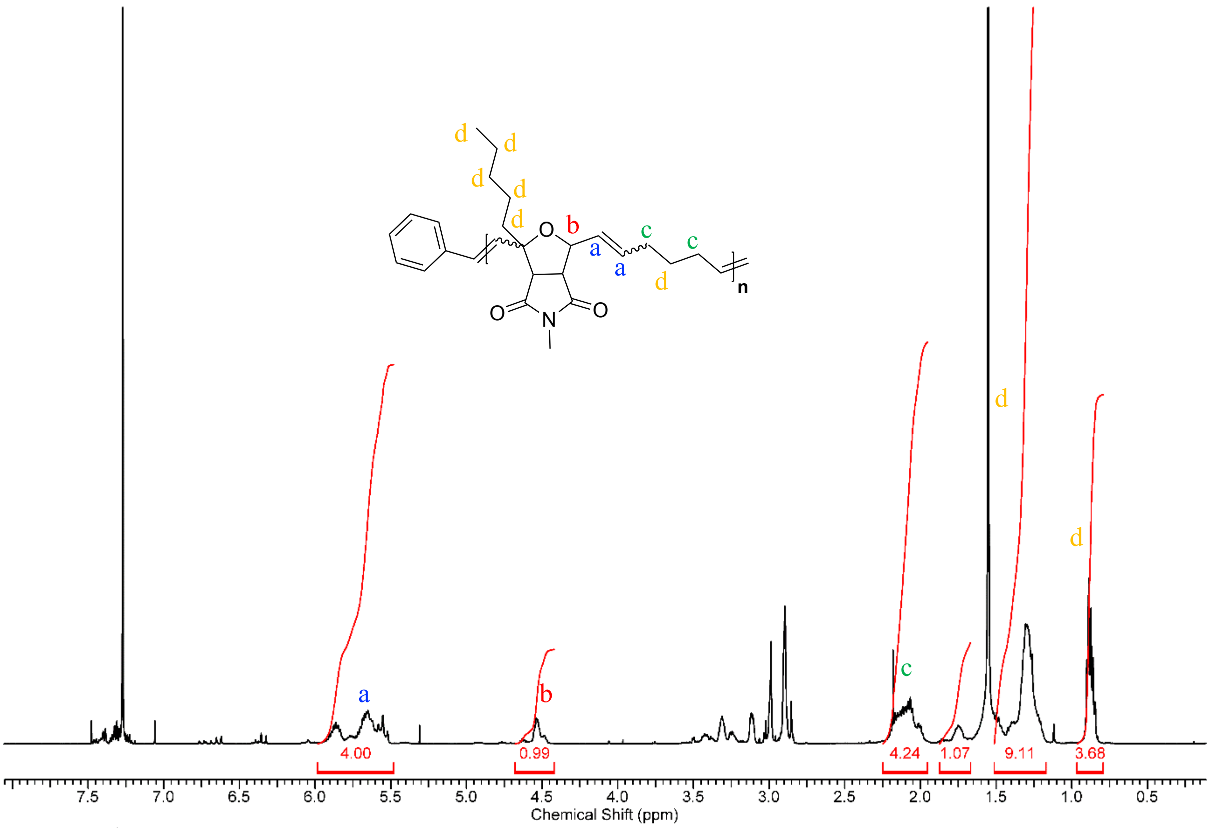

Fig S87. ^{1}H NMR (chloroform-d, 400 MHz) spectrum of Poly **P12.**

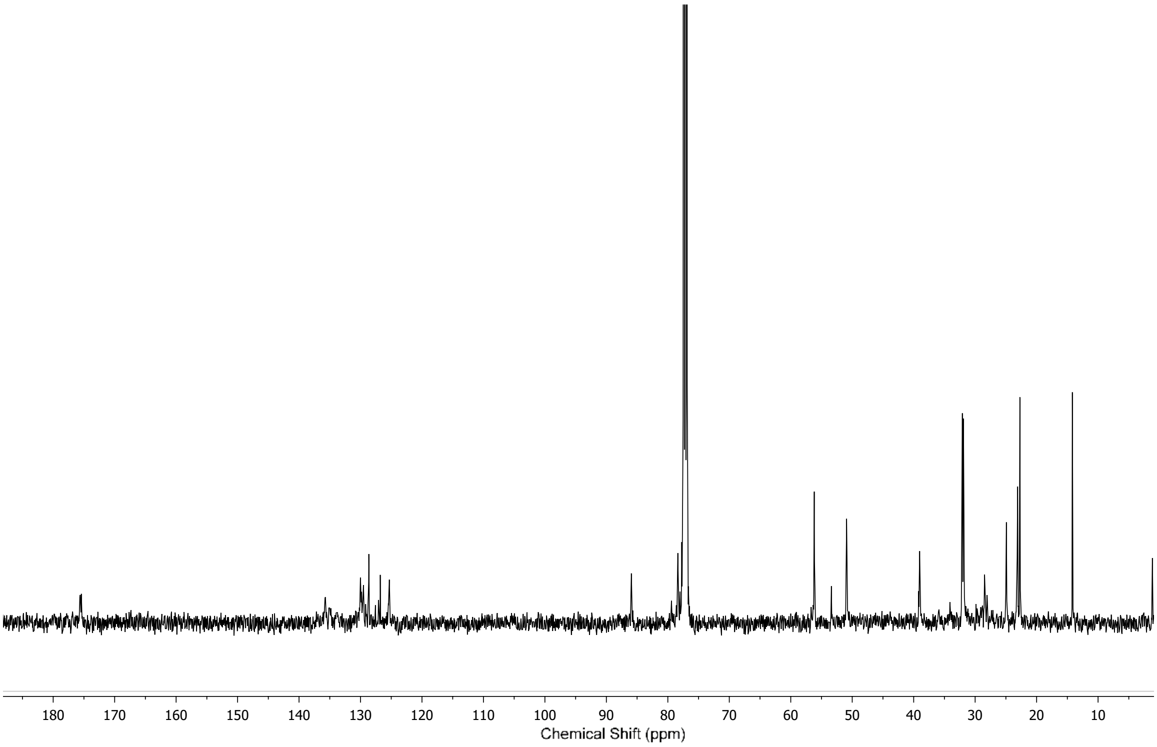

Fig S88. ^{13}C NMR (chloroform-d, 101 MHz) spectrum of Poly **P12.**

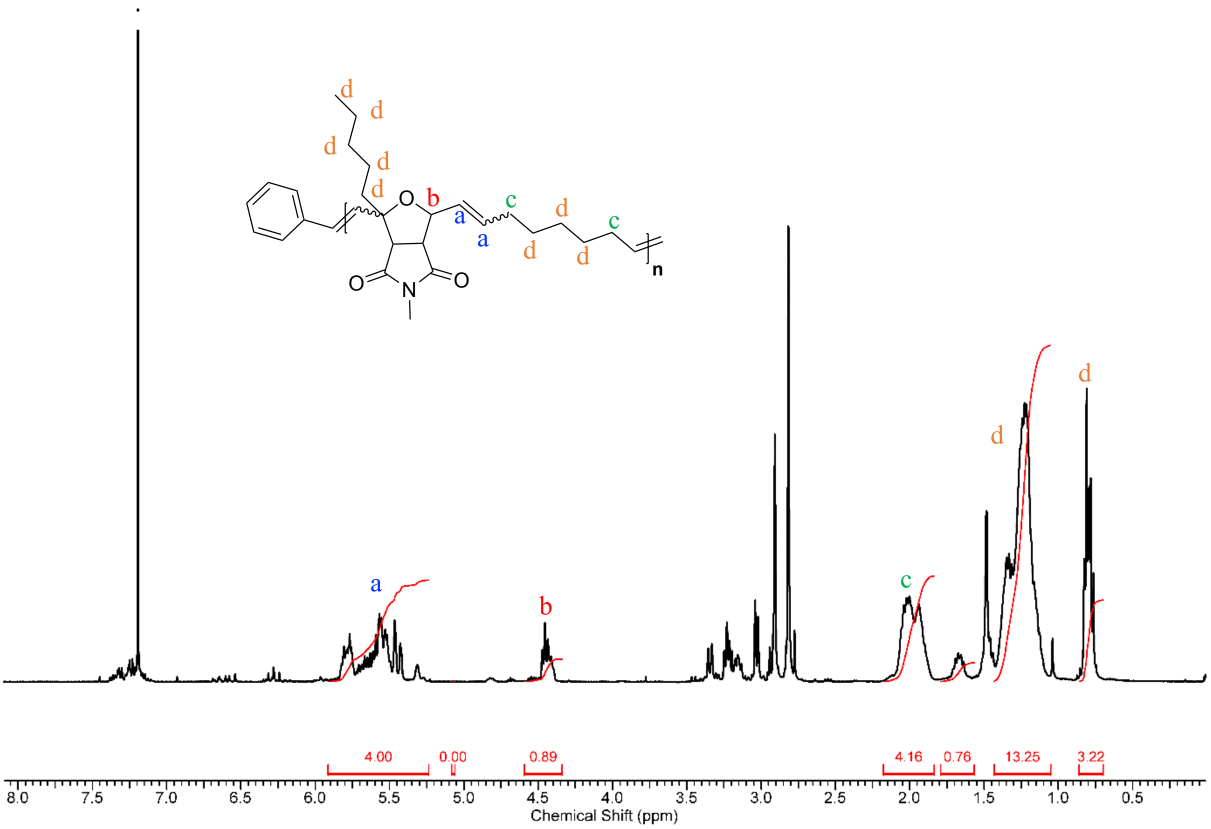

Fig S89. ^{1}H NMR (chloroform-d, 400 MHz) spectrum of Poly **P13.**

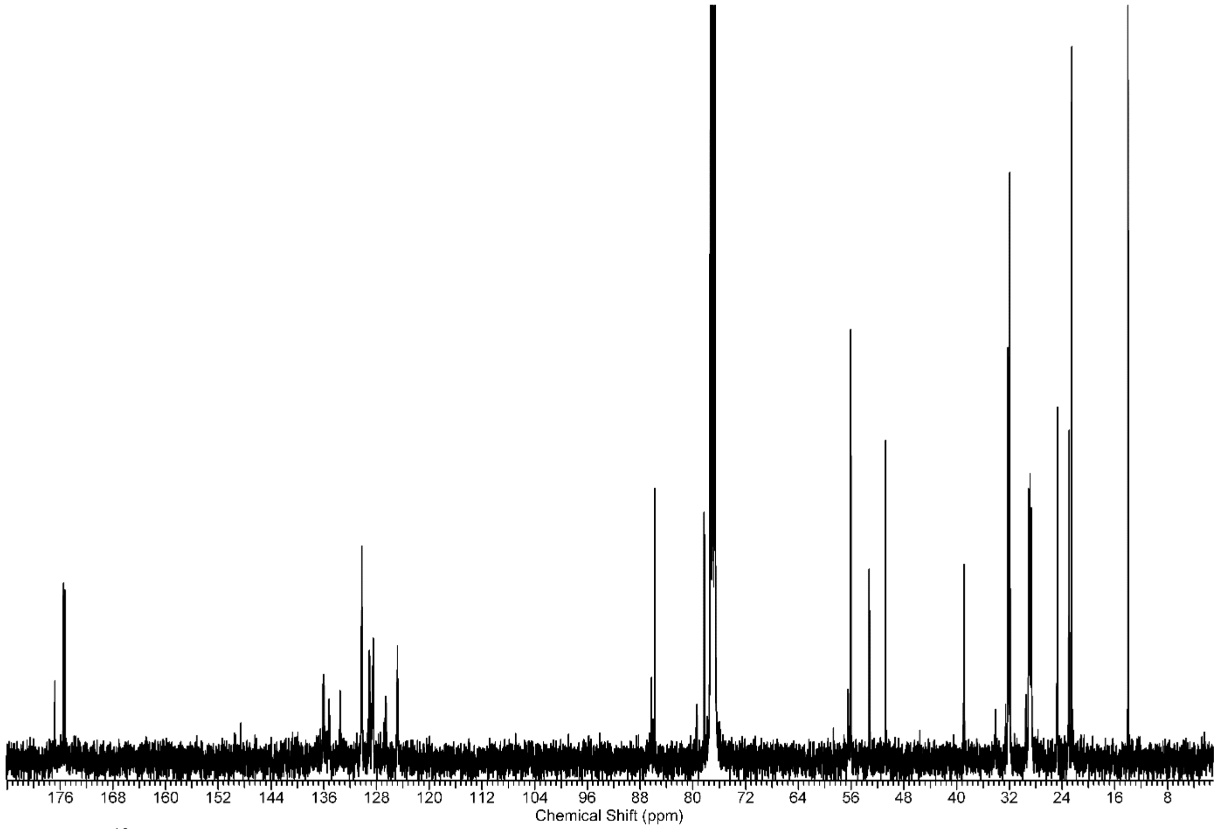

Fig S90. ^{13}C NMR (chloroform-d, 101 MHz) spectrum of Poly **P13.**

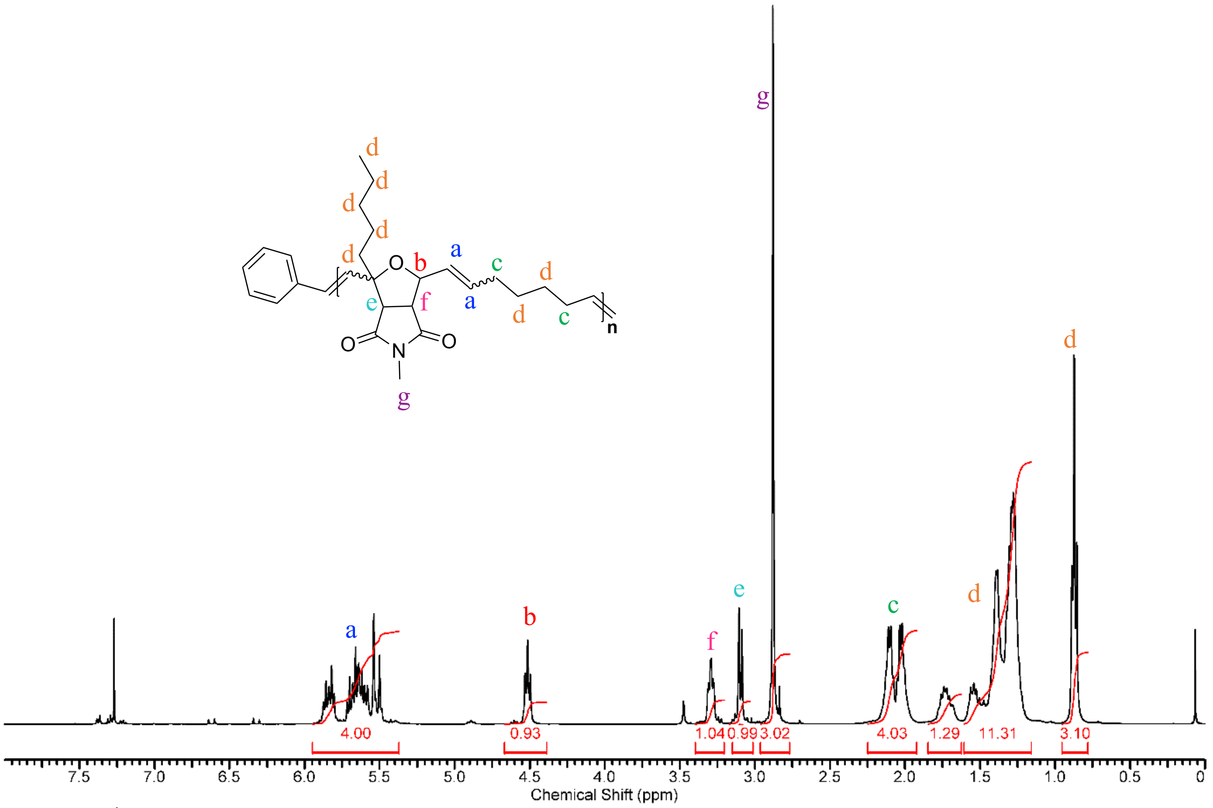

Fig S91. ^{1}H NMR (chloroform-d, 400 MHz) spectrum of Poly **P14.**

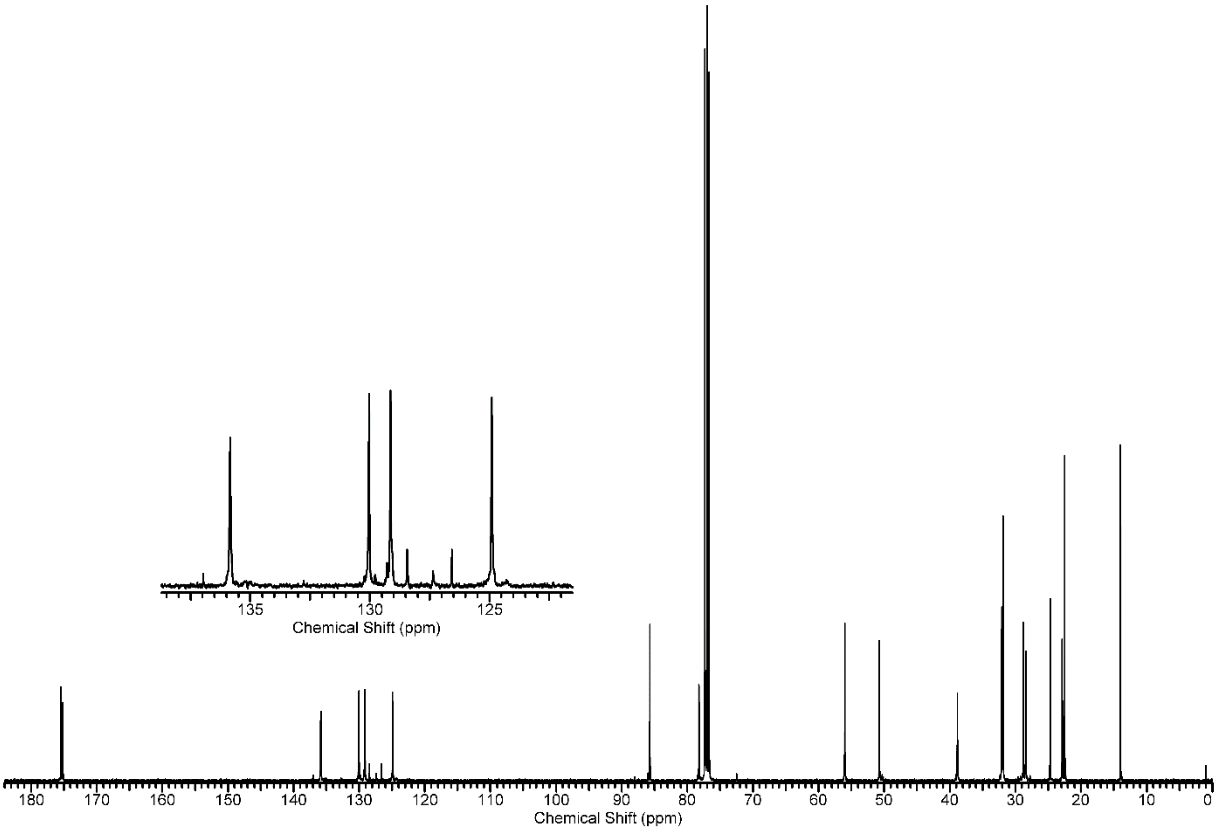

Fig S92. ^{13}C NMR (chloroform-d, 101 MHz) spectrum of Poly **P14.**

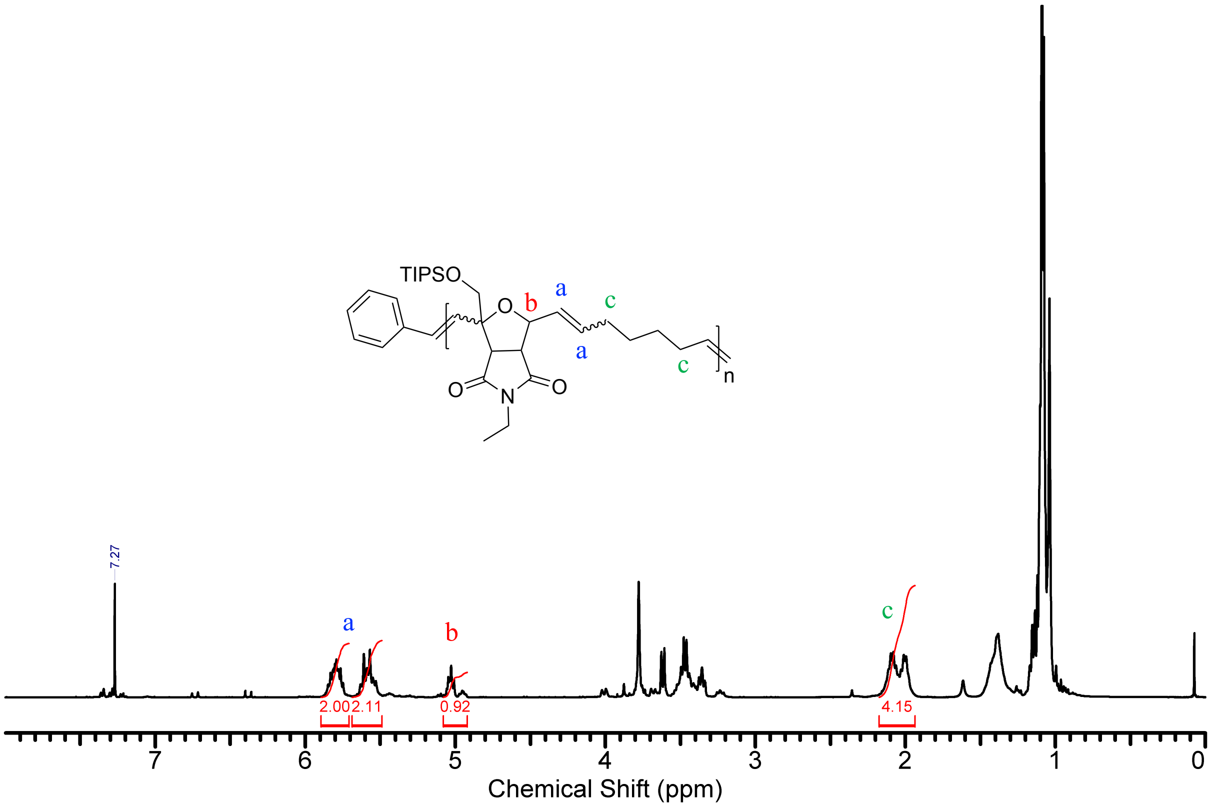

Fig S93. ^{1}H NMR (chloroform-d, 400 MHz) spectrum of Poly **P15.**

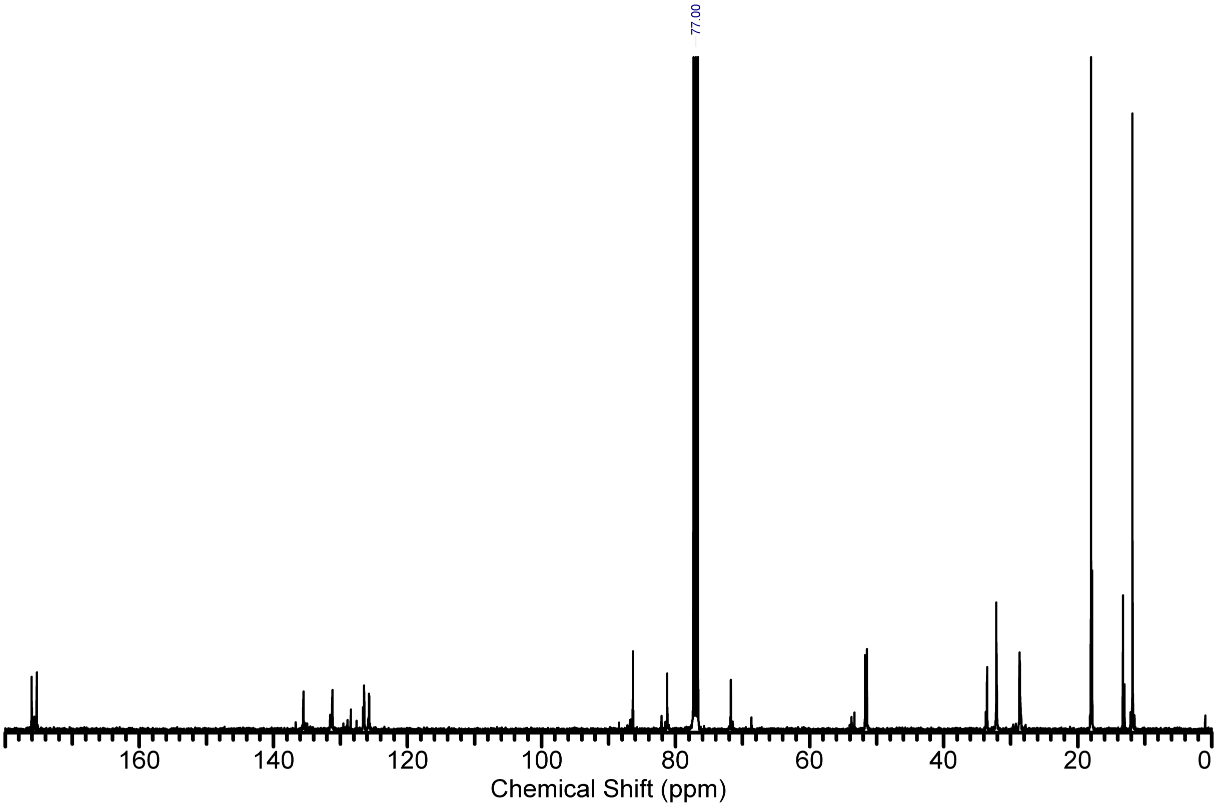

Fig S94. ^{13}C NMR (chloroform-d, 101 MHz) spectrum of Poly **P15.**

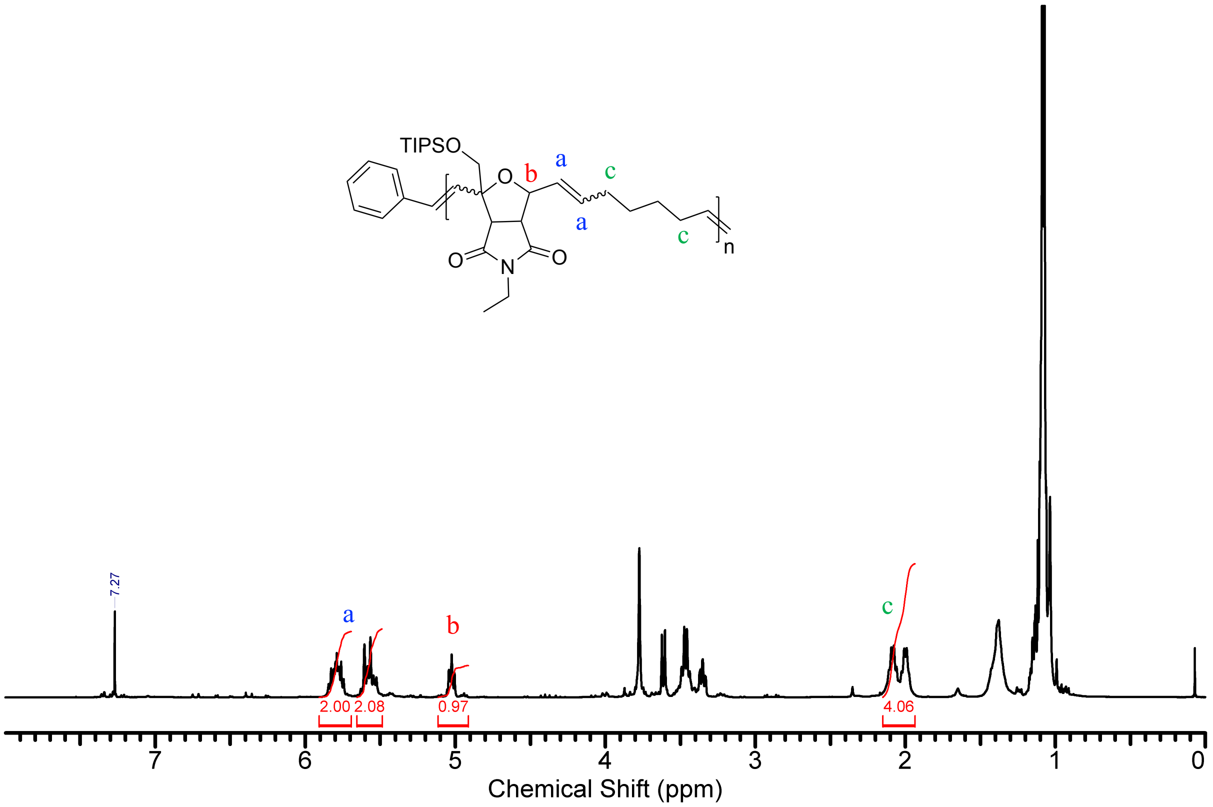

Fig S95. ^{1}H NMR (chloroform-d, 400 MHz) spectrum of Poly **P16.**

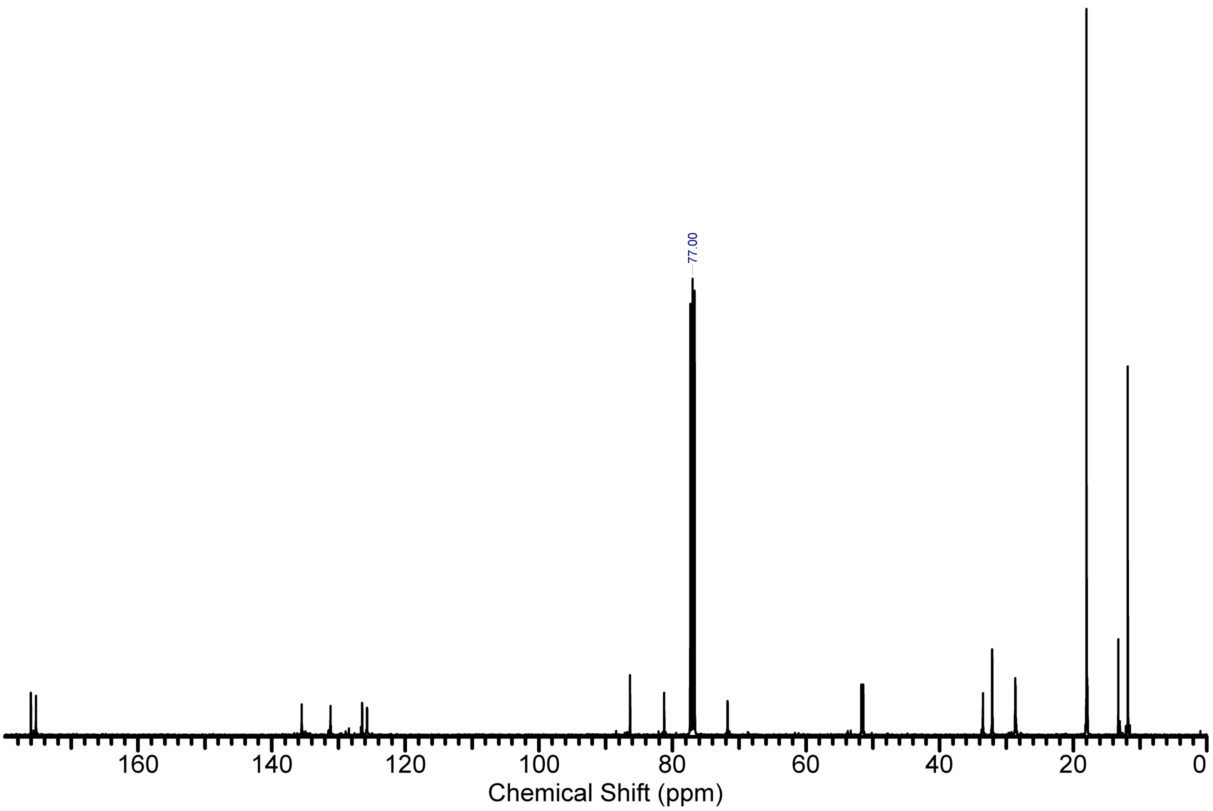

Fig S96. ^{13}C NMR (chloroform-d, 101 MHz) spectrum of Poly **P16.**

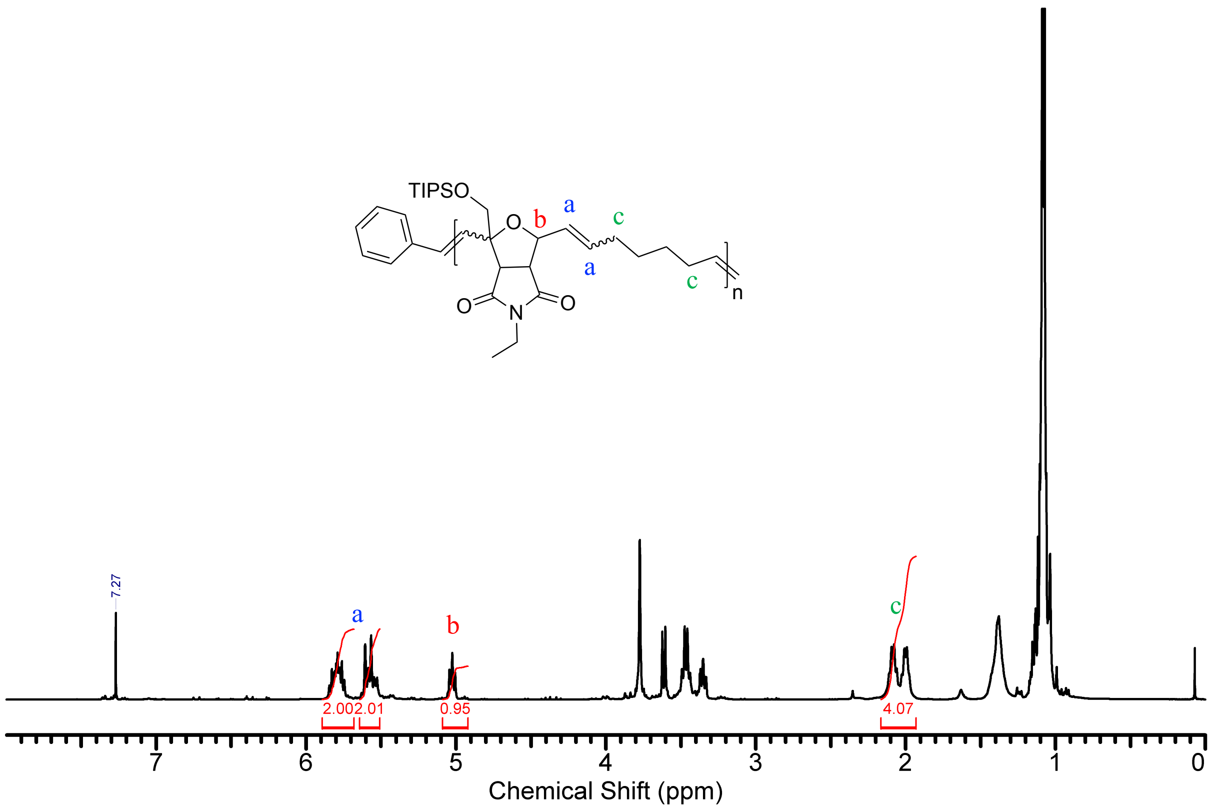

Fig S97. ^{1}H NMR (chloroform-d, 400 MHz) spectrum of Poly **P17.**

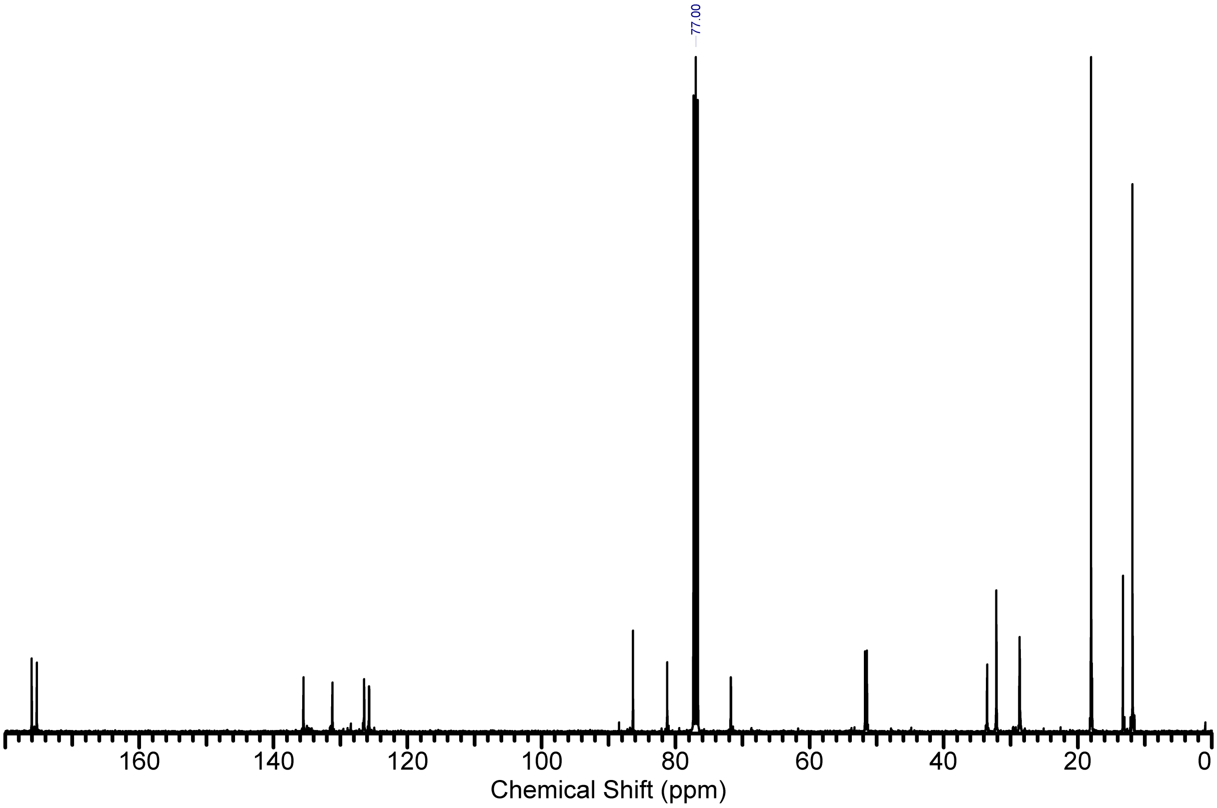

Fig S98. ^{13}C NMR (chloroform-d, 101 MHz) spectrum of Poly **P17.**

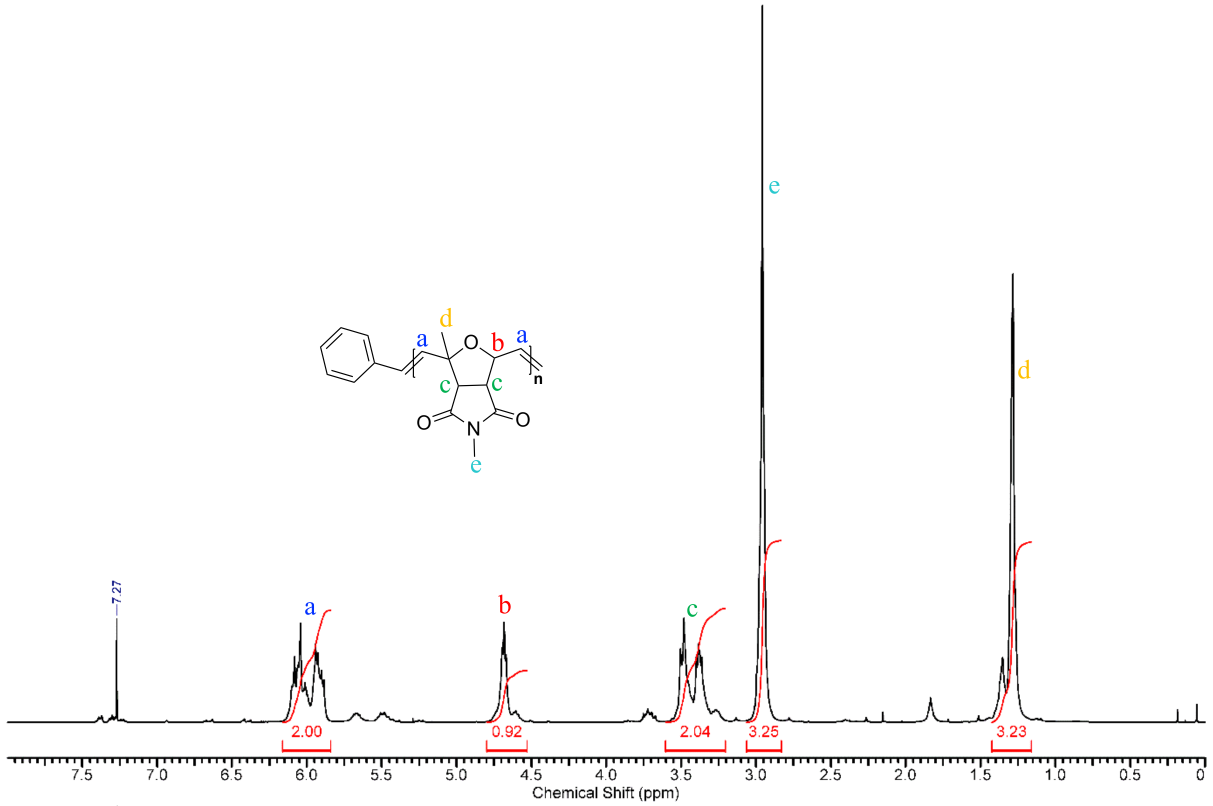

Fig S99. ^{1}H NMR (chloroform-d, 400 MHz) spectrum of homopolymer of **2-exo.**

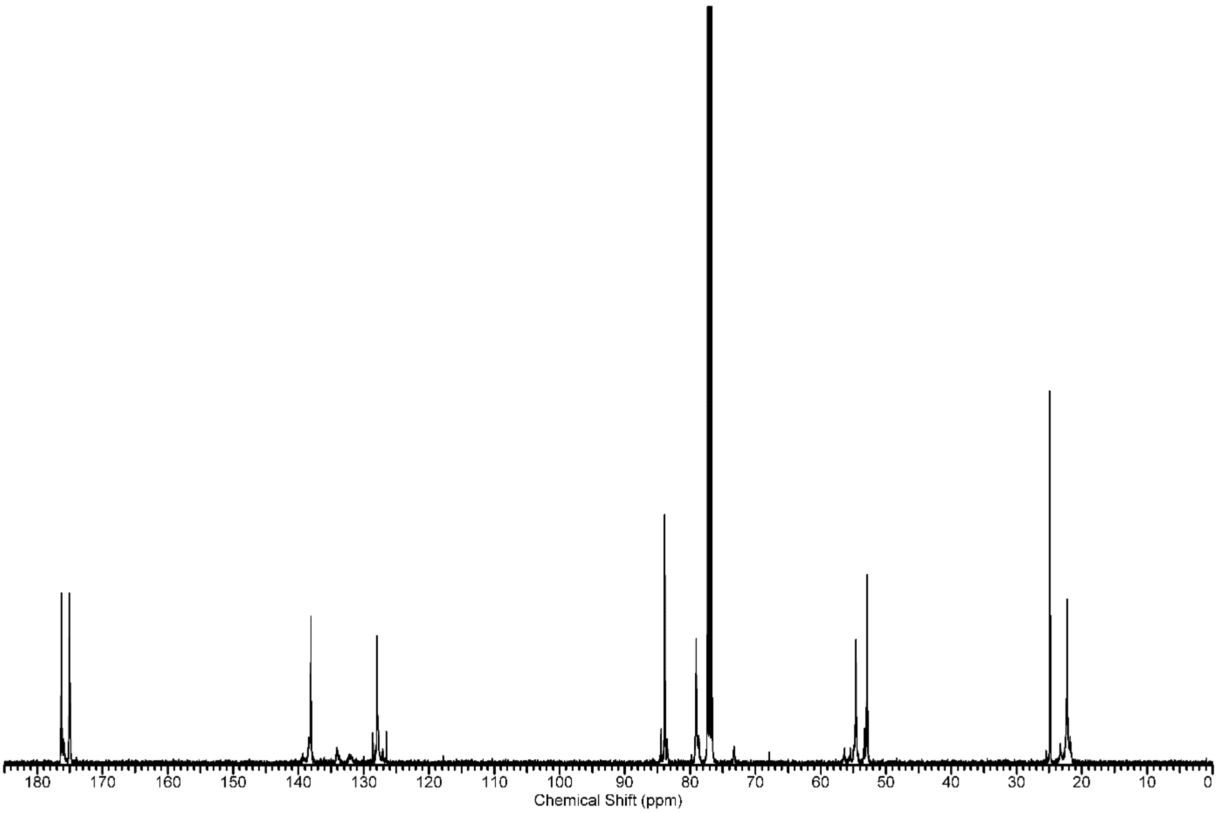

Fig S100. ^{13}C NMR (chloroform-d, 101 MHz) spectrum of homopolymer of **2-exo.**

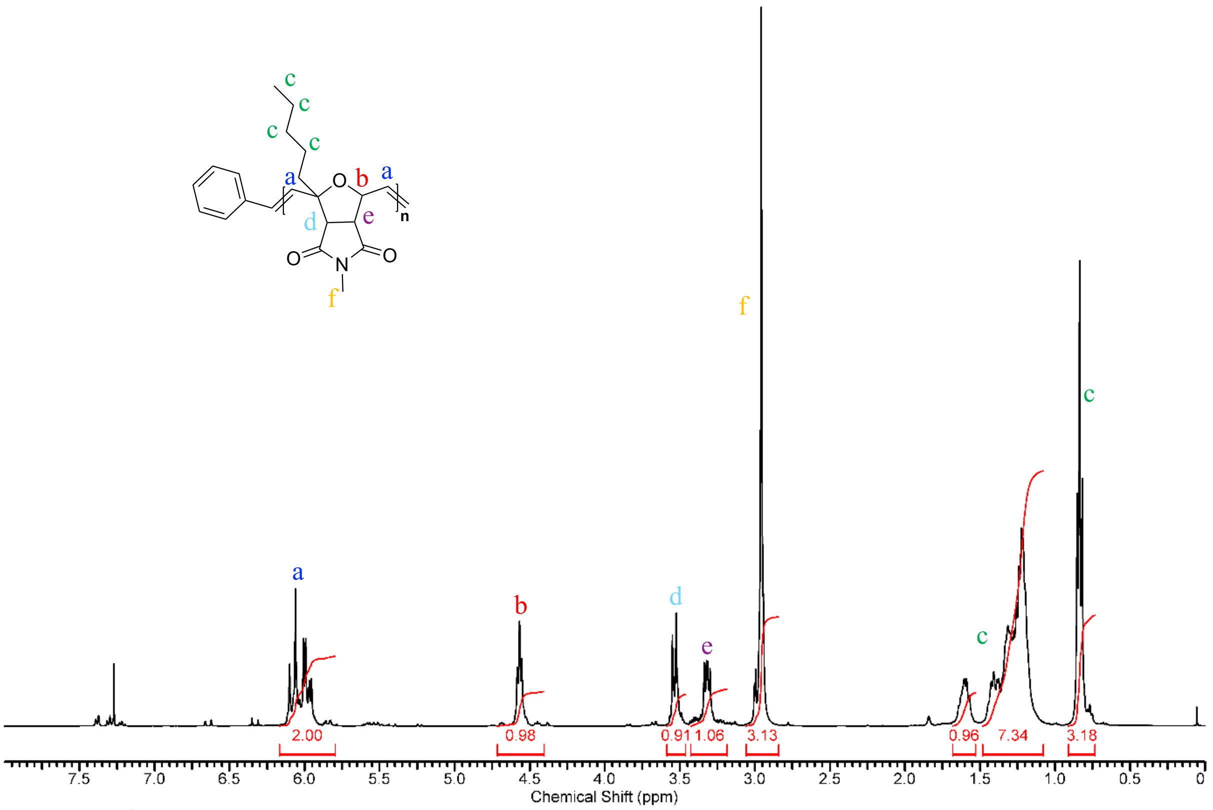

Fig S101. ^{1}H NMR (chloroform-d, 400 MHz) spectrum of homopolymer of **4-exo.**

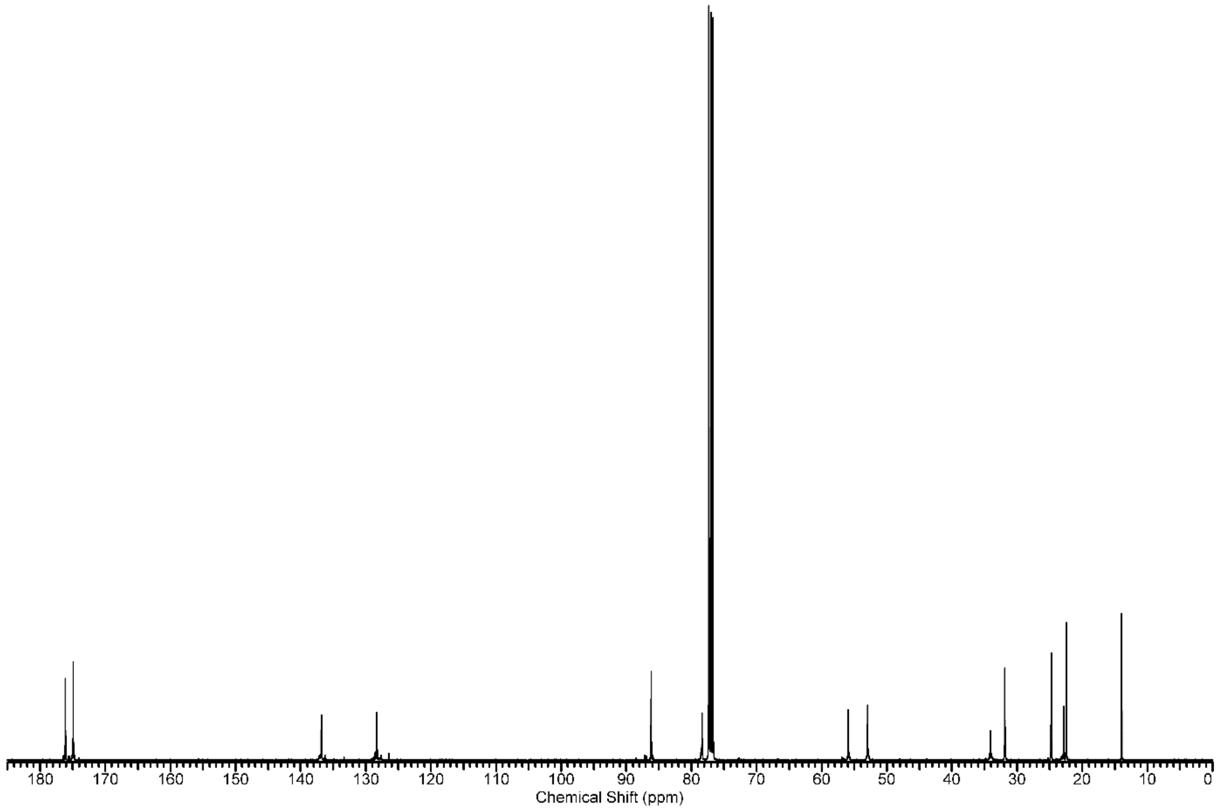

Fig S102. ^{13}C NMR (chloroform-d, 101 MHz) spectrum of homopolymer of **4-exo.**

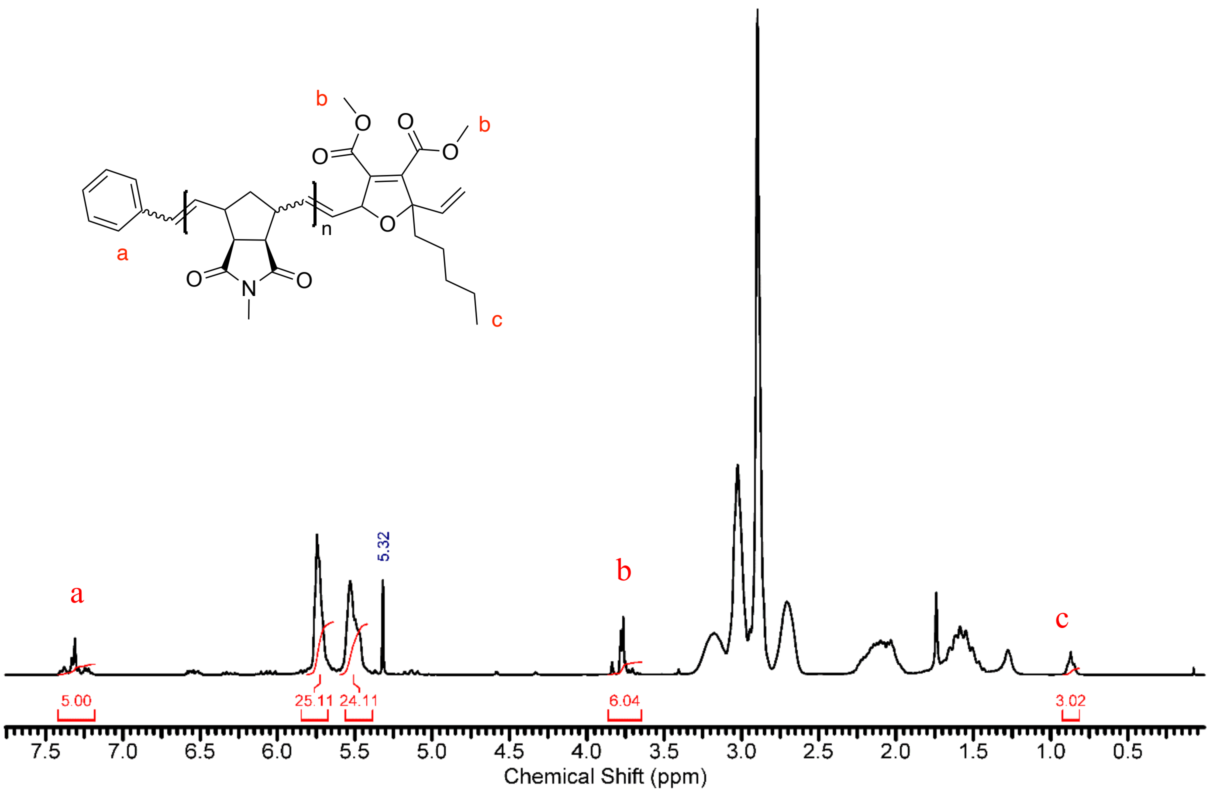

Fig S103. ^{1}H NMR (DCM-d2, 400 MHz) spectrum of **P18.**

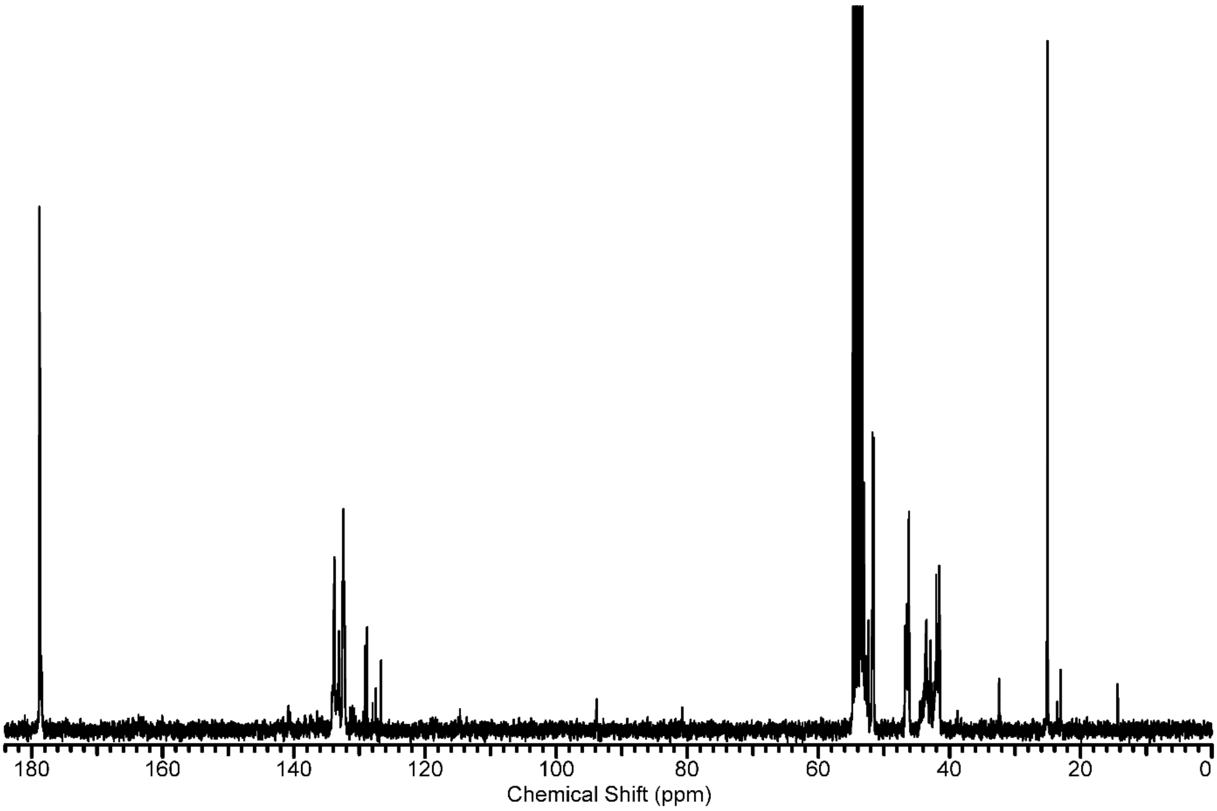

Fig S104. ^{13}C NMR (DCM-d2, 101 MHz) spectrum of **P18.**

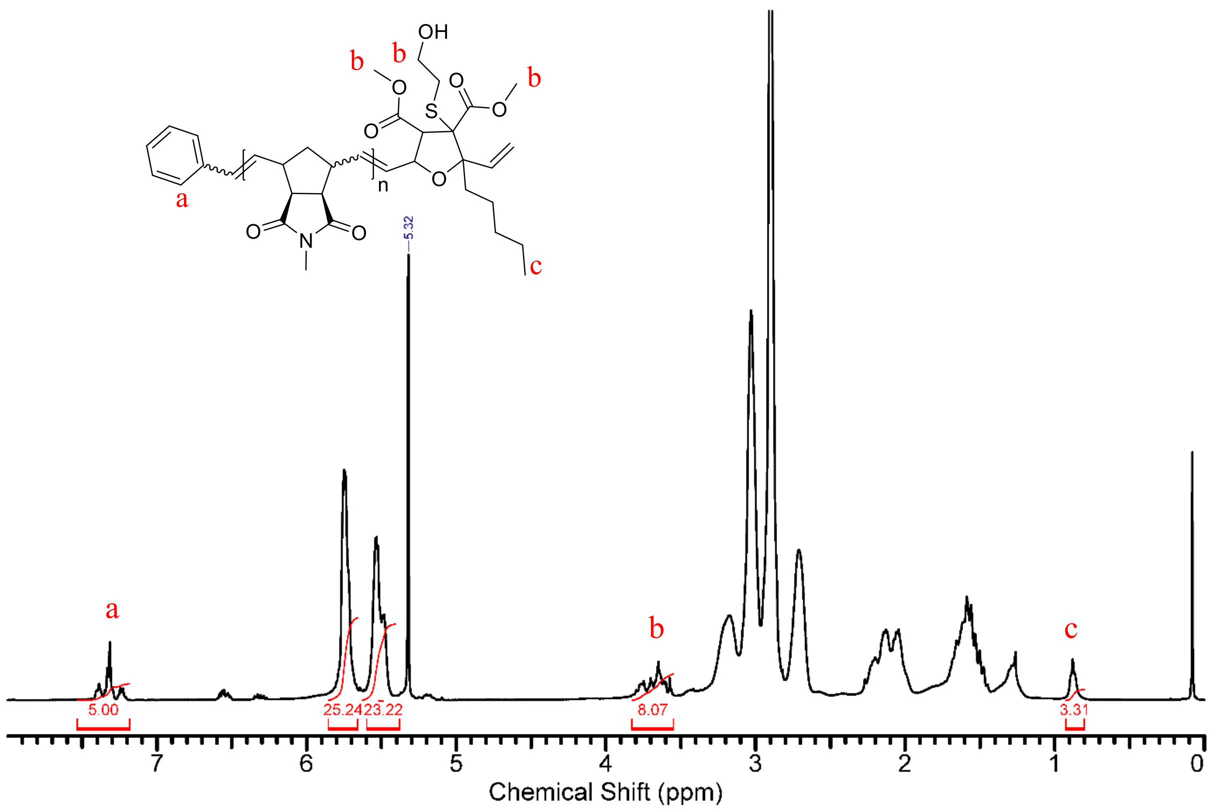

Fig S105. ^{1}H NMR (DCM-d2, 400 MHz) spectrum of **P19.**

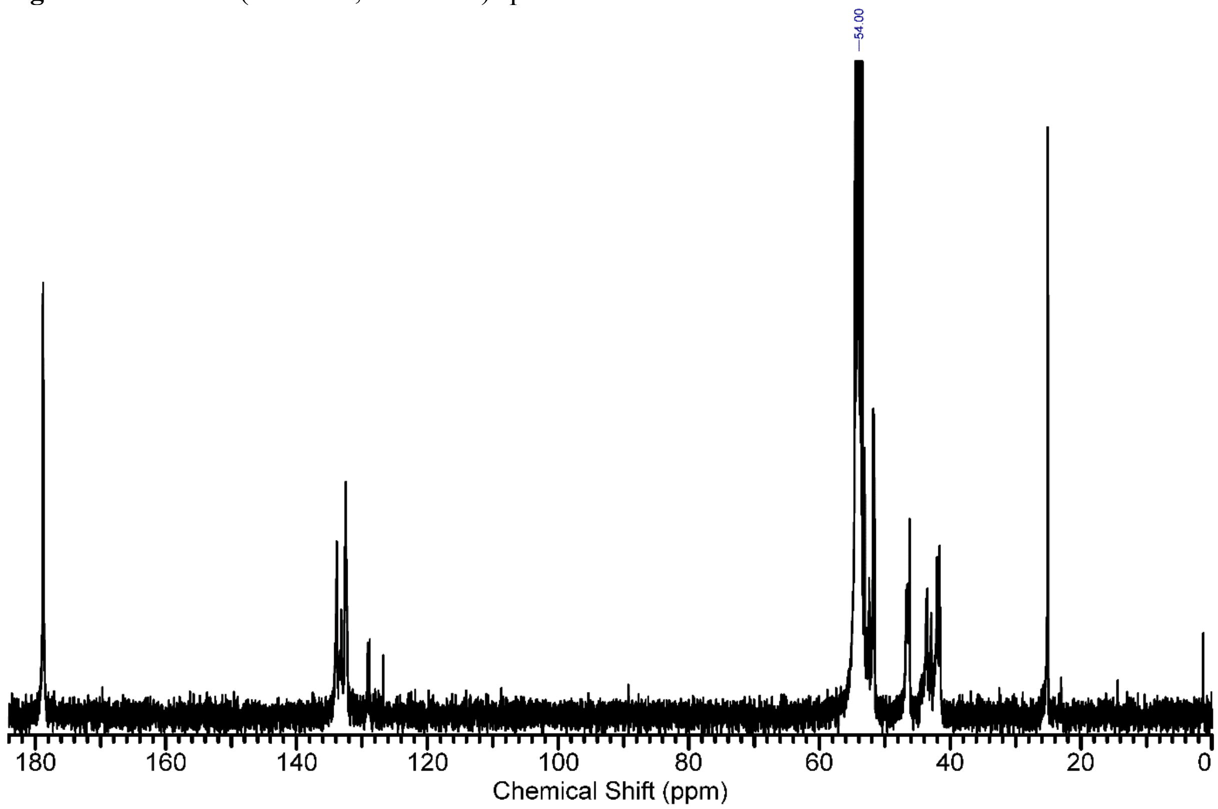

Fig S106. ^{13}C NMR (DCM-d2, 101 MHz) spectrum of **P19.**

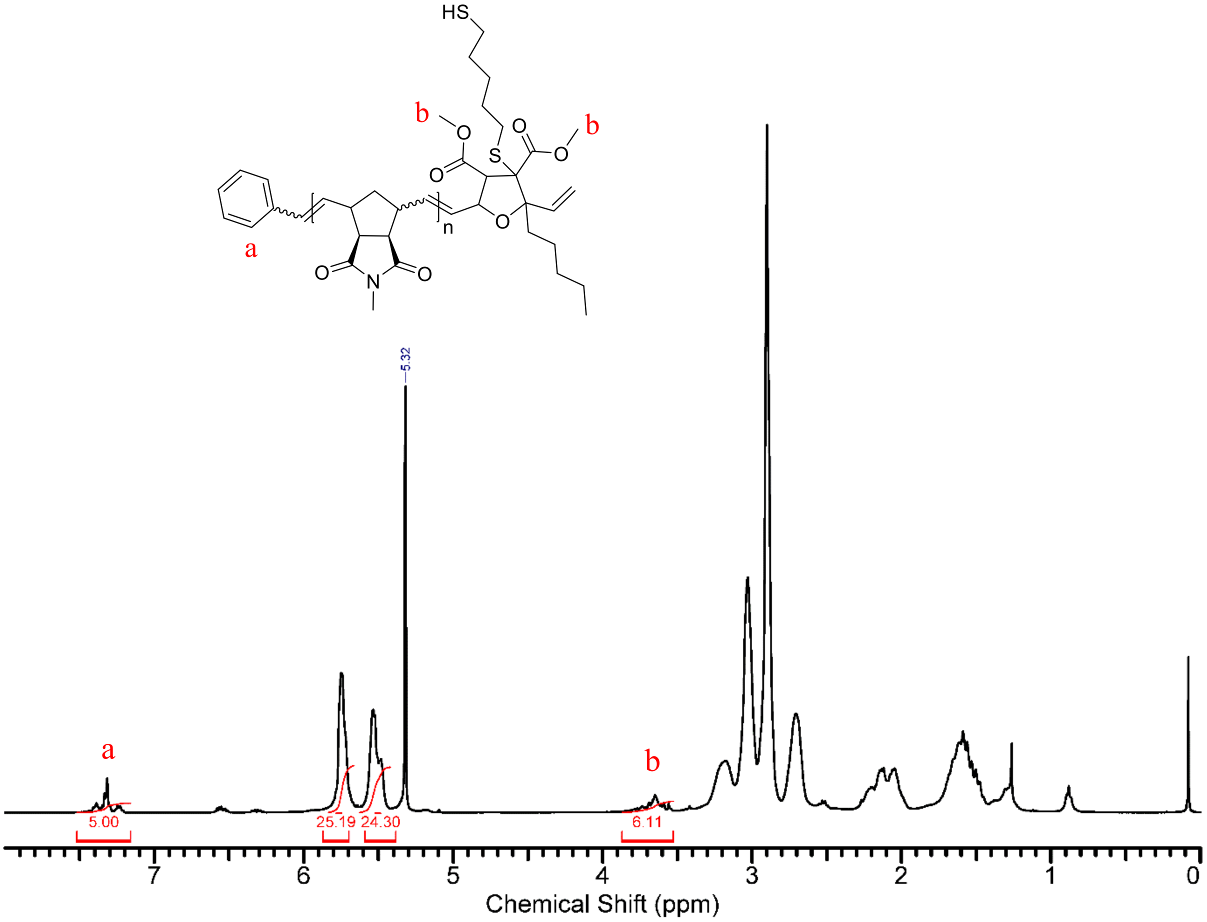

Fig S107. ^{1}H NMR (DCM-d2, 400 MHz) spectrum of **P20.**

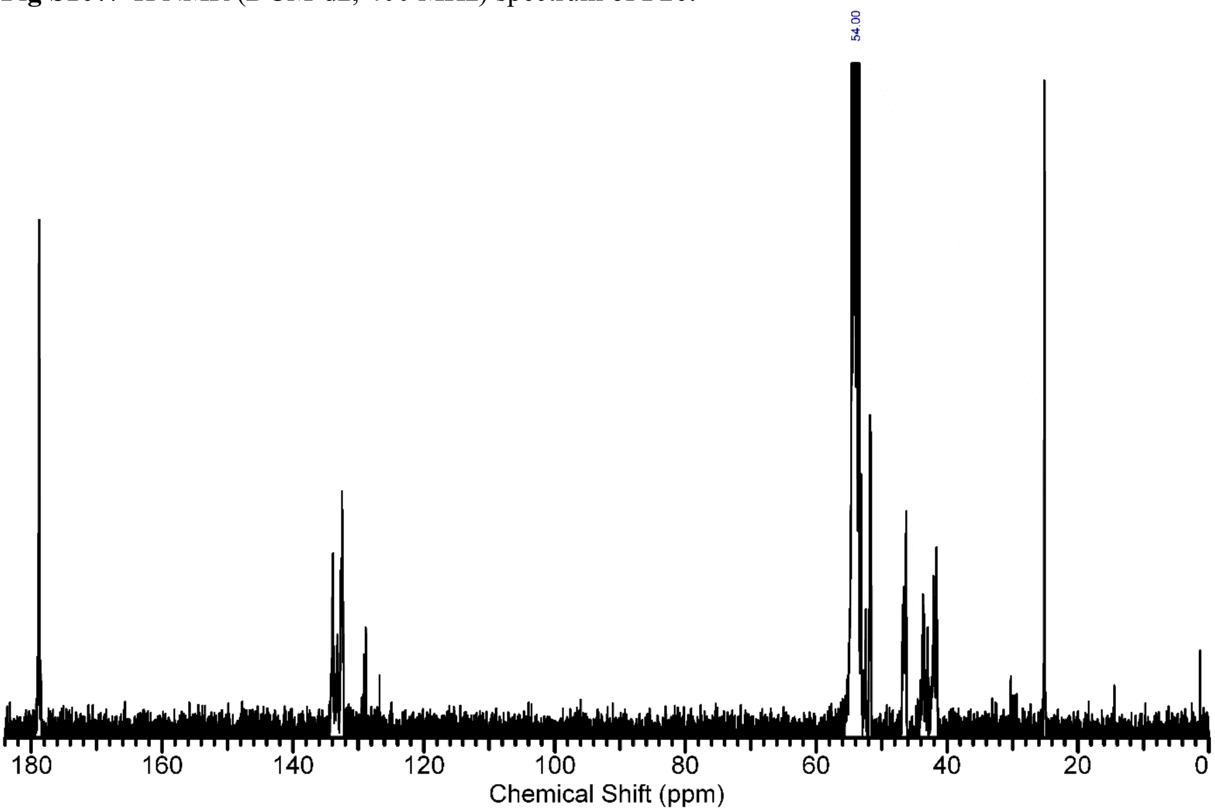

Fig S108. ^{13}C NMR (DCM-d2, 101 MHz) spectrum of **P20.**

NMR spectra comparisons of homo and alternating copolymer

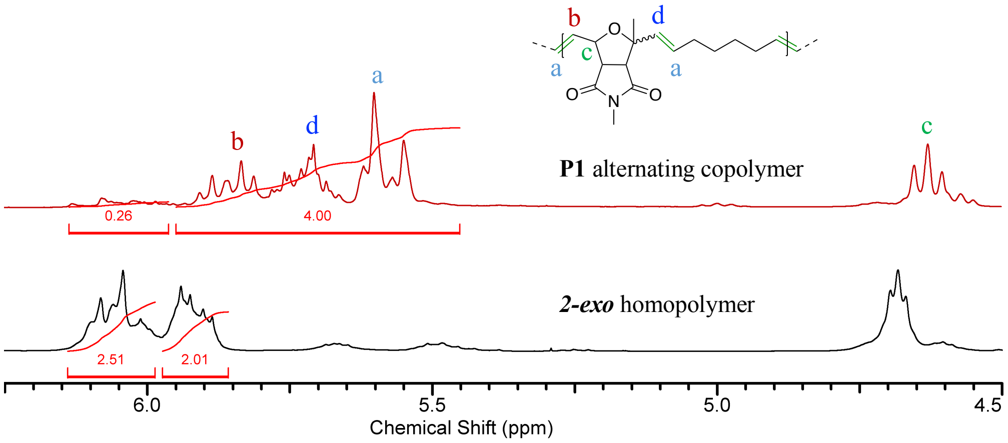

Fig S109. Stacking of ^{1}H NMR spectrum the alternating copolymer **P1** (90% alternating diads) synthesized from endo/exo mixture (**2** + cyclohexene) and homopolymer of **2-exo**.

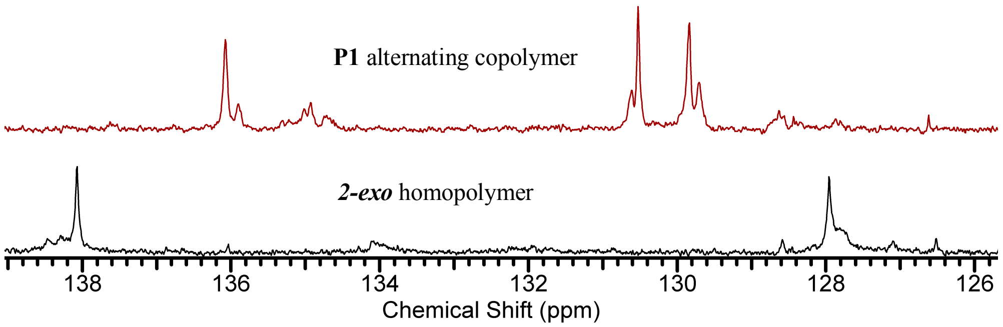

Fig S110. Stacking of ^{13}C NMR spectrum of the alternating copolymer **P1** synthesized from endo/exo mixture (**2** + cyclohexene) and homopolymer of **2-exo.**

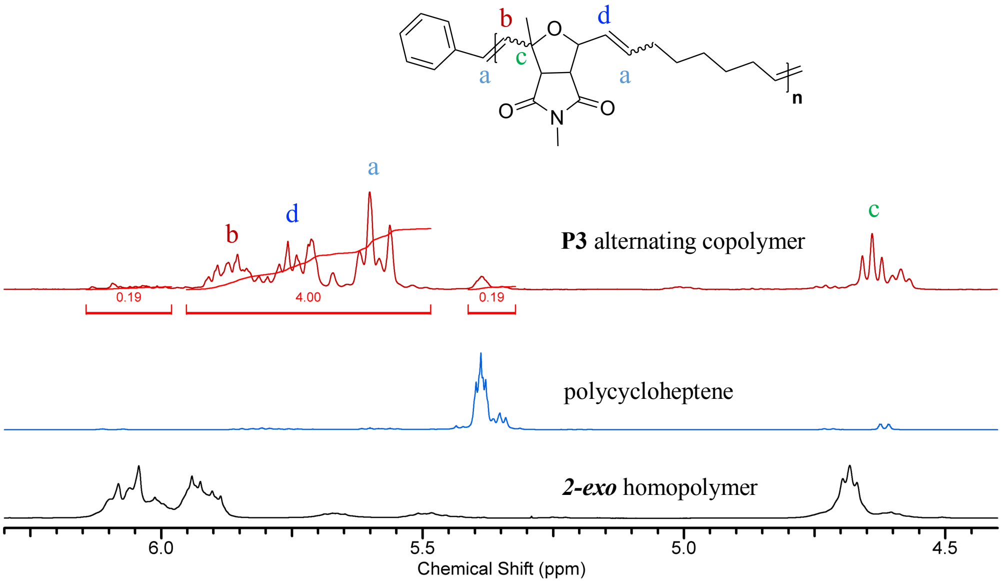

Fig S111. Stacking of ^{1}H NMR spectrum the alternating copolymer **P3** (92% alternating diads) synthesized from endo/exo mixture (**2** + cycloheptene), homopolymer of **2-exo** and homopolymer of cycloheptene.

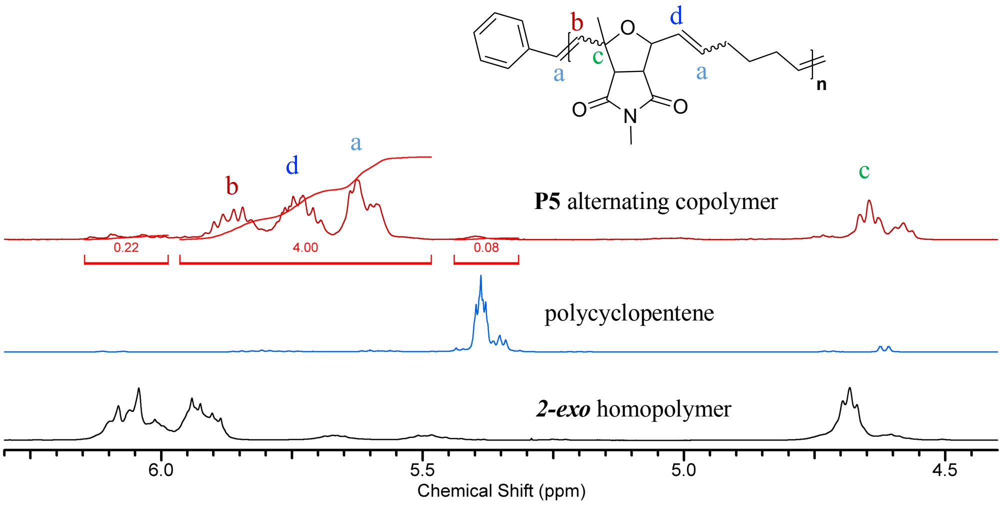

Fig S112. Stacking of ^{1}H NMR spectrum the alternating copolymer **P5** (92% alternating diads) synthesized from endo/exo mixture (**2** + cyclopentene), homopolymer of **2-exo** and homopolymer of cyclopentene.

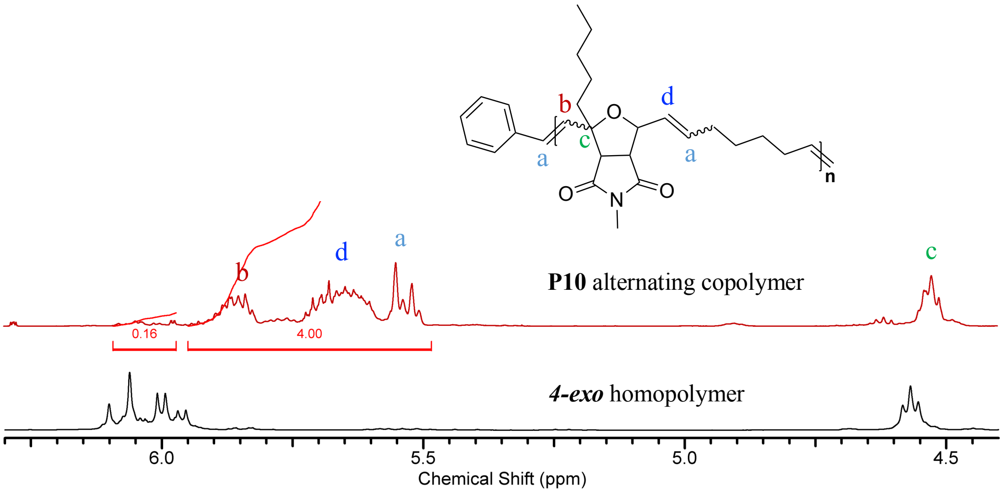

Fig S113. Stacking of ^{1}H NMR spectrum the alternating copolymer **P10** (96% alternating diads) synthesized from endo/exo mixture (**4** + cyclohexene), homopolymer of **4-exo**.

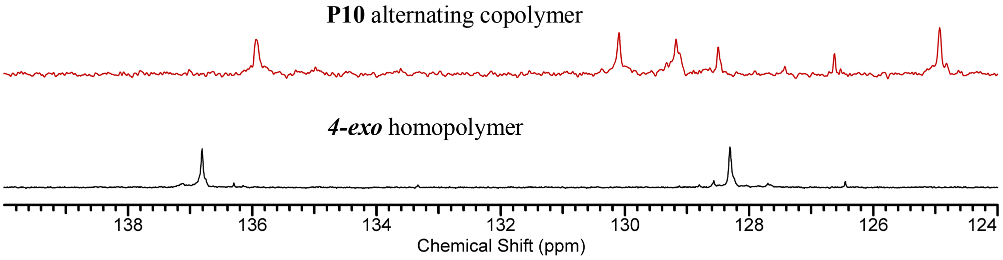

Fig S114. Stacking of ^{13}C NMR spectrum of the alternating copolymer **P10** synthesized from endo/exo mixture (**4** + cyclohexene) and homopolymer of **4-exo.**

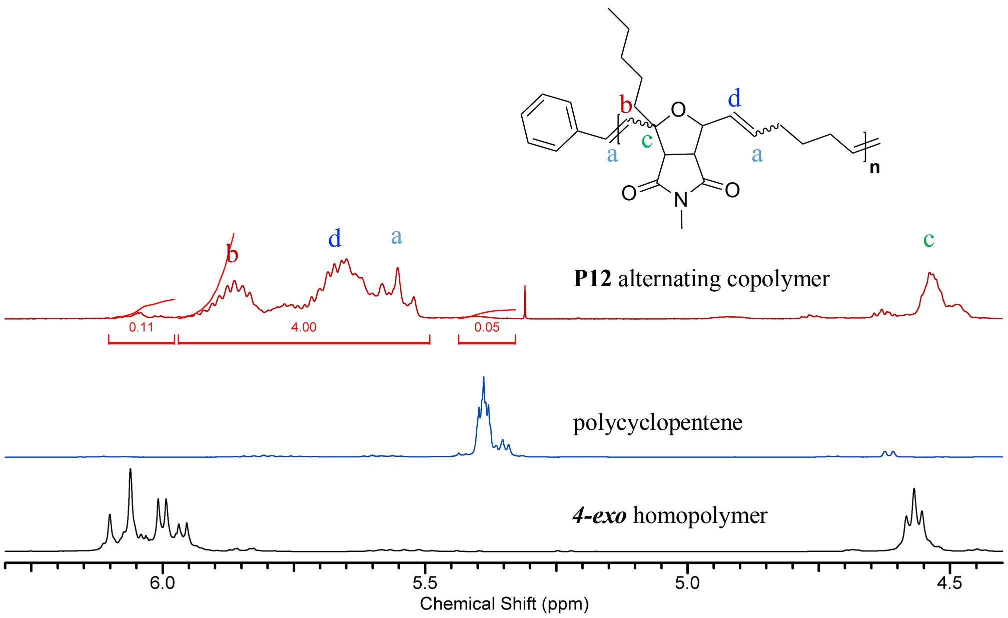

Fig S115. Stacking of ^{1}H NMR spectrum the alternating copolymer **P12** (96% alternating diads) synthesized from endo/exo mixture (**4** + cyclopentene), homopolymer of **4-exo** and homopolymer of cyclopentene.

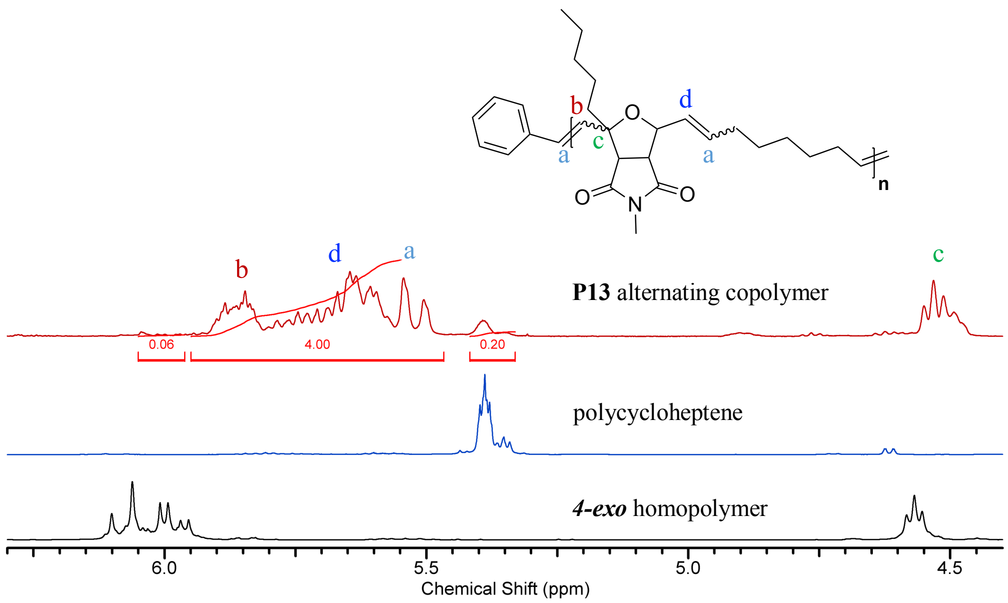

Fig S116. Stacking of ^{1}H NMR spectrum the alternating copolymer **P13** (94% alternating diads) synthesized from endo/exo mixture (**4** + cycloheptene), homopolymer of **4-exo** and homopolymer of cycloheptene.

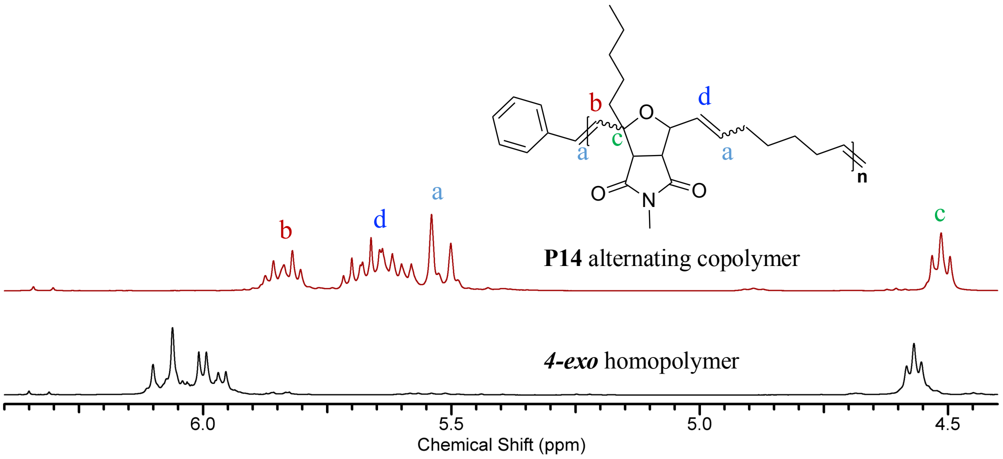

Fig S117. Stacking of ^{1}H NMR spectrum the alternating copolymer **P14** (strictly alternating) synthesized from pure (**4-endo** + cyclohexene), homopolymer of **4-exo**.

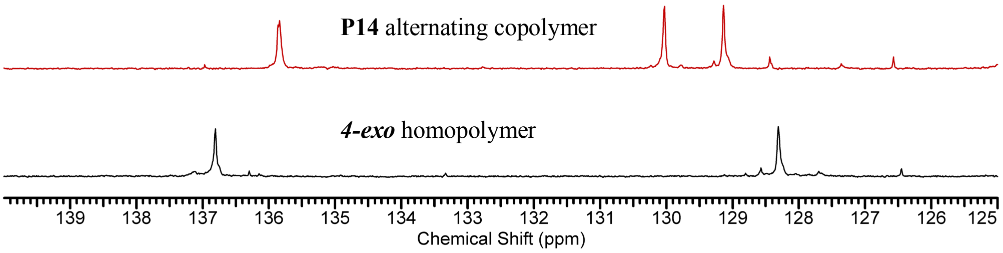

Fig S118. Stacking of ^{13}C NMR spectrum the alternating copolymer **P14** (strictly alternating) synthesized from pure (**4-endo** + cyclohexene), homopolymer of **4-exo**.

High-resolution mass spectrometric data

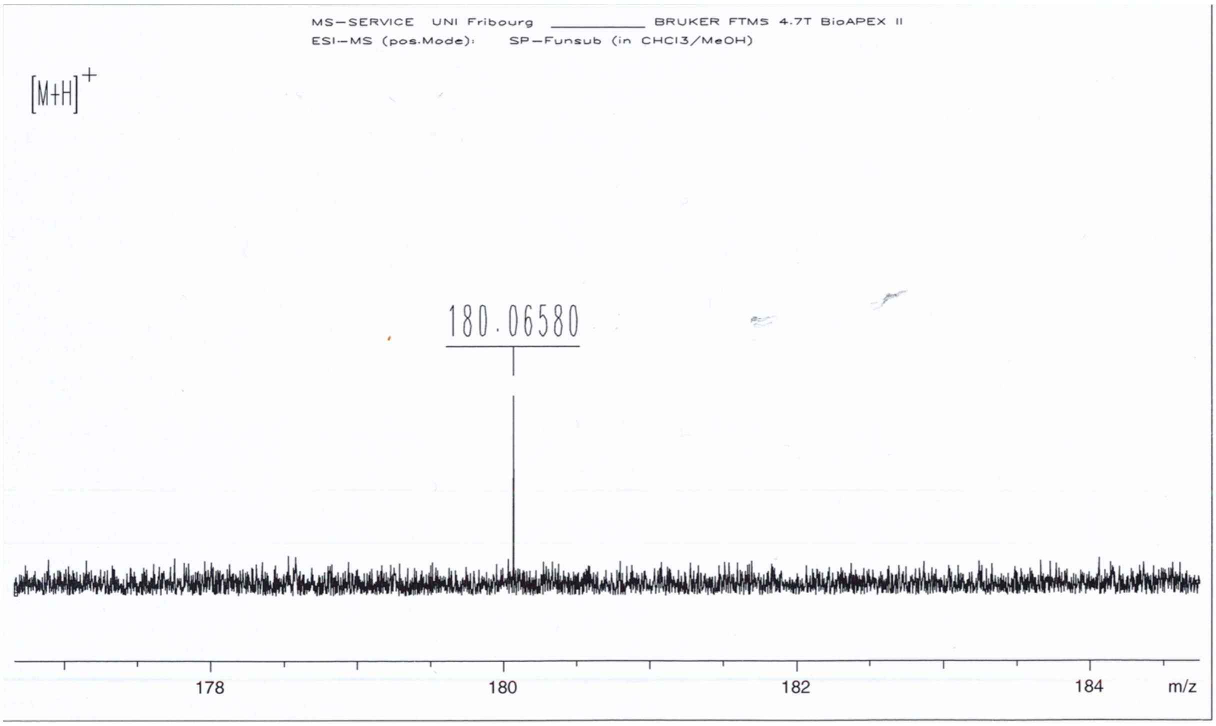

Fig S119. HR-MS of monomer **1**.

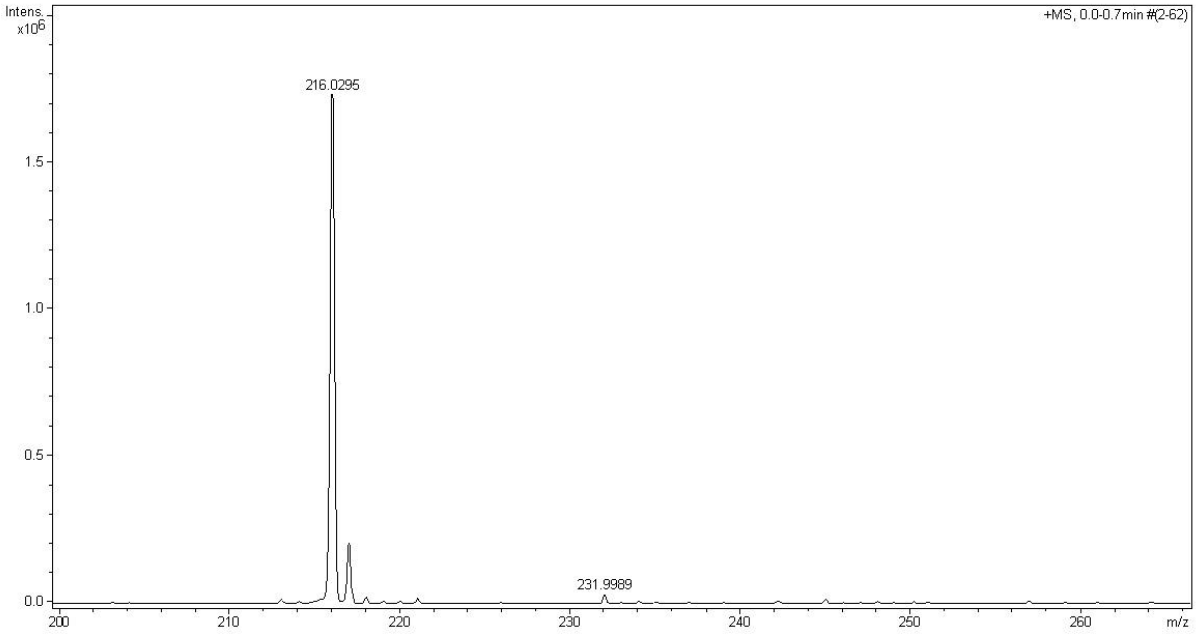

Fig S120. ESI-MS of monomer **2**.

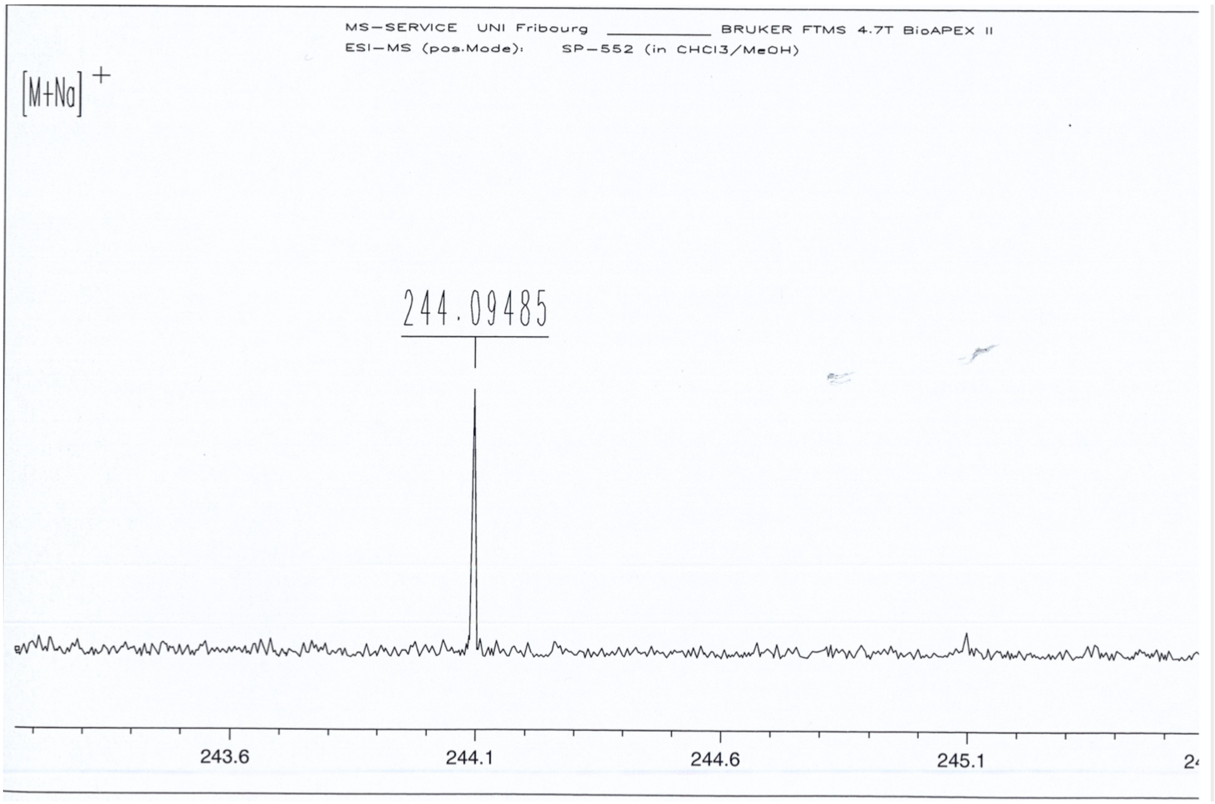

Fig S121. HR-MS of monomer **3**.

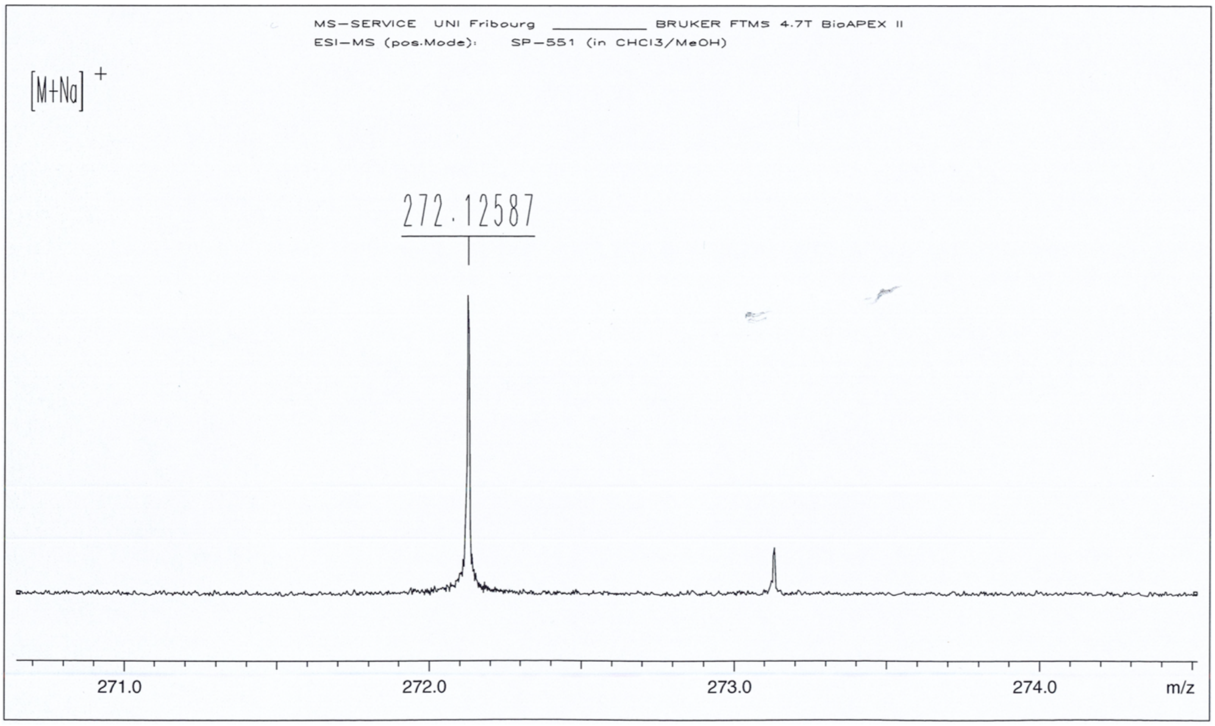

Fig S122. HR-MS of monomer **4**.

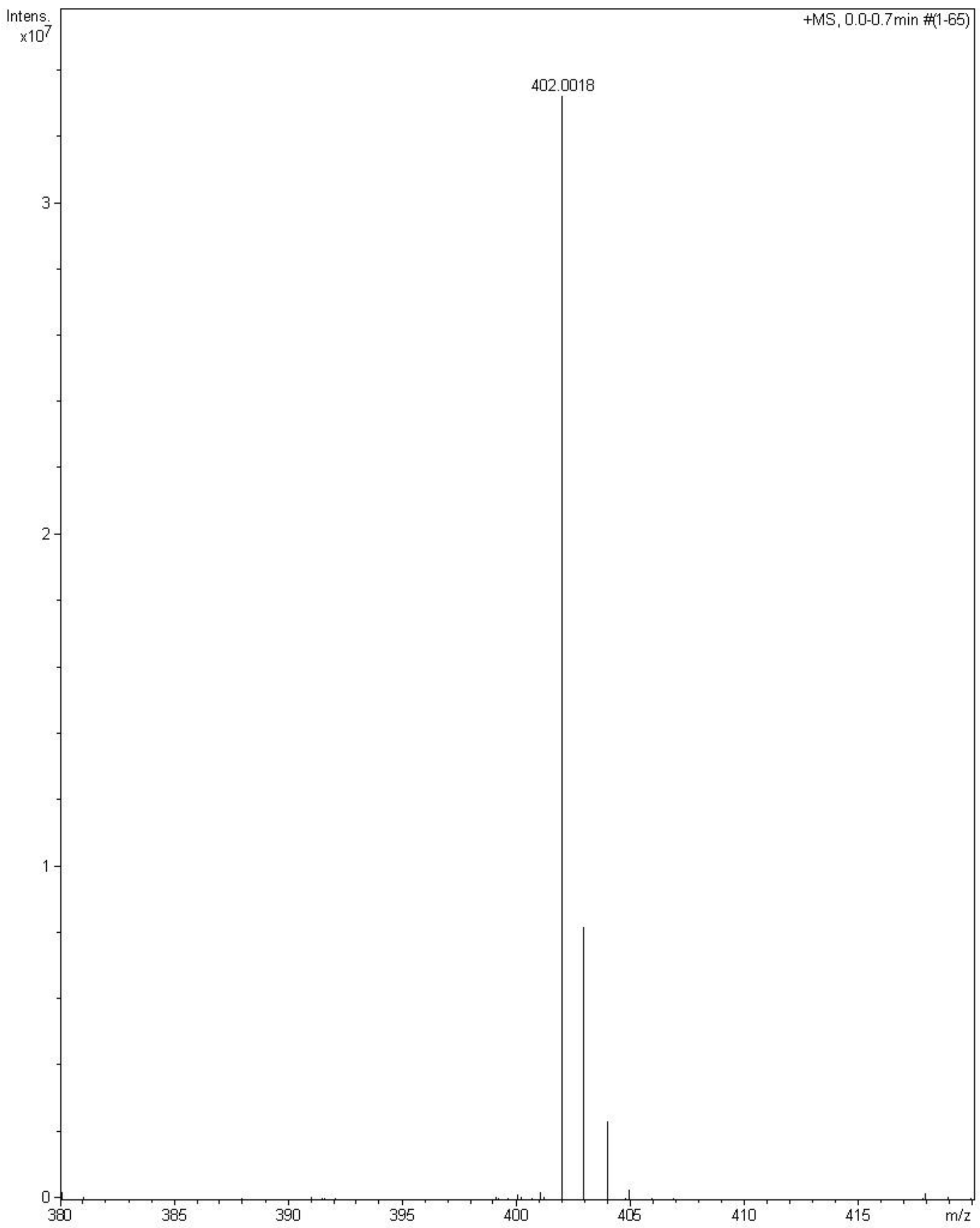

Fig S123. ESI-MS of monomer **5**.

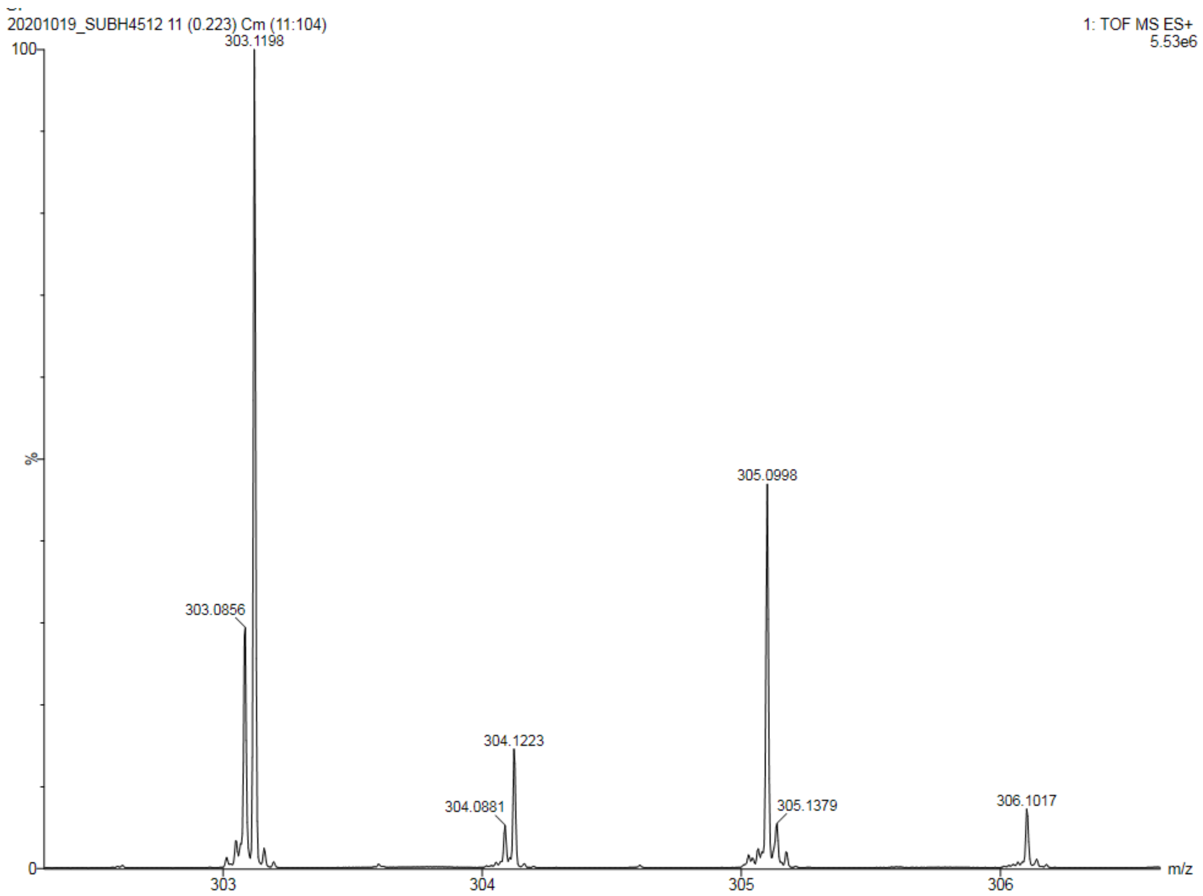

Fig S124. HR-MS of monomer **6**.
